# Beyond the rules: an integrative review of parental perspectives on safer infant sleep in shared environments

**DOI:** 10.3389/fpubh.2025.1629678

**Published:** 2025-09-15

**Authors:** Carly Grubb, Jeanine Young, Terri Downer, Levita D’Souza

**Affiliations:** ^1^School of Health, University of the Sunshine Coast, Murrumba Downs, QLD, Australia; ^2^School of Educational Psychology and Counselling, Monash University, Melbourne, VIC, Australia

**Keywords:** newborn health, public health interventions, maternal health, health disparities, sleep health, infant sleep safety, social determinants of health, sudden unexpected death in infancy

## Abstract

**Background:**

Despite public health campaigns promoting infant sleep safety, SUDI (including SIDS and fatal sleep accidents) remains one of the most significant contributors to post-neonatal infant death in many high-income countries. Bedsharing remains common despite predominant risk elimination guidelines, with many families struggling to follow rigid rules of avoidance. Risk minimisation considers the complexities of family life and recognises most infant deaths in shared sleep environments are associated with additional risk factors.

**Purpose and methods:**

Integrative review methodology was used to investigate the information parents need to minimise risk for infants under 12 months who share a sleep surface. Database searches included Scopus, CINAHL, PubMed, PsycNET and Emcare to identify peer-reviewed publications published January 2013–March 2025. Quality appraisal was undertaken using the QuADs tool.

**Results:**

A total of 60 articles met eligibility criteria. Twelve themes were generated from the data and grouped under four key domains: 1. Challenges in creating safer shared sleep environments, 2. Solutions/strategies used by parents to address challenges, 3. Family experiences when risk factors are present, and 4. Information needs of parents and caregivers. Families reported sharing sleep with infants, intentionally and accidentally, including those at a higher risk of SUDI. Bedsharing often occurs outside of a conscious parental ‘choice’, while families frequently refrain from disclosing bedsharing practices to health professionals. In the absence of formal guidance on safer shared sleep strategies, families generated their own solutions potentially increasing risk.

**Conclusion:**

Parents need universal access to non-judgmental, neutrally-worded support that allows them to ‘prepare to share’ and employ strategies to enhance infant sleep safety wherever, and whenever it occurs.

## 1 Introduction

New parents make frequent, dynamic decisions regarding their baby’s care and safety in the context of their family’s circumstances; including infant sleep location (1, 2). Preparation and decision-making are influenced by cultural and societal norms and values within communities (1, 3, 4), as well as a family’s economic situation including access to stable accommodation and material basics (5, 6). In most non-Western societies, intentionally sharing sleep on the same sleep surface with a baby is the cultural norm (6–15). In Western, Educated, Industrialised, Rich, Democratic (WEIRD) and predominantly white societies (16), cots and cribs dominate perceptions of ‘ideal’ infant sleep practices, with separate sleep location becoming a valued societal norm during the last 200 years (1, 2, 17, 18).

Despite successes of public health campaigns promoting infant sleep safety in the 1990s, reductions in rates of deaths attributed to Sudden Unexpected Death in Infancy [SUDI; including Sudden Infant Death Syndrome (SIDS) and fatal sleep accidents] have slowed, in some nations plateaued (19–21), while even increased in some countries (22, 23). SUDI remains one of the leading contributors to post-neonatal mortality (24). SUDI which occur in shared sleep environments contribute significantly to total infant mortality each year (25–29). Factors known to increase an infant’s vulnerability (smoke-exposure in pregnancy and postnatally; being born premature or of low birth weight; sharing sleep on a sofa, or with an adult under the influence of drugs or alcohol), increase the risk of SIDS and fatal sleep accident (27, 28, 30–41). Scholarly debate (8, 42–44) continues on how to address shared sleep in infant sleep guidance (27, 28, 30–41).

Public health approaches generally fall into three broad categories: 1. Risk elimination as strict instruction (e.g., ‘do not bedshare’) (45, 46), 2. Risk elimination as preferred practice while framing infant sleep practices as parental choice (e.g., ‘it is not safe, but if you *choose* to, follow these precautions’) (47, 48), and 3. Risk minimisation guidance (e.g., ‘shared sleep is common and happens intentionally and unintentionally’; strategies to reduce risk are provided using neutral language without presenting one option as ‘preferred’ or ‘safest’) (49–53). A rapid review of international documents shows varied language in Western societies to convey these approaches (see Supplementary Table A), supported by a recent evaluation of the consistency of infant safer sleep messaging in Australia by Kruse et al. (54).

Risk elimination, advocated by the American Academy of Pediatrics (AAP), advises against bedsharing under any circumstances (45). This strategy assumes that cribs/cots offer a universal, simple solution for safer infant sleep for all families, with policies focused on ensuring parental compliance with this advice. While the AAP guidelines have influenced safe sleep public health campaigns in many countries, this approach has not significantly reduced SUDI rates which have risen in the US since 2020 (22). Shared sleeping remains a common practice in Western societies for many reasons (8, 21, 42, 55–59).

Shared sleep or bedsharing aligns with human evolutionary design, supporting mothers and infants and prolonging breastfeeding (4, 60–64); strongly suggesting the focus of infant sleep safety should include risk mitigation for shared sleep rather than solely advocating for avoidance. Historically, safe sleep messages have been unidirectional, information giving based on the assumption that a parent’s actions are influenced with information alone (65). Recently, UK and Australian researchers have codesigned safer sleep messages and policy guidelines to improve acceptability and uptake (49, 66, 67).

Building on Salm Ward and Doering’s (68, 69) earlier reviews of mother-infant bedsharing this integrative review examines literature published during the last decade, which centres on shared sleeping using a parent-focused lens. Understanding parent and caregiver experiences with safer sleep advice and information needs is crucial for safer sleep campaigns. By considering diverse family circumstances, this review aims to inform more effective public health messaging and resources. A systematic approach was employed to explore the primary research question: *‘What information do parents want and need to minimise risk if they have an infant under 12 months of age who shares the same sleep surface, intentionally or not?’*

## 2 Methods

An integrative review methodology was chosen to explore the multifaceted phenomenon of shared sleeping because this process supports holistic exploration of complex, health related topics including the flexibility to integrate diverse methodologies (70). Whittemore and Knafl’s five-step integrative review framework (71) was used to guide this review (71): problem identification, literature search, data evaluation, data analysis and presentation.

### 2.1 Search strategy

Four objectives guided research question development using the PICo model (72) (Population, Phenomenon of Interest, Context), to identify these key concepts: 1. Sleep safety challenges, 2. Strategies used, 3. Family experiences with risk factors, and 4. Parental information needs. Literature databases including Scopus, CINAHL, PubMed, PsycNET, and Emcare were searched using relevant keywords for peer reviewed studies published between January 2013 and 13 March 2025. The search strategy was guided by a university librarian. Eligible studies focused on bed-sharing with infants under 12 months of age and reported primary caregiver perceptions or experiences related to reasons for caregiver-infant bed-sharing, associated challenges, and/or solutions and strategies to address these challenges. Studies were included if they were empirical, peer reviewed publications, including systematically conducted literature reviews and publicly available theses published in English between 1 January 2013 and 13 March 2025. Detailed review objectives, inclusion and exclusion criteria, and search strings are contained in Supplementary Table B.

### 2.2 Study selection

**Figure 1:**
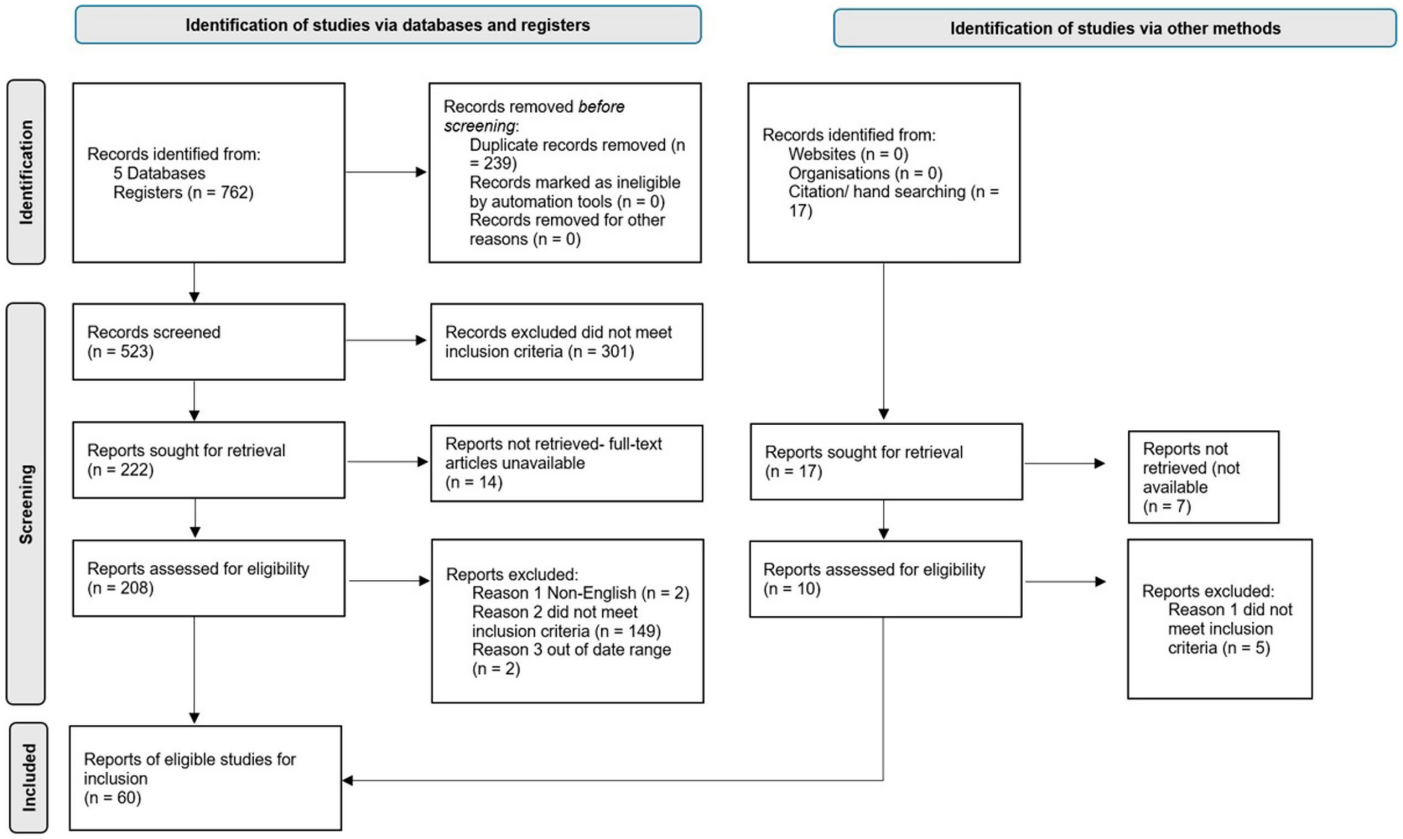
PRISMA flow diagram including screening and inclusion (RRID: SCR_018721).

A systematic search identified 762 studies with 17 additional articles found through a hand search of included reference lists. After duplicate removal (*n* = 239), two researchers (CG, JY) screened titles and abstracts. A third researcher (TD) joined for full text screening and all discrepancies were resolved through discussion and consensus. Ultimately, 60 articles met eligibility criteria. See Figure 1 for PRISMA (RRID: SCR_018721) flowchart.

### 2.3 Quality appraisal

The Quality Assessment for Diverse Studies (QuADS) tool (73) was chosen to undertake methodological and reporting quality of eligible studies to capture the complexity and depth of the topic. No studies were excluded based on this quality assessment. Notably, some studies (*n* = 5, 8%) provide minimal or no detail relating to recruitment data, over a quarter of studies lacked caregiver sampling details appropriate to study aims (*n* = 17, 28%), and many lacked stakeholder involvement in design (*n* = 40, 69%; Supplementary Table C).

### 2.4 Data analysis

**Figure 2:**
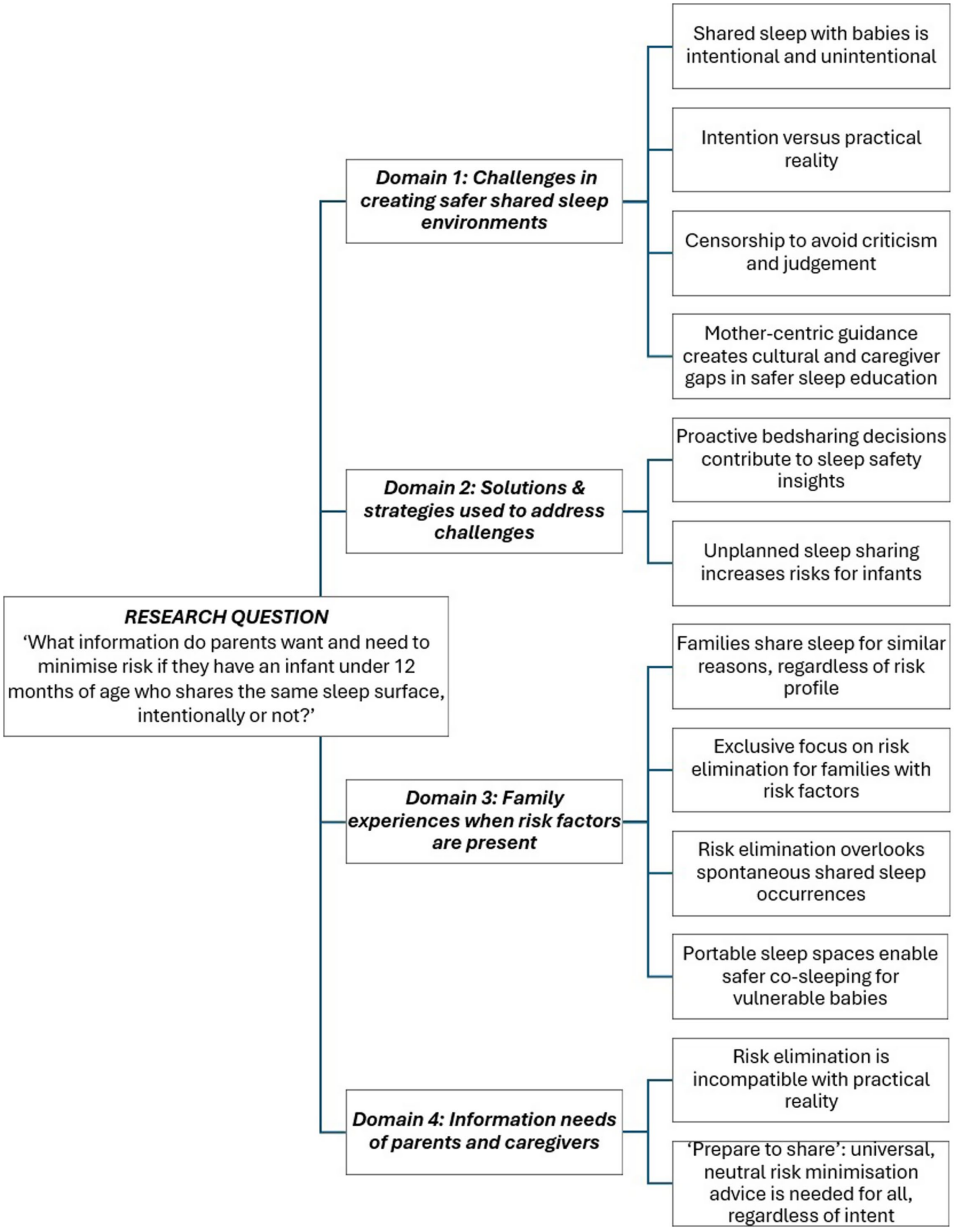
Summary of key domains and themes.

Key data points were extracted and tabulated, including authors, study details, shared sleep approaches and grouped across the four key domains related to the review objectives. See Figure 2. Extracted data covered bedsharing rationale, SUDI risk profiles, challenges, solutions, with information needs differentiated as parent perspectives and/or author conclusions. Data analysis followed Whittemore and Knafl’s framework (*71**),* emphasising data reduction and display. Table 1 contains a summary of data with full extraction details contained in Supplementary Table D.

**Table 1:** Data extraction table.

Article	Sample	Method	Risk profile	Approach	Reasons	Challenges	Solutions	Info needs-parent response/author recommendations^*^
Bailey (108). Australia	Mothers-six breastfeeding mothers who bed-shared with their infants	qualitative interviews	Breastfeeding-protective	Risk minimisation	✓	✓	✓	✓Author
Bailey et al. (109). Australia	Mothers-174 women trained as Australian Breastfeeding Association counsellors	Cross-sectional-one group survey design	Breastfeeding-protective	Risk minimisation	✓			✓Author
Ball et al. (101). UK	Mothers and Fathers-In the LTAS study, 79 expectant mothers and fathers In the BBE study, seventy-seven (77) parents	Feasibility study: Comparative study of perceptions between two cohorts	Smoke exposure Young parental age	Risk minimisation		✓	✓	✓Author
Bamber et al. (102). UK	Infants-477 infant deaths recorded in Project Indigo (2005–86, 2006–84, 2007–89, 2008–77, 2009–76, 2010–65)	Retrospective cohort.	Preterm AND alcohol consumed AND smoke exposure AND social deprivation	Neutral	✓	✓		✓Author
Barrett et al. (96). UK	14 white-British mothers, with 2 fathers and one grandmother joining the mother, recent contact with child protection services in northeast England	Qualitative-In-depth semi-structured interview	Contact with child protection + Preterm/ Alcohol/ / Drugs/ Smoking/ DV/ Social deprivation/ Medically fragile baby No breastfeeding	Neutral-risk minimisation lens	✓	✓	✓	✓Mothers/Author
Barry and McKenna (60). USA	Other-A review	Narrative Review,	Breastfeeding-protective	Risk minimisation	✓	✓	✓	✓Author
Beth Howard et al. (98). USA	Mothers and Fathers-15 English-speaking caregivers of infants. 13 mothers and 2 fathers	Qualitative focus groups	social deprivation	Risk elimination	✓	✓	✓	✓Author
Capper et al. (93). USA	Mothers *n* = 98 Mothers caring for preterm infant at home	Cross-sectional descriptive survey design	Prematurity	Risk elimination	✓	✓	✓	✓Mothers
Caraballo et al. (88). USA	Mothers-43 adolescent mothers	Focus groups	Teen mother/Young Maternal Age	Risk elimination	✓	✓	✓	✓Author
Clarke (84). NZ	Mothers--13 mothers of infants, living in a more socioeconomically deprived suburb in Christchurch NZ	Inductive qualitative design-semi-structured interviews	low socio-economic	Risk minimisation	✓	✓	✓	✓Author
Cole et al. (103). Australia	Mothers (97%)-n3341 caregivers in Australia with young infants	cross-sectional survey	mixed	Risk minimisation	✓	✓	✓	✓Author
Cowan et al. (104). NZ	Mothers (83%) + ‘others’--100 NZ families who received a Portable Sleep Space (PSS) after an earthquake disrupted their sleep conditions	convenience sample, online survey	Disrupted routine, Smoke exposure in pregnancy, prematurity, low birth weight, crowded living, frequent moving	Risk minimisation	✓	✓	✓	✓Author
Crane and Ball (119). UK	Mothers-46 mothers-25 White British origin, 21 Pakistani origin-with infants	In-depth narrative interviews		Risk minimisation	✓	✓		✓Mothers/ Author
Cunningham et al. (130). Australia	Mothers-1126 Australian parents of 8-week-old infants	Cross-sectional survey		Exploratory-leaning toward risk minimisation	✓	✓	✓	✓Author
Doering et al. (120). USA	77% mothers 23% fathers--375 parents-77% mothers, 74% Caucasian	exploratory survey	medically complex infant	Risk minimisation	✓	✓	✓	✓Author
Doering et al. (122). USA	98% mothers 2% fathers--The 49 caregivers represented 10 different countries	pilot study used a mixed-methods, exploratory, descriptive, non-experimental design		Exploratory	✓	✓	✓	✓Author
Ellis (85). UK	Mothers-15 mothers aged between 16–21 years, presented with at least one other factor	qualitative approach using Interpretative Phenomenological Analysis (IPA). Serial in-depth interviews	Teen/Young Maternal Age + smoking; misuse of drugs or alcohol, unemployment or low income; reported housing issues	Exploratory	✓	✓	✓	✓Author
Fangupo et al. (10). NZ	15 caregivers who identified as Ethnically Diverse Pacific Families (EDPF) 9 mothers, 3 fathers, 2 grandmothers	Qualitative interviews	Ethnically Diverse-Pacific families	Exploratory	✓	✓		✓Author
Feld et al. (86). Ecuador.	Women-100 pregnant women	Cross-sectional descriptive design. Self-report surveys		Risk elimination	✓	✓		
Gaertner et al. (105). Germany	Mothers-1400 mothers of infants in Germany	Quantitative interviews and surveys at multiple timepoints	smoke exposure	Risk minimisation	✓	✓	✓	✓Author
Gaydos et al. (76) USA	Mothers and Medical providers-60 African American mothers of young infants, 20 medical providers who serve new mothers	Focus groups (with mothers) and telephone interviews (with providers)	low-income mothers African American	Risk minimisation	✓	✓	✓	✓Author
George et al. (90). NZ	Mothers-14 Māori families-11 interview mother only, 3 interviews with 2 parents	Qualitative interviews	Priority population-Māori	Exploratory/aiming for risk minimisation	✓	✓	✓	✓Author
Gettler et al. (123) USA	Fathers-195 Midwestern US Fathers	Qualitative survey tools	Fathers (non-breastfeeding parent)	Exploratory	✓	✓		
Gilmour et al. (124). Canada	Women-5329 Canadian mothers	Cross-sectional survey	Mixed	Exploratory	✓	✓		
Gustafsson et al. (125). Sweden	Mothers (84.2%) and fathers (15.8%) 76 parents	Qualitative online survey		Exploratory	✓			✓Author
Hamadneh et al. (100). Jordan	Mothers-604 mothers-394 citizens and 210 refugees in Jordan	semi-structured interview completed during a face-to-face interview	Refugee/unstable accommodation AND high smoke exposure and poorly ventilated sleep environments	Risk elimination		✓		
Hauck, et al. (75). USA	96% mother and 4% other-3303 families with financial need for a free crib + at least 1factor, 1729 through to follow-up	pre and post intervention surveys	Priority population-African American, American Indian or Alaska Native, maternal smoking, pre-term or low birth weight, or sibling of a SIDS infant	Risk elimination	✓	✓	✓	
Hauck et al. (74). USA	1,259 mothers who responded to the postpartum survey (mean infant age, 11.2 weeks).	Quantitative -randomized trial-survey	Mixed-Breastfeeding-protective Formula feeding Priority population-African-American	Risk elimination-with a risk minimisation as an add on	✓	✓	✓	✓Author
Herman et al. (77). USA	54 mothers and 13 female supporters and 13 male supporters--73 African-American, or American-Indian caregivers	Focus groups	Priority population-African American, American Indian	Exploratory /leaning toward risk elimination	✓	✓	✓	✓Parents
Hirsch et al. (78). USA	49 fathers/ grandfathers/ uncles/ cousins/ caregivers of infants. 67% African-American, 33% White	Focus Groups	Priority population-African-American, Fathers (non-breastfeeding parent)	Risk elimination	✓	✓	✓	✓Parents
Huber et al. (110). USA	Providers + PRAMS and OPAS data-7 perinatal service providers participating in NAPPSSIIN-2019 PRAMS and OPAS data	Mixed methods-intervention study	mixed	Risk minimisation/risk mitigation		✓	✓	✓HPs
Hutchison et al. (106). NZ	Women-172 mothers of infants	postal survey (quant and qual)		Exploratory	✓	✓		
Hwang et al. (94). USA	23 mothers of preterm infants	in-depth qualitative interviews	Prematurity	Exploratory/leaning towards risk minimisation	✓	✓	✓	✓Author
Hwang et al. (126). USA	3,297 mothers of infants	Postal survey		Exploratory		✓		
Kadakia et al. (80). USA	Mothers-Survey 412 African American parents FG//Interviews-83 African-American parents	cross-sectional mixed methods-survey/focus groups/interviews	Priority Population-African-American social deprivation formula feeding	Risk elimination	✓	✓		✓Author
Lerner et al. (81). USA	63 African American mother-infant dyads	Observational – qualitative via survey and video	Priority population-African-American	Exploratory	✓	✓		✓Author
Louis-Jacques et al. (112). USA	546 physicians and medical students who birthed children from October 2020 through August 2021	Quantitative online survey	Breastfeeding-protective	Exploratory	✓			
Luijk et al. (127). Netherlands	5,095 mothers at 2 months and 5,361 mothers at 24 months a population-based multiethnic (Dutch, Turkish and Moroccan, and Caribbean)	Prospective cohort design-questionnaires and medical records		Neutral	✓	✓	✓	
MacFarlane et al. (91). NZ	Thirty mothers participated in the study	qualitative face to face interviews	Priority population-Māori and Pasifika smoke exposure	Exploratory. Bed-sharing cultural norm and expectation	✓	✓	✓	✓Author
Mathews et al. (79). USA	422 African–American and 90 Hispanic mothers	cross-sectional, multimodal (surveys, qualitative interviews-focus groups or individual	Priority population-African American, CALD, Lower SES, Smoke exposure	Exploratory /risk elimination tone	✓	✓		
McIntosh et al. (92). NZ	240 Māori and Pacifica women-112 intervention group (101 for full intervention), 110 control group	randomised controlled trial	Priority population-Māori and Pacifica, smoke exposure, low birth weight, congenital airways issue, prior SIDS of sibling	Risk elimination w. Some minimisation	✓	✓	✓	✓Author
Moon et al. (113). USA	25 US based mothers	survey and virtual focus groups		Risk elimination	✓	✓	✓	✓Author
Morrison et al. (99). USA	23 mothers with an Opioid Use Disorder (OUD)	Qualitative interviews and thematic analysis	Opioid Use Disorder	Risk elimination	✓	✓	✓	✓Author
Murray et al. (128). Vietnam	21 Vietnamese mothers	Semi-structured qualitative interviews		Exploratory. bed-sharing cultural norm and expectation	✓	✓		✓Author
Osei-Poku et al. (114). Lusaka, Zambia.	478 mothers in Lusaka Zambia	qualitative cross-sectional survey		Risk elimination	✓	✓		
Osei-Poku et al. (121). Lusaka, Zambia.	35 mothers in Lusaka Zambia	Qualitative, focus groups		Risk minimisation/bed-sharing cultural norm and expectation	✓	✓	✓	✓Author
Pease et al. (97). UK	20 mothers from a deprived area of Bristol, UK	Semi structured interviews	Three or more measures of increased risk of SIDS-young maternal age, smoking during pregnancy, three or more children, and a measure of deprivation	Risk minimisation	✓	✓	✓	✓Mothers
Pease et al. (107) UK	Infants-138 SIDS deaths in 2020 compared with 402 SIDS deaths and 1,387 age-equivalent surviving controls	Cohort of SIDS in 2020 compared with a combined analysis of two case-controlled studies	low birth weight, premature, male infants, smoke exposure during pregnancy and after, socioeconomically deprived families, prone, non-sober parent, sleeping on a sofa, times of disrupted routine	Risk minimisation	✓	✓	✓	✓Author
Pretorius et al. (115) USA	526 mothers on Facebook (undescribed locations)	Qualitative data-textual analysis		Risk elimination	✓	✓	✓	✓Mothers
Rudzik and Ball (111). UK	39 mothers in the UK	Qualitative analysis-focus groups	Formula Feeding	Exploratory	✓	✓		
Sahud et al. (65) USA	21 parents (85% mothers) who had practiced non-recommended sleep methods with their infant and had or had not experienced an undesirable sleep event (e.g., fall)	One-on-one phone interviews	Mixed population. Did include priority population-African American	Exploratory-risk minimisation	✓	✓	✓	✓Mothers
Salm Ward et al. (116). USA	615 mothers (pre-and post) and 66 follow-ups	Matched pre and post-test cohort design with follow up survey		Risk elimination	✓	✓	✓	✓Author
Salm Ward (117). USA	Mothers and fathers-22 families (20 mothers and 2 mother–father dyads)	Qualitative semi-structured interviews		Risk elimination w. some minimisation	✓	✓	✓	✓Author
Shimizu et al. (139). Japan	51 Japanese mothers	Qualitative analysis of comments on a parenting forum		Neutral/ Bed-sharing cultural norm and expectation	✓	✓	✓	
Shin et al. (118) USA	411 US women	Pre-post-test surveys-quantitative		Risk elimination		✓	✓	
Stiffler et al. (83). USA	15 African American mothers	Qualitative focus groups	Priority population-African-American	Risk elimination w. Some minimisation	✓	✓	✓	✓Mothers
Tully et al. (95). USA	56 mother infant dyads-26 late preterm and 30 term	Qualitative semi-structured interviews	Premature	Risk minimisation	✓	✓	✓	✓Author
Weil (89). USA	12 young mothers from Cook County, US living in transitional living programs for young mothers	Self-report surveys + Qualitative focus groups	Young Maternal Age	Risk elimination	✓	✓	✓	✓Mothers
Yuma-Guerrero et al. (87). USA	93 pregnant or parenting teenagers 9,507% (*n* = 89) mothers 4.3% fathers (*n* = 4) who are also students in Texas USA	Semi structured focus groups	Teenage mothers	Risk elimination	✓	✓		✓Author
Zoucha et al. (86). USA	84% women and 16% male--19 African-American caregivers	Semi structured qualitative interviews	Priority population-African American	Risk elimination	✓	✓		✓Parents/Caregivers

* This column indicates whether parents directly reported their information needs for safer sleep education/advice or if these information needs were identified by the Author(s) in the study’s discussion or conclusion.

## 3 Results

### 3.1 Eligible study characteristics

Of the 60 eligible studies, the majority were empirical (59/60, 98%) with one narrative literature review. Among empirical studies, 49% (29/59) were qualitative, 33% (*n* = 20/59) mixed methods, and 15% (*n* = 9/59) quantitative studies. Publications were spread across the inclusion period ranging from 1 to 9 publications per year.

### 3.2 Study sample participants

Most studies included women/mothers (54/60, 90%), with 36 (60%) focusing exclusively on women/mothers as participants. Families with increased SUDI risk, frequently considered priority populations for safe sleep messaging, were represented in 55% (*n* = 33) of studies, including: African-American/American-Indian families (11/33, 33%) (65, 74–83), low socio-economic status (*n* = 5/33, 15%) (76, 79, 84–86), adolescent mothers (*n* = 4/33, 12%) (85, 87–89), Māori/ Pasifika families (*n* = 4/33, 12%) (10, 90–92), premature/low-birthweight infants (*n* = 4/33, 12%) (93–96), families experiencing social deprivation (*n* = 4/33, 12%) (80, 96–98), parents with opioid use history (*n* = 2/33, 6%) (96, 99), and refugee and/or transient families (*n* = 1/33, 3%) (100). Smoking exposure was noted in 15 studies (15/60, 25%) (75, 79, 85, 91, 92, 96, 97, 100–107). Breastfeeding, a known protective factor, was a focus in eight (13%) studies (60, 74, 80, 108–112).

### 3.3 Country of origin

Just over half of the studies were from the USA (*n* = 31, 51.6%), with others from Australasia (20%), Europe (18%), Sub-Saharan Africa (3.5%), Asia (3.5%), and single studies from Jordan, Ecuador, and Canada.

### 3.4 Approaches used for shared sleep safety

Shared sleep philosophies underpinning the approach to safer sleep messaging and assumptions in published studies were grouped into five categories: (a) risk elimination, viewing all shared sleep as hazardous (*n* = 20) (74, 75, 78, 80, 82, 83, 86–89, 93, 98–100, 113–118); (b) risk minimisation, acknowledging its occurrence and focusing on reducing risks (*n* = 17) (60, 76, 84, 92, 95, 97, 101, 103–105, 107–110, 119–121); (c) exploratory, describing the phenomenon without a specific aim of risk reduction or elimination (*n* = 16) (10, 81, 85, 91, 102, 106, 111, 112, 122–129); (d) exploratory with a risk minimisation aim (*n* = 5) (65, 90, 94, 96, 130); and (e) exploratory with a risk elimination aim (*n* = 2) (77, 79). See Table 1.

### 3.5 Analysis of results

This analysis systematically addresses the four study objectives. Twelve themes generated from the data were grouped under four domains. Themes will be identified, compared and discussed to address the related objectives. Figure 2 summarises key domains and themes.

#### 3.5.1 Domain 1: challenges in creating safer shared sleep environments

Four themes were generated regarding the challenges parents faced in creating safer shared sleep environments*: Shared sleep with babies is intentional and unintentional; Intention versus practical reality; Censorship to avoid criticism and judgement; and Mother-centric guidance creates cultural and caregiver gaps in safer sleep education.* Despite being advised against co-sleeping (10, 65, 75–79, 83, 88, 90, 93, 95, 96, 98, 106, 113, 116–118), many parents engaged in both intentional and unintentional shared sleep due to the practical challenges and emotional demands of infant care, often without guidance or support. In more than half of the included studies (*n* = 32, 53%), parent reports consistently suggested that they were unprepared for the reality of infant sleep and related care both day and night (frequent waking, feeding, comfort and settling through co-regulation), and this often led to reactive and/or unintentional (spontaneous) shared sleep (10, 65, 74, 77, 78, 81, 83–85, 87–89, 91, 93–95, 97–99, 103, 106, 111, 113, 115–120, 123, 124, 127, 130). The factors associated with unintentionally falling asleep with a baby included infant-related factors such as night-time feeding requirements (65, 74, 81, 85, 87, 95, 97, 103, 106, 116, 117, 119), infant temperament/preferences (10, 65, 77, 78, 84, 88, 93, 96, 116, 123, 127), infant wakefulness (113, 123, 127), and the need for extra comfort and soothing when babies were unsettled, sick or experiencing discomfort (81, 83, 89, 91, 95, 96, 98, 99, 106, 113, 115, 120, 124). Adult-related factors included the experience of overwhelming exhaustion and fatigue because of the intensity of infant care around sleep (65, 74, 77, 78, 84, 85, 91, 94, 96–99, 103, 111, 113, 116, 117, 120, 130). and maternal anxiety (65, 83, 96).

Unintentional and some forms of reactive sleep (in response to infant need or circumstance) (10, 77, 78, 81, 83–85, 87–89, 91, 93–99, 103, 106, 111, 113, 115–120, 123, 124, 127, 130) often occurred in locations or environments that increase the risk of sleep accidents and SUDI (including SIDS), particularly if no pre-planning was involved (65, 74, 76, 85, 91, 97, 102, 107, 113, 117, 119, 120, 130). The reality of infant care created dynamic challenges for parents as they negotiated meeting their infant’s needs in ways that also met their own physiological need for rest and sleep in the context of their family’s life. The perceived comfort of their infant (or alleviating the experience of discomfort) provided by sharing sleep was a clear priority for parents (10, 60, 65, 77–79, 81–85, 87–89, 91, 95, 98, 99, 103, 108, 112, 113, 116, 123, 124, 127, 130) alongside providing for infant safety. Most parents in the reported studies (*n* = 34/60) initially planned, and had prepared, a surface (e.g., bassinet/cot) to sleep their baby separately, predominantly due to information provided by health professionals, but reported they now shared sleep some of the time and in some instances, for all sleep due to a variety of reasons (10, 65, 77, 78, 81, 83–85, 87–89, 91, 93–99, 103, 105, 106, 111, 113, 115–120, 123, 124, 127, 130).

Not all parents felt they could openly discuss or even disclose shared sleep with health professionals as they knew it went against recommendations (65, 83–85, 89, 90, 96, 99, 108). Interestingly, 52% of breastfeeding physicians who reported bedsharing in a study by Louis-Jacques et al. (112) did not disclose this practice to their child’s physician (despite being medical peers). A limited number of studies (*n* = 6) described shared sleeping occurring due to lack of access to a cot/crib/bassinet, including the ability to procure one (75, 79, 92, 95, 117, 121). Parents reported that many health professionals did not appear to be forthcoming with risk minimisation strategies (65, 76, 110). In the absence of formal guidance, parents self-generated strategies and solutions to address their safety fears/concerns and minimise risk for their infant (See Table 2). The most common parental fears were of potential smothering or suffocation (65, 83, 91, 95–97, 113, 115, 121, 122), baby rolling off the bed or other sleep surface (couch/sofa) (65, 76, 78, 88, 89, 94, 99, 103, 108, 113, 116, 121, 130), co-sleeping itself (i.e., bedsharing) so sleeping on a sofa to avoid bedsharing (65, 74, 76, 116, 119, 120, 130), the fear of choking or aspiration (77, 79, 96, 121) and fragility of their baby which drove a parental compulsion--often described as a need--to share sleep in order to monitor baby closely. This was further evident in reports by parents of premature or medically complex babies (93, 94, 96, 122), and babies experiencing withdrawals from opioids (96, 99). Table 2 provides a summary of the solutions which parents employed to address challenges stemming from parent fears.

**Table 2:** Parent-generated solutions to address challenges arising from parental fears.

Parental fear	Self-generated solutions that may inadvertently increase risk
Smothering/suffocation	Use of a three-sided, bedside sleeper/cot to allow proximal shared sleep in own space but incorrectly installed with a gap left, increasing a risk of entrapment (85) Propping baby on pillow or placing pillow between baby and adult to address fear of rolling on baby, increasing risk of suffocation (91, 96) Positioning infant above adult shoulders to address fear of suffocation under blankets or overlay (116) Propping infant on mother’s arm to reduce fear of rolling on baby (116) (may increase risk of airway obstruction through chin to chest positioning) Stuffing blankets into cracks of sofa to try to reduce risk of entrapment while sofa-sharing (113) (creates soft surfaces) Baby sleeping between father’s arms as a cradle to prevent smothering (78)
Baby rolling/falling	Use of pillows/blankets around baby to prevent rolling or on floor to cushion fall increasing risk of suffocation (65, 76, 88, 99, 103, 113, 116) Moved bed against wall to prevent falls but increasing risk of entrapment through wedging (65, 78, 89, 94, 121, 130) Use of a three-sided bedside sleeper to provide a barrier to prevent baby rolling off bed (116) Use of bedrails to prevent baby rolling off bed increasing risk of entrapment (108, 116) Positioning infant perpendicular in adult bed to try to prevent baby from rolling off (116) Positioning infant between adults to block from falling odd side of bed (65) Sleeping on couch as couch was lower than bed to reduce height of any potential fall (65)
Co-sleeping	Sleeping on chairs or sofas to avoid ‘co-sleeping’ (65, 74, 76, 116, 119, 120, 130)
Choking/aspiration	Sleeping baby prone or on their side to prevent choking or aspiration (77, 79, 121)
Fragility	Sleeping infant on chest to monitor breathing (116) Preterm baby propped on pillow after feeds (95)
Comfort	Use of pillows or blankets to soften a hard surface (85) Sleeping on a softer adult mattress or lounge (113, 114, 116)

While mothers were participants in 90% (*n* = 54) of eligible studies and were exclusive participants in 60% (*n* = 36), it was clear that for many of the families, other caregivers (usually fathers and grandmothers) provided direct care for the infant (10, 77–79, 84, 85, 88, 91, 97, 107, 127). Fathers and other caregivers sharing sleep with infants were reported in 17 studies (10, 77–80, 84, 87, 90, 98, 116, 119–121, 123, 128–130). Limited guidance for the broader caregiving circle created challenges for mothers, who had to share and ‘enforce’ safe sleep advice while managing differing opinions (83, 87). This led to conflicts in some families (83, 87, 94), especially as other caregivers, often responsible for daytime infant care, had varying practices (78, 94). Studies indicated that sleep safety was often less prioritised during daytime and times of changed routine (78, 84, 97, 99, 103, 113, 120).

Nine studies utilised the term ‘parent’, ‘parenting’ and/or ‘parental’ in framing discussion of research methods, results and findings but the data were unclear if caregivers other than the mother were present in the infant’s sleep environment (75, 88, 93, 95, 110, 113, 115, 122, 125). Analysis of infant deaths was fraught with similar concerns. For example, the term ‘adults’ was used by Pease et al. (107) in a comparative analysis of infant deaths (*n* = 540) occurring between 1993 and 2020. Results from a retrospective cohort study (*n* = 477 infant deaths) by Bamber et al. (102) indicated that some of these deaths occurred in the presence of more than one adult, without any description of the adult’s relationship with the infant. In contrast, Weil’s (89) bivariate analysis of sleep related infant deaths in a Illinois dataset, identified the presence of fathers and ‘other’ persons at the times of death.

Breastfeeding was encouraged in cohorts with and without an increased risk of SUDI due to the many benefits breastfeeding offers for both mothers and their babies (75, 76, 79–81, 90–92, 94, 110, 112). Breastfeeding and co-sleeping practices are closely related and mutually supportive. Parents found the advice to avoid co-sleeping challenging as it seemingly contradicted the practical implications of successful breastfeeding (90, 108).

#### 3.5.2 Domain 2: solutions and strategies to address challenges

Two themes were identified related to solutions and strategies: *Proactive bedsharing decisions contribute to sleep safety insights*; and *Unplanned sleep sharing increases risks for infants*. Results indicated that parents who proactively planned to bedshare with their infant actively attempted to minimise risk in their shared sleep environment and sometimes sought access to resources and information to guide their attempts (65, 76–79, 84, 88–92, 94–97, 99, 101, 103, 108, 113, 115, 116, 121, 129, 130). Some solutions generated by parents were in alignment with contemporary risk minimisation approaches (49) for example keeping loose bedding/pillows away from baby (65, 76, 84, 113, 116), use of a firm, flat mattress (108, 120, 122) and not smoking if bed-sharing or during pregnancy (76, 84, 91, 113, 122). Other actions may have inadvertently increased risk (49) such as sleeping on chairs/sofas to avoid bed-sharing (65, 74, 76, 116, 119, 120, 130) or placing blankets/pillows around baby or on floor for protection in an attempt to prevent or cushion a potential fall (65, 76, 88, 99, 103, 113, 116) (please see Table 3 for a summary of the alignment of parent-generated solutions to address safer shared sleeping challenges with current risk minimisation strategies).

**Table 3:** Alignment of parent-generated solutions to address safer shared sleeping challenges with current risk minimisation strategies.

Suggestions that align with current risk minimisation strategies	Understanding normal infant sleep (including frequent arousals which are protective) and breastfeeding (also protective) (60, 108) helps parents to be proactive to avoid falling asleep in potentially unsafe environments For families with additional risk factors-using a baby box/portable sleeping space/Wahakura/Pēpi-pod/bassinet to avoid direct bed-sharing (e.g., smokers/prem babies/low birth weight/emergent (earthquake) setting) (90–92, 101, 104) Use of a three-sided cot to keep baby close but facilitate own sleep space or as a barrier to prevent baby from falling off edge of bed; this was also described as a challenge with not all parents being aware of or understanding the importance of correct setup to avoid entrapment risks (85, 105, 116). Baby had own space on bed (large enough mattress to have a clear space around baby), own blanket/sleep sack (78, 84, 91, 113, 122) Not smoking if bed-sharing or during pregnancy (76, 90, 105, 121) Perceived increased maternal awareness or vigilance (lighter sleep, ‘mum’ sleep, which is supported by findings in Mosko et al. (154) that found a high level of synchronicity between mother and infant arousals while bed-sharing (65, 91, 96, 97, 116, 121) Consciously purchased and/or use of a firm, flat mattress (108, 120, 122) Use of the protective C-position by the mother around baby (78, 94, 116) Baby always on their back (supine position) (76, 113) Loose adult blankets/pillows keep away from baby (65, 76, 84, 113, 116) Baby has a separate but adjoining futon spread above the mother’s head (129) Sober parent (76, 116) Trundle beds, extra single mattresses to sleep older siblings separately from baby (76, 108) Baby never left alone on an adult bed (76, 119, 121) Partner slept in different room (84) Mattress lowered onto the floor to reduce risk of falling (130)
Suggestions that do not align with current risk minimisation strategies	Sleeping/feeding on chairs or sofas to avoid bed-sharing (65, 74, 76, 116, 119, 120, 130) Sleeping infant on chest to monitor breathing (116) Infant positioned above the adult’s shoulders (116) Propping baby on mother’s arm to avoid perceived risk of choking or of mother rolling on infant (116) Moved bed against wall and/or put baby between adults to reduce risk of falling (65, 78, 94, 121, 130) Infant placed perpendicular to the mother to prevent infant from rolling out of bed (116) Makeshift beds once baby outgrows Moses Basket (85) Use of pillows and blankets to soften a hard surface to improve infant comfort (85, 91) Use of bedrails to prevent falls (108, 116) Actions for avoiding falling asleep accidentally such as setting a 20-min timer or arranging help for infant caregiving to promote parental sleep, turning on a light during feeds, walking around a bit (74, 85, 91, 120) Blankets/Pillows propping baby on/around baby for protection/on floor in case of fall (65, 76, 88, 99, 103, 113, 116) Bed-sharing to closely monitor a baby who only sleeps prone (79)

Safety concerns prompted these parental solutions related to suffocation, baby rolling or falling, co-sleeping itself, choking/aspiration, the infant’s perceived fragility and infant’s comfort (alleviating perceived discomfort; see Table 2). Some parents, particularly those participating in studies based in North America, accessed alternative guidance (to the AAP) such as La Leche League International’s Safe Sleep 7 guidance to inform their risk minimisation actions (94, 113).

The presence of siblings and older children in the household was reported in 39% (*n* = 22) of the eligible literature; and in some instances, were described as sharing the bed with the mother and infant (117, 128, 129). Some studies described actions parents took to facilitate a separate sleep space for their older child/ren, to separate from the new baby (e.g., trundle beds) (76, 108).

#### 3.5.3 Domain 3: family experience when risk factors are present

Four themes were generated regarding the experience of families when risk factors were present*: Families share sleep for similar reasons regardless of risk profile; Exclusive focus on risk elimination for families with risk factors; Risk elimination overlooks spontaneous shared sleep occurrences; Portable sleep spaces enable safer co-sleeping for vulnerable babies*. There was broad agreement (83%, *n* = 50/60) across the literature that certain factors are associated with a higher risk of SUDI, particularly in the shared sleep environment. These circumstances include an infant who is smoke-exposed, premature, low birthweight, non-sober caregiver (alcohol or drug-effected), young maternal age, low socioeconomic status, infant not breastfed or a member of a priority population (population groups who have been identified as having higher rates of SUDI than the general population). In these circumstances, it appeared to be the norm for any form of shared sleep to be advised against regardless of whether the research study was utilising a risk elimination, exploratory, or risk minimisation approach to guide recommendations for practice (10, 60, 75–80, 82–95, 97–111, 113–120, 122, 124, 128, 130). A distinct exception was Barrett et al. (96) who recommended practitioners discuss safer ways of co-sleeping as part of the safer sleep planning for these particular families. Parents and families who met criteria for one or more of these associated risk factors frequently expressed awareness of the advice not to co-sleep with their infant (10, 75, 76, 79, 80, 84, 87, 90–94, 96–99). However, results of this review strongly indicated that these families are sharing sleep for similar reasons as their lower-risk peers (Table 4). Most parents (majority mothers) from these priority populations planned to sleep their baby separately but reported they now shared sleep intermittently or regularly (10, 77, 78, 83, 87–89, 91, 93–99, 120). Parents felt unable to discuss their practices with health professionals due to fear of judgment, stigma, or punitive action, including child safety referrals, consistent with their ‘low risk’ counterparts (84, 89, 96, 99). Parents reported that few health professionals were forthcoming with risk minimisation strategies (76, 110).

**Table 4:** Top 5 reasons for bed-sharing comparing family risk profiles.

Reason for bed-sharing (*n* = 145)	Families with associated risk factors/priority population	Mix of families with and without risks	Families without associated risk factors (general population samples)
1. Breastfeeding (*n* = 37/145, 26%)	(*n* = 17/37, 46%) (77, 78, 80, 81, 83, 84, 87–92, 94, 95, 98, 102, 106)	(*n* = 11/37, 30%) (74, 86, 103, 105, 111, 116, 119, 120, 124, 127, 130)	(*n* = 9/37, 24%) (108, 109, 106, 112, 121, 115, 117, 129
2. Comforting for infants (soothing, settling) Comforting for mother/parent–parental preference/ enjoyment/satisfaction/pride/comfort in bed-sharing (*n* = 34/145, 23%)	(*n* = 17/34, 50%) (10, 77, 78, 81, 82, 84, 85, 87, 89, 91, 93–96, 98, 99, 102)	(*n* = 7/34, 21%) (65, 74, 86, 103, 120, 122, 130)	(*n* = 10/34, 29%) (108, 60, 123, 125, 112, 113, 121, 115, 117, 169)
3. Monitoring/safety/protection (*n* = 29/145, 20%)	(*n* = 19/29, 66%) (76–79, 82, 83, 87–91, 93–98, 104, 119)	(*n* = 3/29, 10%) (65, 86, 120)	(*n* = 7/29, 24%) (60, 108, 112, 115, 117, 121, 129)
4. Better/more sleep (for mother and/or baby) (*n* = 24/145, 17%)	(*n* = 13/24, 54%) (76–78, 88–91, 93–96, 98, 99)	(*n* = 5/24, 21%) (65, 111, 122, 124, 130)	(*n* = 6/24, 25%) (60, 108, 112, 113, 121, 125)
5. Exhaustion/Fatigue (*n* = 21/145, 14%)	(*n* = 14/21, 67%) (77, 78, 83–85, 88–91, 94–97, 99)	(*n* = 6/21, 28%) (65, 103, 116, 120, 124, 130)	(*n* = 1/21, 5%) (113)

In contrast, evaluations of several, novel in-bed portable sleep space (PSS) programs (NZ Pēpi-Pod® Program (91, 92, 104) and wahakura programs (91)) described valuable culturally-appropriate tools that supported parents to make shared sleep safer in the first few months of life (41, 131, 132). McIntosh et al. (91) conducted a randomised controlled trial in New Zealand with 211 women who met eligibility criteria including maternal smoking, second-hand smoke exposure, low birthweight, airway issues, or a family history of SUDI. The Pēpi-Pod® (in-bed infant sleep space designed for 0–4 months) was widely accepted and used by nearly half of participants at 2 months. However, bedsharing remained high (61% at 2 months, 81% at 4 months, when most infants had outgrown the pod). The intervention also appeared to support breastfeeding, likely due to close maternal–infant contact. Similarly, in an evaluation by Cowan et al. (104), 13% of parents were direct bedsharing after discontinuing use of the Pēpi-Pod® when their baby had outgrown the device. Importantly, the primary purpose of Pēpi-Pod Program® (sleep space dimensions: 72cmL x 40cmW x 15.5 cm) and wahakura programs is not to eliminate bedsharing, but rather to support close, proximate care while protecting vulnerable, smoke-exposed infants during a developmentally vulnerable period (0–14 weeks) from suffocation (133). Infant airway protection strategies are key features of Pēpi-Pod Program® educational materials (133, 134). Notably, results indicate a continued occurrence of direct bedsharing before, during and after implementation. Similarly, Hauck et al. (75) reported that 16% of participants in the U. S. National Crib Distribution Program continued bedsharing, with no report of risk reduction strategies provided. Ball et al. (101) evaluated two infant sleep spaces: a shallow, transparent propylene box (72.5cmL x 33.5cmW x 18cmH) with safe sleep information (written and video), and a higher-sided (65cmL x 40cmW x 28cmH), opaque cardboard box with access to on-line education. Parents preferred the lower-sided propylene option, describing easier visual and physical access to their baby in addition to hygiene and portability benefits (101). Salm Ward et al. (117) also reported that 28.8% of 66 respondents sometimes fell asleep with their infants on sofas, chairs, or in bed while feeding during follow-up of a safe sleep and crib distribution program.

#### 3.5.4 Domain 4: information needs of parents and caregivers

Two themes related to the information needs of parents and caregivers were identified*: Risk elimination is incompatible with practical reality; ‘Prepare to share’: universal, neutral risk minimisation advice is needed for all, regardless of intent*. Few studies (*n* = 4/60) collected and described parents’ information needs on shared sleep (4/60) (77, 89, 94, 113) or safe sleep more broadly (*n* = 6/60) (65, 78, 93, 96, 97, 115). Only 11% (7/60) explored preferred delivery of existing messages (65, 78, 82, 83, 89, 91, 97) while 56% (34/60) offered author-led recommendations for future safe sleep information provision to parents (See Supplementary Table D for individual study detail). Notably, 35% (21/60) called for more guidance to improve shared sleep safety (60, 65, 74, 76, 84, 85, 92, 95–97, 101, 103–105, 107–110, 119–121).

Of the 10 studies addressing parental perspectives on their information needs for shared or safer sleep (65, 77, 78, 89, 93, 94, 96, 97, 113, 115), findings showed a clear desire for practical risk minimisation strategies to support sleep, shared sleep and infant sleep positioning. Herman et al. (77), noted that current safe infant sleep recommendations often fail to meet real-world needs; a view echoed in 46% (28/60) of studies in which parents found that the advice not to co-sleep was not always achievable in practice (10, 65, 75–81, 83, 84, 91, 93, 95–99, 103, 105, 106, 115–117, 120, 123, 124, 130). Parents in other studies described safe sleep advice as ‘unrealistic’ (65, 113), ‘not feasible’ (115), ‘condescending’ (97), ‘ridiculous’ (87), and ‘rigid’ (89) and ‘not incorporating the needs of the child’ (89). Some reported that advice not to co-sleep went against their instinct (88, 90, 97) or was not applicable to them (85, 108, 111, 119, 127, 130). Pakistani mothers in a study by Crane and Ball (119) repeatedly expressed that the safe sleep guidance was not written for them, but for their white counterparts. These findings highlight the need for culturally sensitive, relevant and inclusive safer sleep advice (119, 127).

Parents requested targeted safer sleep education to include the broader infant caregiving circle to help combat the conflicting advice and care practices parents face, when information provision is mother-centric (10, 77–79, 84, 85, 88, 91, 94, 97, 107, 127). Social supports (in-community and online) were identified as important, timely sources of information to mothers as they navigate the complexity of infant sleep, their child’s dynamic development, and their family’s life (89, 96, 115). Results indicate there is a wide variation in the quality of the safer sleep advice provided within these, often unmoderated, spaces (115).

Results suggest that mothers are motivated and feel confident in minimising suffocation risks (89), however parents often believe ‘SIDS’ is largely an unavoidable phenomenon, and feel they can do little to ‘prevent’ it (78, 79, 83). Understanding the rationale underpinning safer sleep recommendations was a priority for mothers in two UK-based studies (96, 97). These mothers expressed the desire for health professionals to take the time to explain why they advised certain practices and to allow parents time to absorb the information and ask questions. Participants expressed the need for individualised and collaborative conversations with trusted others (96, 97). Fathers too, wanted to be treated as competent, responsible infant caregivers and for messaging to acknowledge their active involvement (78).

## 4 Discussion

This review explored the research question: ‘*What information do parents want and need to minimise risk if they have an infant under 12 months of age who shares the same sleep surface, intentionally or not?’*. The challenges parents and caregivers face in navigating safer sleep messaging, the strategies parents employed in making shared sleep safer for their baby, parental experiences with higher risk infants, and the information they seek, were identified. This discussion provides a synthesis of these findings, comparing and contrasting themes generated from this review with those originally identified by Salm Ward (69) over a decade ago. Education, practice and policy implications for contemporary health professionals and families will also be presented.

The literature highlights several challenges parents face in creating safer shared sleep environments. Many parents share sleep, intentionally and unintentionally, due to factors such as infant needs (e.g., nighttime feeding, comfort) and exhaustion, despite being aware of associated risks in some circumstances. Acknowledging parental fatigue as a separate driver for shared sleep is an important addition to Salm Ward’s (69) earlier findings. This finding highlights the common and consistent occurrence of unintentional shared sleep due to parent exhaustion (65, 74, 77, 78, 84, 85, 91, 94, 96–99, 103, 111, 113, 116, 117, 120, 130), a phenomenon not adequately addressed by safe sleep approaches that assume shared sleep is always a conscious choice (68, 69).

Human sleep physiology dictates that we will sleep (135) and in the postpartum context, breastfeeding-induced hormones also promote sleep (13, 74, 136, 137). Given our human biology, it seems appropriate that sleep safety policies should educate parents on the likelihood of falling asleep with their baby, regardless of intention, and provide strategies for how to prepare the environment to make it safer if it occurs (60, 65, 74–76, 85, 93, 98, 106, 117, 119, 120, 122). Simply having a separate sleep space, and an intention not to share, is likely to be insufficient (65, 74). Providing universal, neutral guidance on how to minimise risks when sharing sleep can help prevent sleep-related accidents, including among ‘accidental bedsharers’. Preparing families with this information is not a promotion of bedsharing, nor an endorsement. Rather, this *prepare to share* approach recognises that many parents do- and will-bedshare, and ensures they have access to evidence-based safety information regardless of intent or circumstance.

This review highlighted a gap in the current literature, demonstrating that most education on sleep safety focusses on mothers, often overlooking the roles of other caregivers. This reflects an outdated assumption of a nuclear family model, excluding the important role of fathers and multigenerational caregiving, which could be leveraged in future infant sleep safety approaches. Earlier recommendations by Pease et al. support the need for safer sleep messaging to include all caregivers (138).

Although it is well documented that parents and caregivers often share sleep with their infants, research frequently lacks clarity about who exactly is involved (75, 82, 87, 89, 90, 92, 98, 101, 103, 119, 122, 125). Ambiguous and inconsistent language, especially in studies reporting infant deaths (102, 107), makes it difficult to determine the identity, relationship, and caregiving role (active or passive) of those sharing the sleep environment. These details are vital for accurately assessing risk and tailoring education and support to specific family circumstances. While previous research has highlighted the need to consider partners in bedsharing (13), and this review underscores the involvement of a broader range of caregivers beyond parents.

This review supports earlier findings (40, 139) that infant sleep safety is often deprioritised during daytime naps, routine disruptions (e.g., illness, travel) and emergencies (78, 97, 99, 103, 113). Future risk minimisation guidelines should address these contexts specifically (49), such as the Australian Breastfeeding Association’s Supporting Safer Sleep for Babies in Evacuation Centres (140). Parents and carers desire guidance in safely adapting to novel environments while continuing to act responsively to their baby’s needs.

Extensive accounts in the contemporary literature highlighted an earlier finding from Rowe (141): parental intentions to balance family sleep needs while maximising infant safety (10, 60, 65, 77–79, 81–85, 87–89, 91, 95, 96, 98, 99, 103, 108, 113, 116, 123, 124, 127, 130). When following standard safe sleep advice (i.e., risk elimination or risk elimination as preferred practice) was not feasible, parents created their own solutions especially to prevent infant rolling or falls (from the bed/sleep space), often without formal guidance.

A concerning finding was that many parents fear judgement and feel unsafe disclosing bedsharing with healthcare providers, leading to underreporting (65, 83–85, 89, 90, 96, 99, 109). Salm Ward and Doering’s (68) review also highlighted stigma as a key factor contributing to underreporting. These results demonstrate that parents and carers have a clearly expressed desire, and need, for health professionals to engage in open, non-judgemental conversations regarding bedsharing and to provide anticipatory guidance without negative rhetoric (65, 96, 142). Proactive guidance from health professionals is the safest and most appropriate option, as merely providing risk minimisation guidance after shared sleep disclosure is insufficient. This approach overlooks parents who either do not disclose or unintentionally share sleep for many and diverse reasons, as highlighted in this review (65, 96). Parents who reported bedsharing offered valuable insights into safer sleep strategies. Their contributions highlighted practical gaps and the utility of risk minimisation approaches. Incorporating parental expertise and experiences could improve safer sleep messaging, as supported by Pease et al. (138).

Intention to bedshare appeared to be associated with better preparation and uptake of practices which protect an infant’s airway. In line with findings from the Salm Ward review (69), parents who plan to bedshare were more likely to take steps to minimise risks, while unplanned shared sleep (60, 75, 76, 85, 93, 98, 106, 117, 119, 120, 122), and in particular, sofa sharing (29, 74, 76, 85, 91, 97, 102, 107, 113, 117, 119, 120, 130), may increase the likelihood of fatal sleeping accidents and SUDI. These findings, again, highlight the imperative for accessible risk minimisation resources as part of universal safer sleep guidance, regardless of a parent’s sleep location plan (65). An important finding from this review is that families with factors increasing SUDI risk, share sleep with their infants for similar reasons as lower-risk counterparts. This was also evident in Salm Ward’s earlier review (69). This raises concern about the blanket advice to avoid co-sleeping, which fails to consider the dynamic and complex interplay of infant and adult sleep needs. Factors such as feeding (breast, bottle, mixed), settling, soothing and adult sleep environment all contribute to shared sleep plans and actions, which are not always intentional. Unintentional, spontaneous shared sleep exists beyond active, parental choice, regardless of risk profile. Ignoring non-volitional aspects of sleep may worsen outcomes for marginalised families, deepen disparities in infant sleep safety, and fail to address the universal challenge of managing sleep needs of both infant and their families (4).

Results from this review indicate the need to address socio-economic conditions (e.g., poverty, housing, food and job insecurity) (103, 143) that drive factors increasing the risk of SUDI. Smoking, substance use and lower breastfeeding rates (4, 37, 136, 144, 145) are all associated with social deprivation. For families and their babies to thrive, efforts to address these factors require sustained, meaningful effort to reduce systemic factors which fuel disparities in communities (5, 6, 143, 144, 146).

The use of portable, in-bed sleeping devices (e.g., Pēpi-Pod® Program or wahakura) for vulnerable infants (e.g., smoke-exposed, LBW, premature) within culturally appropriate education programs offers a safer sleep intervention that aligns with families’ preferences to keep their baby close in bed. This approach is supported by New Zealand and Australian findings, and emphasise the importance of trust, culturally competent delivery, and parent involvement in successful health promotion, which has been associated with infant mortality reductions in both countries (28, 134). However a recent study evaluation has also highlighted how monitoring for program fidelity is essential to ensure such programs are delivered as intended and reach the target population (147). While these in-bed sleep devices provide safer sleep options for vulnerable babies, they do not eliminate the need for ongoing risk minimisation guidance, especially once babies grow out of the spaces (usually by 4–5 months) as bed-sharing remains common in the early years of childhood. Recent Australian clinical guidelines, codesigned and based on risk minimisation (49) have emphasised this importance of considering the interaction of sleep environments and the dynamic growth and development of infants, particularly during their first year.

Parents in this review clearly expressed that the current safe sleep messaging approaches, based on risk elimination, are insufficient to meet their needs, particularly for non-white, non-Western cultures, where bed-sharing is a common practice. Many parents suggested or clearly stated that the current advice and messages were not applicable or practicable to them (10, 65, 75–81, 83, 84, 91, 93, 95–99, 103, 105, 106, 115–117, 119, 120, 123, 124, 130). These findings are supported by Volpe and Ball (148) who identified ‘trade-offs’ between aligning with or against safe sleep guidance was a reality for most mothers. Interestingly, despite parents reporting that risk elimination advice and actual infant care were incompatible, many studies continued to reinforce, in their conclusions, the importance of risk elimination messaging, sometimes contradicting or failing to respond to the evidence they presented (75, 77–80, 93, 98, 99, 113, 115, 117).

Parents were open to, and desired opportunities, to learn ways to improve safety while bed-sharing with their infants. This supports findings identified in the Salm Ward review that messaging on safer bed-sharing was needed (69). There was a distinct and expressed need for advice to encompass all infant caregivers, not only mothers. Online social support was valued for providing timely answers, support and solidarity as they navigated their infant’s needs and evolving family circumstances (89, 115).

Caregivers (mostly mothers) expressed a need for non-judgemental support from health professionals to improve shared sleep safety through conversations, and specifically time to process and ask questions about the rationale underpinning safer sleep guidance (96, 97). Understanding the ‘why’ or the rationale underpinning messages emerged as a key finding to inform future research and public health campaigns. Developing improved messaging including safer sleep conversations that incorporate the reasons for advice may help parents better apply these understandings when responding in novel sleep situations (96, 97).

### 4.1 Research opportunities

This review identified several research opportunities, particularly the need for studies that investigate best practice approaches to incorporate identified parent information needs, including risk minimisation strategies, into parent-facing resources with parents and caregivers involved as key stakeholders. Parents have articulated several fears and safety concerns relevant to current infant sleep messaging highlighting the importance of participatory research approaches supporting the co-development and co-design of safer infant sleep messages, campaigns and specific interventions with parents as active contributors (149).

Building on findings by Shiells et al. (150), there is potential to improve the impact of safer sleep information by using evidence-based behaviour change models, such as COM-B, to focusing on factors influencing human actions, including capabilities, opportunities and motivations. The Baby Sleep Planner (66), developed through co-design with parents and practitioners in the UK, offers an interesting and potentially promising framework for developing and evaluating context specific resources in different locations (151).

Parent fatigue was a distinct and relatively unexplored driver of unintentional shared sleep. Future research should investigate the contextual factors and environments that contribute to unintentional shared sleep among fatigued parents, evaluate how safer sleep interventions can be adapted to acknowledge and address caregiver exhaustion without resulting in increased caregiver-infant separation, early breastfeeding cessation or reduction in parental responsiveness (18, 152, 153), and explore the influence of support systems in reducing fatigue-related sleep risks, including the role of other family members.

Shared sleep safety for broader caregiving circles including other family members, and the influence of shared sleeping with older infants, toddlers and children is also a priority for further exploration. Review findings also highlighted a paucity of studies examining shared sleep safety from the perspectives of parents and caregivers with multiple infants, disabilities (physical, cognitive, psychosocial), and from Australian Aboriginal and Torres Strait Islander families, culturally and linguistically diverse families, LGBTIQA+ families, and foster carers.

### 4.2 Strengths and limitations

Infant sleep safety is a public health priority, and the integrative review method provided a robust framework to critically analyse both qualitative and quantitative findings for ‘evidence-based patient-oriented healthcare’ (70). Building on Salm Ward’s study (69), this review expanded the scope to include parents, caregivers, challenges, solutions, priority group needs, and implications for health professional support, offering a contemporary perspective on infant mortality prevention. An expert librarian guided the search strategy to ensure inclusion of relevant evidence since 2013, and the QuADS tool assessed the methodological quality of diverse studies (71).

Notable limitations are the focus on English-language publications, which may bias the review towards WEIRD populations, and an absence of broader cultural wisdom and perspectives. Some study samples were un-representative of their target parent cohorts (e.g., 97% of participants were mothers rather than broader ‘parent’ or ‘caregiver’ cohorts described in study aims and conclusions), with a considerable proportion of studies lacking detailed description of recruitment outcomes (*n* = 21, 35% scored ≤2, QuADS tool-Question 9, Supplementary Table C) potentially contributing to sampling bias. Although approaches to shared sleep messaging vary in the literature, many study authors did not state their assumptions, instead implicitly adopting a risk elimination lens that shaped interpretations of caregiver ‘noncompliance’ and information needs. Although the QuADS tool assessed study quality, no cut-off level was established for exclusion, resulting in varying levels of methodological rigor and transparency in reporting across studies.

## 5 Conclusion

Current safer sleep guidelines often assume that shared sleep is a deliberate decision, yet the insights from this review highlight the critical mismatch between policy assumptions and real-world behaviour. Shared sleep is common for many families and embracing this reality with proactive education and guidelines is vital to ensure the safety of infants, day and night. Families deserve support that reflects their lived experiences. This review has highlighted that parents are willing and able to co-create evidence-based resources, public health campaigns and strategies that empower parents with the knowledge they need to make shared sleep safer. Evidence from this review challenges researchers, policy makers and health professionals to shift their focus from risk elimination approaches by acknowledging the prevalence of shared sleep, fostering collaboration with consumers, and prioritising co-designed risk minimisation education that meets the needs of contemporary parents. In doing so, we can make safer shared sleep a shared priority wherever, and whenever, it occurs.
