# Supplementary material for: Beyond the rules: an integrative review of parental perspectives on safer infant sleep in shared environments
Source: Front Public Health. 2025 Sep 15;13:1629678. doi: 10.3389/fpubh.2025.1629678 (PMC12477138; doi:10.3389/fpubh.2025.1629678)
Supplement: Supplementary file 1 [file Data_Sheet_1.PDF]

Supplemental Table A: Key approaches to shared sleep in safe sleep policy and guidelines

Approach + Definition	Examples of language used in safe infant sleep policy and resources
**Risk Elimination as instruction-**<br>"An approach that proposes parents are informed not to bed-share or co-sleep with their infant under any circumstances. Not supported by current evidence and is often not practical or adhered to by parents."(Queensland Clinical Guidelines, 2022)	American Academy of Pediatrics:<br>• "the AAP is unable to recommend bed sharing under any circumstances."<br>• "Never sleep with your baby"<br>• Based on the evidence, the AAP doesn't recommend bed sharing with your baby under any circumstances. This includes twins and other multiples." (Moon, 2023)<br>New Zealand Ministry of Health:<br>• "It is never safe to put your baby to sleep in an adult bed, on a couch or on a chair." (HealthEd., 2023)
**Risk elimination framed as best practice acknowledging shared sleep may happen**<br>An approach that proposes parents are informed not to bed-share or co-sleep with their infant but then adds risk reduction strategies as a precaution.	Lullaby Trust:<br>• "The safest place for a baby to sleep is in their own separate sleep space such as a cot or Moses basket, free from toys, blankets and pillows. This helps lower the risk of sudden infant death syndrome (SIDS). But many parents find themselves co-sleeping whether they mean to or they fall asleep together unintentionally.<br>Wherever you're planning for your baby to sleep, we recommend making your bed as safe as possible for your baby**.** This page offers advice and guidance on how to create a safer environment for sleep, when to avoid co-sleeping completely, and how to reduce the risk of SIDS." (The Lullaby Trust, 2025)<br>National Health Service:<br>• "It's always safer to let your baby sleep in their own cot or Moses basket in the same room as you."<br>• "You should never co-sleep with your baby if you are extremely tired, or your baby has a fever or any signs of illness."<br>• It is not recommended to co-sleep with your baby if they were born premature (before 37 weeks) or had a low birthweight (less than 2.5kg or 5.5lb). (National Health Service, 2025)<br>Red Nose Australia:<br>• "The safest place for a baby is in their own safe sleep space, we do not recommend co-sleeping. However, if you do co-sleep, it is important for you to know the safest way to do it." (Red Nose Australia, 2024)
**Risk Minimisation-**<br>"An approach that advocates an individual family's circumstances are considered when providing advice about the infant care practices parents use in caring for their infant. Supports the recommendation that parents are provided with information that includes benefits and strategies to reduce the risk and increase safety associated with all infant sleep environments, including shared sleeping "(Queensland Clinical Guidelines, 2022)	Academy of Breastfeeding Medicine, protocol:<br>• "There is currently not enough evidence to support routine recommendations against co-sleeping. Parents should be educated about risks and benefits of co-sleeping and unsafe co-sleeping practices and should be allowed to make their own informed decision." (Blair et al., 2020)<br>Australian College of Midwives, position statement:<br>• "In consideration of the many documented benefits of shared sleeping, the need to promote and support breastfeeding, the high prevalence of shared sleep environments in contemporary Australian society and the right of parents to make informed choices about their baby's care, the Australian College of Midwives supports the recommendation that parents should be provided with information that includes benefits, risks and strategies to reduce the risk and increase safety associated with shared sleep environments, should they decide or have no option but to share a sleep surface with their baby." (Australian College of Midwives, 2014)<br>National Institute for Health and Care Excellence (NICE), postnatal guideline:<br>• "Discuss with parents safer practices for bed sharing, including:<br>o making sure the baby sleeps on a firm, flat mattress, lying face up (rather than face down or on their side)<br>o not sleeping on a sofa or chair with the baby<br>o not having pillows or duvets near the baby<br>o not having other children or pets in the bed when sharing a bed with a baby."(National Institute for Health and Care Excellence, 2021)<br>Queensland Clinical Guidelines:<br>• "Sharing sleep with an infant is a common and valued infant care practice in most cultures. Families share sleep with their infant for cultural, social and personal reasons. Shared sleep may be intentional, or occur unintentionally, if the caregiver falls asleep or has no other options to use as an infant sleep space." (Queensland Clinical Guidelines, 2022)<br>UNICEF UK<br>• "Some parents choose to sleep with their baby in bed and some fall asleep with their baby during the night while feeding and comforting – whether they intend to or not. Therefore, it is very important to consider the following points:" (UNICEF UK, 2022)

Supplemental Table B: Research Question and Search Strategy

**Search strategy string - PICo**		**Adapted strategy building on Salm Ward 2015**
Population	Sample cohort terms<br>Study type terms	Infant OR caregiver* OR parent* OR mother* OR maternal OR women OR woman OR paternal OR father*
		AND
	Study type terms	
Interest	Shared sleep terms	Cosleep* OR co-sleep* OR bedshar*OR sofashar* OR “shared sleep” OR roomshar* OR “room shar*” OR “sleep location*” OR “sleep practice*” OR “sleep arrangement*”
		AND
Context	Uptake and engagement with risk minimisation approaches for shared sleep (challenges faced creating shared sleeping environments, solutions or strategies applied to address these challenges, what information did they want or need, how is this different for families with additional risk factors (smoking, preterm, low birth weight, formula fed, drugs/ alcohol)	Concern OR strateg* OR challeng* OR risk factor*
Limits:<br>Dates- 2013-March 13, 2025<br>Language: English		N.B. * represents truncation
Databases: Scopus, CINAHL, PubMed, PsycNET, Emcare		

Inclusion criteria:

a) Empirical, peer reviewed studies, including systematically conducted literature reviews and publicly available final theses, published between January 2013-13 March 2025 in the English language, and
b) referred to bed-sharing with infants less than 12 months of age (inclusive of vulnerable groups: including multiple births, co-bedding when referring to caregiver-infant bed-sharing); and
c) provided perceptions/perspectives of primary caregivers relating to reasons for primary caregiver-infant bed-sharing (these 'reasons' may include challenges and but may also be 'solutions' to other issues, including caring for infants with medical complexity/health challenges); and/or
d) provided challenges associated with bed-sharing (included challenges of 'not bed-sharing', challenges when bed-sharing); and/or provided strategies/solutions to address challenges experienced.

Exclusion criteria:

a) papers published before January 2013; as the preceding two decades (1990-2013) of literature were the focus of the earlier Salm Ward (2015) review.
b) papers not available in the English language,
c) discussion papers or general opinion papers, and clinical guidelines.

Supplemental Table C: QuADS tool

Study/ Criteria	1. Theoretical or conceptual underpinning to the research	2. Statement of research aim/s	3. Clear description of research setting and target population	4. The study design is appropriate to address the stated research aim/s	5. Appropriate sampling to address the research aim/s	6. Rationale for choice of data collection tool/s	7. The format and content of data collection tool is appropriate to address the stated research aim/s	8. Description of data collection procedure	9. Recruitment data provided	10. Justification for analytic method selected	11. The method of analysis was appropriate to answer the research aim/s	12. Evidence that the research stakeholders have been considered in research design or conduct.	13. Strengths and limitations critically discussed
Bailey, C. (2016). Breastfeeding mothers' experiences of bedsharing: A qualitative study. Breastfeeding Review, 24(2), 33-40. https://search.informit.org/doi/10.3316/ielapa.268164166573611	1. General reference to broad theories or concepts that frame the study. e.g. key concepts were identified in the introduction section.	3. Explicit and detailed statement of aims/objectives in main body of report.	3. Specific description of the research setting and target population of study e.g. 'nurses and doctors from GP practices in	3. The study design selected appears to be the most suitable approach to attempt to answer the stated research aim/s.	3. Detailed evidence of consideration of the sample required to address the research aim/s. e.g. sample size calculation or discussion of an iterative	1. Very limited explanation for choice of data collection tool/s. e.g. based on availability of tool.	2. Structure and/or content of tool/s allow for data to be gathered broadly addressing the stated aim/s but could benefit from	2. States each stage of data collection procedure but with limited detail or states some stages in detail but omits others	2. Some recruitment data but not a complete account e.g. number of people who were invited and agreed.	1. Very limited justification for choice of analytic method selected. e.g. previous use by the research team.	3. Method of analysis selected is the most suitable approach to attempt answer the research aim/s in detail e.g. for qualitative interpretative phenomenological analysis might be considered	0. No mention at all	0. No mention at all
Bailey, C., Tawia, S., & McGuire, E. (2020). Breastfeeding Duration and Infant Sleep Location in a Cohort of Volunteer Breastfeeding Counselors. J Hum Lact, 36(2), 354-364. https://doi.org/10.1177/0890334419851801	3. Explicit discussion of the theories or concepts that inform the study, with application of the theory or concept evident through the design,	3. Explicit and detailed statement of aims/objectives in main body of report.	3. Specific description of the research setting and target population of study e.g. 'nurses and doctors from GP practices in	3. The study design selected appears to be the most suitable approach to attempt to answer the stated research aim/s.	3. Detailed evidence of consideration of the sample required to address the research aim/s. e.g. sample size calculation or discussion of an iterative	3. Detailed explanation of rationale for choice of data collection tool/s. e.g. relevance to the study aim/s, co-designed with the	3. Structure and content of tool/s allow for detailed data to be gathered around all relevant issues required to address the	3. Detailed description of each stage of the data collection procedure, including when, where and how data was gathered such that the	2. Some recruitment data but not a complete account e.g. number of people who were invited and agreed.	3. Detailed justification for choice of analytic method selected e.g. relevance to the study aim/s or comment around of the strengths	3. Method of analysis selected is the most suitable approach to attempt answer the research aim/s in detail e.g. for qualitative interpretative phenomenological analysis might be considered	2. Evidence of stakeholder input informing the research. e.g. use of pilot study with feedback influencing the study design/conduct or reference to a project reference	3. Thorough discussion of strengths and limitations of all aspects of study including design, methods, data collection tools, sample & analytic approach.
Ball, H. L., Taylor, C. E., & Yuill, C. M. (2021). A box to put the baby in: UK parent perceptions of two baby box programmes promoted for infant sleep [Article]. International Journal of Environmental Research and Public Health, 18(21), Article 11473. https://doi.org/10.3390/ijerph182111473	2. Identification of specific theories or concepts that frame the study and how these informed the work undertaken. e.g. key	2. Aims statement made but may only appear in the abstract or be lacking detail.	2. Description of research setting is made but is lacking detail e.g. 'in primary care practices in region [x]'.	1. The study design can only address some aspects of the stated research aim/s e.g. use of focus groups to capture data	1. Evidence of consideration of the sample required e.g. the sample characteristics are described and appear appropriate to address the	0. No mention of rationale for data collection tool used.	0. No research aim/s stated and/or data collection tool not detailed.	1. Basic and brief outline of data collection procedure e.g. 'using a questionnaire distributed to staff'.	2. Some recruitment data but not a complete account e.g. number of people who were invited and agreed.	0. No mention of the rationale for the analytic method chosen.	1. Method of analysis can only address the research aim/s basically or broadly.	0. No mention at all	2. Discussion of some of the key strengths and weaknesses of the study but not complete. e.g. several strengths/limitations explored but with notable omissions or lack of depth of explanation.
Bamber, A. R., Kiho, L., Upton, S., Orchard, M., & Sebire, N. J. (2016). Social and behavioural factors in Non-suspicious unexpected death in infancy; experience from metropolitan police project indigo investigation [Article]. BMC Pediatrics, 16(1), Article 6. https://doi.org/10.1186/s12887-016-0541-x	3. Explicit discussion of the theories or concepts that inform the study, with application of the theory or concept evident through the design,	3. Explicit and detailed statement of aims/objectives in main body of report.	3. Specific description of the research setting and target population of study e.g. 'nurses and doctors from GP practices in	3. The study design selected appears to be the most suitable approach to attempt to answer the stated research aim/s.	2. Evidence of consideration of sample required to address the aim. e.g. the sample characteristics are described with	3. Detailed explanation of rationale for choice of data collection tool/s. e.g. relevance to the study aim/s, co-designed with the	3. Structure and content of tool/s allow for detailed data to be gathered around all relevant issues required to address the	3. Detailed description of each stage of the data collection procedure, including when, where and how data was gathered such that the	3. Complete data allowing for full picture of recruitment outcomes e.g. number of people approached, recruited, and who	3. Detailed justification for choice of analytic method selected e.g. relevance to the study aim/s or comment around of the strengths	3. Method of analysis selected is the most suitable approach to attempt answer the research aim/s in detail e.g. for qualitative interpretative phenomenological analysis might be considered	0. No mention at all	3. Thorough discussion of strengths and limitations of all aspects of study including design, methods, data collection tools, sample & analytic approach.

Barrett, S., Barlow, J., Cann, H., Pease, A., Shiells, K., Woodman, J., & McGovern, R. (2024). Parental decision making about safer sleep practices: A qualitative study of the perspectives of families with additional health and social care needs.	3. Explicit discussion of the theories or concepts that inform the study, with application of the theory or concept evident through the design,	3. Explicit and detailed statement of aims/objectives in main body of report.	3. Specific description of the research setting and target population of study e.g. 'nurses and doctors from GP practices in	3. The study design selected appears to be the most suitable approach to attempt to answer the stated research aim/s.	2. Evidence of consideration of sample required to address the aim. e.g. the sample characteristics are described with	3. Detailed explanation of rationale for choice of data collection tool/s. e.g. relevance to the study aim/s, co-designed with the	3. Structure and content of tool/s allow for detailed data to be gathered around all relevant issues required to address the	3. Detailed description of each stage of the data collection procedure, including when, where and how data was gathered such that the	2. Some recruitment data but not a complete account e.g. number of people who were invited and agreed.	3. Detailed justification for choice of analytic method selected e.g. relevance to the study aim/s or comment around of the strengths	3. Method of analysis selected is the most suitable approach to attempt answer the research aim/s in detail e.g. for qualitative interpretative phenomenological analysis might be considered	0. No mention at all	3. Thorough discussion of strengths and limitations of all aspects of study including design, methods, data collection tools, sample & analytic approach.
Barry, E. S., & McKenna, J. J. (2022). Reasons mothers bedshare: A review of its effects on infant behavior and development. Infant Behavior and Development, 66, 101684. https://doi.org/https://doi.org/10.1016/j.infbeh.2021.101684	3. Explicit discussion of the theories or concepts that inform the study, with application of the theory or concept evident through the design,	3. Explicit and detailed statement of aims/objectives in main body of report.	0. No mention at all	3. The study design selected appears to be the most suitable approach to attempt to answer the stated research aim/s.	0. No mention of the sampling approach.	1. Very limited explanation for choice of data collection tool/s. e.g. based on availability of tool.	1. Structure and/or content of tool/s suitable to address some aspects of the research aim/s or to address the aim/s	0. No mention of the data collection procedure.	0. No mention of recruitment data.	3. Detailed justification for choice of analytic method selected e.g. relevance to the study aim/s or comment around of the strengths	3. Method of analysis selected is the most suitable approach to attempt answer the research aim/s in detail e.g. for qualitative interpretative phenomenological analysis might be considered	0. No mention at all	0. No mention at all
Beth Howard, M., Parrish, B. T., Singletary, J., & Jarvis, L. (2022). Infant Safe Sleep in the District of Columbia: Better for Both. Health Promotion Practice. https://doi.org/https://dx.doi.org/10.1177/15248399221131834	3. Explicit discussion of the theories or concepts that inform the study, with application of the theory or concept evident through the design,	3. Explicit and detailed statement of aims/objectives in main body of report.	3. Specific description of the research setting and target population of study e.g. 'nurses and doctors from GP practices in	3. The study design selected appears to be the most suitable approach to attempt to answer the stated research aim/s.	1. Evidence of consideration of the sample required e.g. the sample characteristics are described and appear appropriate to address the	3. Detailed explanation of rationale for choice of data collection tool/s. e.g. relevance to the study aim/s, co-designed with the	3. Structure and content of tool/s allow for detailed data to be gathered around all relevant issues required to address the	3. Detailed description of each stage of the data collection procedure, including when, where and how data was gathered such that the	3. Complete data allowing for full picture of recruitment outcomes e.g. number of people approached, recruited, and who	3. Detailed justification for choice of analytic method selected e.g. relevance to the study aim/s or comment around of the strengths	3. Method of analysis selected is the most suitable approach to attempt answer the research aim/s in detail e.g. for qualitative interpretative phenomenological analysis might be considered	0. No mention at all	3. Thorough discussion of strengths and limitations of all aspects of study including design, methods, data collection tools, sample & analytic approach.
Capper, B., Damato, E. G., Gutin-Barsman, S., & Dowling, D. (2022). Mothers' Decision Making Concerning Safe Sleep for Preterm Infants: What Are the Influencing Factors? Adv Neonatal Care, 22(5), 444-455. https://doi.org/10.1097/anc.0000000000000952	3. Explicit discussion of the theories or concepts that inform the study, with application of the theory or concept evident through the design,	3. Explicit and detailed statement of aims/objectives in main body of report.	2. Description of research setting is made but is lacking detail e.g. 'in primary care practices in region [x]'.	3. The study design selected appears to be the most suitable approach to attempt to answer the stated research aim/s.	2. Evidence of consideration of sample required to address the aim. e.g. the sample characteristics are described with	3. Detailed explanation of rationale for choice of data collection tool/s. e.g. relevance to the study aim/s, co-designed with the	3. Structure and content of tool/s allow for detailed data to be gathered around all relevant issues required to address the	3. Detailed description of each stage of the data collection procedure, including when, where and how data was gathered such that the	3. Complete data allowing for full picture of recruitment outcomes e.g. number of people approached, recruited, and who	0. No mention of the rationale for the analytic method chosen.	3. Method of analysis selected is the most suitable approach to attempt answer the research aim/s in detail e.g. for qualitative interpretative phenomenological analysis might be considered	0. No mention at all	3. Thorough discussion of strengths and limitations of all aspects of study including design, methods, data collection tools, sample & analytic approach.
Caraballo, M., Shimasaki, S., Johnston, K., Tung, G., Albright, K., & Halbower, A. C. (2016). Knowledge, Attitudes, and Risk for Sudden Unexpected Infant Death in Children of Adolescent Mothers: A Qualitative Study. Journal of Pediatrics, 174, 78-83.e72. https://doi.org/10.1016/j.jpeds.2016.03.031	3. Explicit discussion of the theories or concepts that inform the study, with application of the theory or concept evident through the design,	3. Explicit and detailed statement of aims/objectives in main body of report.	3. Specific description of the research setting and target population of study e.g. 'nurses and doctors from GP practices in	3. The study design selected appears to be the most suitable approach to attempt to answer the stated research aim/s.	3. Detailed evidence of consideration of the sample required to address the research aim/s. e.g. sample size calculation or discussion of an iterative	2. Basic explanation of rationale for choice of data collection tool/s. e.g. based on use in a prior similar study.	3. Structure and content of tool/s allow for detailed data to be gathered around all relevant issues required to address the	3. Detailed description of each stage of the data collection procedure, including when, where and how data was gathered such that the	2. Some recruitment data but not a complete account e.g. number of people who were invited and agreed.	3. Detailed justification for choice of analytic method selected e.g. relevance to the study aim/s or comment around of the strengths	3. Method of analysis selected is the most suitable approach to attempt answer the research aim/s in detail e.g. for qualitative interpretative phenomenological analysis might be considered	0. No mention at all	3. Thorough discussion of strengths and limitations of all aspects of study including design, methods, data collection tools, sample & analytic approach.
Clarke, J. (2016). Velcro babies: A Qualitative Study Exploring Maternal Motivations in the Night-time Care of Infants University of Otago]. http://hdl.handle.net/10523/6458	3. Explicit discussion of the theories or concepts that inform the study, with application of the theory or concept evident through the design,	3. Explicit and detailed statement of aims/objectives in main body of report.	3. Specific description of the research setting and target population of study e.g. 'nurses and doctors from GP practices in	3. The study design selected appears to be the most suitable approach to attempt to answer the stated research aim/s.	3. Detailed evidence of consideration of the sample required to address the research aim/s. e.g. sample size calculation or discussion of an iterative	3. Detailed explanation of rationale for choice of data collection tool/s. e.g. relevance to the study aim/s, co-designed with the	3. Structure and content of tool/s allow for detailed data to be gathered around all relevant issues required to address the	3. Detailed description of each stage of the data collection procedure, including when, where and how data was gathered such that the	3. Complete data allowing for full picture of recruitment outcomes e.g. number of people approached, recruited, and who	3. Detailed justification for choice of analytic method selected e.g. relevance to the study aim/s or comment around of the strengths	3. Method of analysis selected is the most suitable approach to attempt answer the research aim/s in detail e.g. for qualitative interpretative phenomenological analysis might be considered	0. No mention at all	2. Discussion of some of the key strengths and weaknesses of the study but not complete. e.g. several strengths/limitations explored but with notable omissions or lack of depth of explanation.

Cole, R., Young, J., Kearney, L., & Thompson, J. M. D. (2021). Challenges parents encounter when implementing infant safe sleep advice. Acta Paediatr, 110(11), 3083-3093. https://doi.org/10.1111/apa.16040	3. Explicit discussion of the theories or concepts that inform the study, with application of the theory or concept evident through the design,	3. Explicit and detailed statement of aims/objectives in main body of report.	3. Specific description of the research setting and target population of study e.g. 'nurses and doctors from GP practices in	3. The study design selected appears to be the most suitable approach to attempt to answer the stated research aim/s.	1. Evidence of consideration of the sample required e.g. the sample characteristics are described and appear appropriate to address the	0. No mention of rationale for data collection tool used.	3. Structure and content of tool/s allow for detailed data to be gathered around all relevant issues required to address the	3. Detailed description of each stage of the data collection procedure, including when, where and how data was gathered such that the	3. Complete data allowing for full picture of recruitment outcomes e.g. number of people approached, recruited, and who	3. Detailed justification for choice of analytic method selected e.g. relevance to the study aim/s or comment around of the strengths	3. Method of analysis selected is the most suitable approach to attempt answer the research aim/s in detail e.g. for qualitative interpretative phenomenological analysis might be considered	0. No mention at all	2. Discussion of some of the key strengths and weaknesses of the study but not complete. e.g. several strengths/limitations explored but with notable omissions or lack of depth of explanation.
Cowan, S., Bennett, S., Clarke, J., & Pease, A. (2013). An evaluation of portable sleeping spaces for babies following the Christchurch earthquake of February 2011. Journal of Paediatrics and Child Health, 49(5), 364-368. https://doi.org/https://dx.doi.org/10.1111/jpc.12196	3. Explicit discussion of the theories or concepts that inform the study, with application of the theory or concept evident through the design,	3. Explicit and detailed statement of aims/objectives in main body of report.	3. Specific description of the research setting and target population of study e.g. 'nurses and doctors from GP practices in	3. The study design selected appears to be the most suitable approach to attempt to answer the stated research aim/s.	1. Evidence of consideration of the sample required e.g. the sample characteristics are described and appear appropriate to address the	2. Basic explanation of rationale for choice of data collection tool/s. e.g. based on use in a prior similar study.	3. Structure and content of tool/s allow for detailed data to be gathered around all relevant issues required to address the	3. Detailed description of each stage of the data collection procedure, including when, where and how data was gathered such that the	3. Complete data allowing for full picture of recruitment outcomes e.g. number of people approached, recruited, and who	0. No mention of the rationale for the analytic method chosen.	3. Method of analysis selected is the most suitable approach to attempt answer the research aim/s in detail e.g. for qualitative interpretative phenomenological analysis might be considered	0. No mention at all	1. Very limited mention of strengths and limitations with omissions of many key issues. e.g. one or two strengths/limitations mentioned with limited detail.
Crane, D., & Ball, H. L. (2016). A qualitative study in parental perceptions and understanding of SIDS-reduction guidance in a UK bi-cultural urban community. BMC Pediatrics, 16(1). https://doi.org/10.1186/s12887-016-0560-7	2. Identification of specific theories or concepts that frame the study and how these informed the work undertaken. e.g. key	3. Explicit and detailed statement of aims/objectives in main body of report.	3. Specific description of the research setting and target population of study e.g. 'nurses and doctors from GP practices in	3. The study design selected appears to be the most suitable approach to attempt to answer the stated research aim/s.	2. Evidence of consideration of sample required to address the aim. e.g. the sample characteristics are described with	2. Basic explanation of rationale for choice of data collection tool/s. e.g. based on use in a prior similar study.	2. Structure and/or content of tool/s allow for data to be gathered broadly addressing the stated aim/s but could benefit from	2. States each stage of data collection procedure but with limited detail or states some stages in detail but omits others	3. Complete data allowing for full picture of recruitment outcomes e.g. number of people approached, recruited, and who	2. Basic justification for choice of analytic method selected e.g. method used in prior similar research.	3. Method of analysis selected is the most suitable approach to attempt answer the research aim/s in detail e.g. for qualitative interpretative phenomenological analysis might be considered	0. No mention at all	0. No mention at all
Cunningham, H. M., Vally, H., & Bugeja, L. (2018). Bed-Sharing in the First 8 Weeks of Life: An Australian Study. Maternal & Child Health Journal, 22(4), 556-564. https://doi.org/10.1007/s10995-017-2424-7	3. Explicit discussion of the theories or concepts that inform the study, with application of the theory or concept evident through the design,	2. Aims statement made but may only appear in the abstract or be lacking detail.	3. Specific description of the research setting and target population of study e.g. 'nurses and doctors from GP practices in	3. The study design selected appears to be the most suitable approach to attempt to answer the stated research aim/s.	3. Detailed evidence of consideration of the sample required to address the research aim/s. e.g. sample size calculation or discussion of an iterative	3. Detailed explanation of rationale for choice of data collection tool/s. e.g. relevance to the study aim/s, co-designed with the	3. Structure and content of tool/s allow for detailed data to be gathered around all relevant issues required to address the	3. Detailed description of each stage of the data collection procedure, including when, where and how data was gathered such that the	3. Complete data allowing for full picture of recruitment outcomes e.g. number of people approached, recruited, and who	2. Basic justification for choice of analytic method selected e.g. method used in prior similar research.	3. Method of analysis selected is the most suitable approach to attempt answer the research aim/s in detail e.g. for qualitative interpretative phenomenological analysis might be considered	0. No mention at all	3. Thorough discussion of strengths and limitations of all aspects of study including design, methods, data collection tools, sample & analytic approach.
Doering, J. J., Lim, P. S., Salm Ward, T. C., & Davies, W. H. (2019). Prevalence of unintentional infant bedsharing. Applied Nursing Research, 46, 28-30. https://doi.org/10.1016/j.apnr.2019.02.003	3. Explicit discussion of the theories or concepts that inform the study, with application of the theory or concept evident through the design,	3. Explicit and detailed statement of aims/objectives in main body of report.	3. Specific description of the research setting and target population of study e.g. 'nurses and doctors from GP practices in	2. The study design can address the stated research aim/s but there is a more suitable alternative that could have been	1. Evidence of consideration of the sample required e.g. the sample characteristics are described and appear appropriate to address the	3. Detailed explanation of rationale for choice of data collection tool/s. e.g. relevance to the study aim/s, co-designed with the	2. Structure and/or content of tool/s allow for data to be gathered broadly addressing the stated aim/s but could benefit from	3. Detailed description of each stage of the data collection procedure, including when, where and how data was gathered such that the	3. Complete data allowing for full picture of recruitment outcomes e.g. number of people approached, recruited, and who	3. Detailed justification for choice of analytic method selected e.g. relevance to the study aim/s or comment around of the strengths	3. Method of analysis selected is the most suitable approach to attempt answer the research aim/s in detail e.g. for qualitative interpretative phenomenological analysis might be considered	0. No mention at all	3. Thorough discussion of strengths and limitations of all aspects of study including design, methods, data collection tools, sample & analytic approach.
Doering, J. J., Marvin, A., & Strook, S. (2017). Parent decision factors, safety strategies, and fears about infant sleep locations. Applied Nursing Research, 34, 29-33. https://doi.org/10.1016/j.apnr.2017.01.002	2. Identification of specific theories or concepts that frame the study and how these informed the work undertaken. e.g. key	3. Explicit and detailed statement of aims/objectives in main body of report.	3. Specific description of the research setting and target population of study e.g. 'nurses and doctors from GP practices in	3. The study design selected appears to be the most suitable approach to attempt to answer the stated research aim/s.	1. Evidence of consideration of the sample required e.g. the sample characteristics are described and appear appropriate to address the	3. Detailed explanation of rationale for choice of data collection tool/s. e.g. relevance to the study aim/s, co-designed with the	3. Structure and content of tool/s allow for detailed data to be gathered around all relevant issues required to address the	3. Detailed description of each stage of the data collection procedure, including when, where and how data was gathered such that the	2. Some recruitment data but not a complete account e.g. number of people who were invited and agreed.	3. Detailed justification for choice of analytic method selected e.g. relevance to the study aim/s or comment around of the strengths	3. Method of analysis selected is the most suitable approach to attempt answer the research aim/s in detail e.g. for qualitative interpretative phenomenological analysis might be considered	2. Evidence of stakeholder input informing the research. e.g. use of pilot study with feedback influencing the study design/conduct or reference to a project reference	2. Discussion of some of the key strengths and weaknesses of the study but not complete. e.g. several strengths/limitations explored but with notable omissions or lack of depth of explanation.

Ellis, C. (2019). Safely Sleeping? : an Exploration of Mothers' Understanding of Safe Sleep Practices and Factors that Influence Reducing Risks in Their Infant's Sleep Environment (Publication Number 28125218) [Ph.D., University of Warwick (United Kingdom)]. ProQuest One Academic. England.	3. Explicit discussion of the theories or concepts that inform the study, with application of the theory or concept evident through the design,	3. Explicit and detailed statement of aims/objectives in main body of report.	3. Specific description of the research setting and target population of study e.g. 'nurses and doctors from GP practices in	3. The study design selected appears to be the most suitable approach to attempt to answer the stated research aim/s.	3. Detailed evidence of consideration of the sample required to address the research aim/s. e.g. sample size calculation or discussion of an iterative	3. Detailed explanation of rationale for choice of data collection tool/s. e.g. relevance to the study aim/s, co-designed with the	3. Structure and content of tool/s allow for detailed data to be gathered around all relevant issues required to address the	3. Detailed description of each stage of the data collection procedure, including when, where and how data was gathered such that the	3. Complete data allowing for full picture of recruitment outcomes e.g. number of people approached, recruited, and who	3. Detailed justification for choice of analytic method selected e.g. relevance to the study aim/s or comment around of the strengths	3. Method of analysis selected is the most suitable approach to attempt answer the research aim/s in detail e.g. for qualitative interpretative phenomenological analysis might be considered	3. Substantial consultation with stakeholders identifiable in planning of study design and in preliminary work e.g. consultation in the conceptualisatio	3. Thorough discussion of strengths and limitations of all aspects of study including design, methods, data collection tools, sample & analytic approach.
Fangupo, L. J., Lucas, A. W., Taylor, R. W., Camp, J., & Richards, R. (2021). Sleep and parenting in ethnically diverse Pacific families in southern New Zealand: A qualitative exploration. Sleep Health, 668. https://doi.org/https://dx.doi.org/10.1016/j.sleh.2021.11.002	3. Explicit discussion of the theories or concepts that inform the study, with application of the theory or concept evident through the design,	3. Explicit and detailed statement of aims/objectives in main body of report.	3. Specific description of the research setting and target population of study e.g. 'nurses and doctors from GP practices in	3. The study design selected appears to be the most suitable approach to attempt to answer the stated research aim/s.	2. Evidence of consideration of sample required to address the aim. e.g. the sample characteristics are described with	3. Detailed explanation of rationale for choice of data collection tool/s. e.g. relevance to the study aim/s, co-designed with the	3. Structure and content of tool/s allow for detailed data to be gathered around all relevant issues required to address the	3. Detailed description of each stage of the data collection procedure, including when, where and how data was gathered such that the	2. Some recruitment data but not a complete account e.g. number of people who were invited and agreed.	0. No mention of the rationale for the analytic method chosen.	3. Method of analysis selected is the most suitable approach to attempt answer the research aim/s in detail e.g. for qualitative interpretative phenomenological analysis might be considered	1. Consideration of some the research stakeholders e.g. use of pilot study with target sample but no stakeholder involvement in planning stages of study design.	3. Thorough discussion of strengths and limitations of all aspects of study including design, methods, data collection tools, sample & analytic approach.
Feld, H., Ceballos Osorio, J., Bahamonde, M., Young, T., Boada, P., & Rayens, M. K. (2021). Poverty and Paternal Education Associated With Infant Safe Sleep Intentions in a Peri-Urban Community in Ecuador. Glob Pediatr Health, 8, 2333794x211044112. https://doi.org/10.1177/2333794x211044112	2. Identification of specific theories or concepts that frame the study and how these informed the work undertaken. e.g. key	3. Explicit and detailed statement of aims/objectives in main body of report.	3. Specific description of the research setting and target population of study e.g. 'nurses and doctors from GP practices in	3. The study design selected appears to be the most suitable approach to attempt to answer the stated research aim/s.	3. Detailed evidence of consideration of the sample required to address the research aim/s. e.g. sample size calculation or discussion of an iterative	3. Detailed explanation of rationale for choice of data collection tool/s. e.g. relevance to the study aim/s, co-designed with the	2. Structure and/or content of tool/s allow for data to be gathered broadly addressing the stated aim/s but could benefit from	3. Detailed description of each stage of the data collection procedure, including when, where and how data was gathered such that the	2. Some recruitment data but not a complete account e.g. number of people who were invited and agreed.	3. Detailed justification for choice of analytic method selected e.g. relevance to the study aim/s or comment around of the strengths	3. Method of analysis selected is the most suitable approach to attempt answer the research aim/s in detail e.g. for qualitative interpretative phenomenological analysis might be considered	0. No mention at all	3. Thorough discussion of strengths and limitations of all aspects of study including design, methods, data collection tools, sample & analytic approach.
Gaertner, V. D., Malfertheiner, S. F., Postpischil, J., Brandstetter, S., Seelbach-Göbel, B., Apfelbacher, C., Melter, M., Kabesch, M., Ambrosch, A., Arndt, P. A., Baessler, A., Berneburg, M., Böse-O'Reilly, S., Brunner, R., Buchalla, W., Franke, A., Häusler, S., Heid, I., Heinze, S., . . . Wellmann, S. (2023). Implementation of safe infant sleep recommendations during night-time sleep in the first year of life in a German birth cohort [Article]. Scientific Reports, 13(1), Article 875. https://doi.org/10.1038/s41598-023-28008-1	2. Identification of specific theories or concepts that frame the study and how these informed the work undertaken. e.g. key	3. Explicit and detailed statement of aims/objectives in main body of report.	3. Specific description of the research setting and target population of study e.g. 'nurses and doctors from GP practices in	3. The study design selected appears to be the most suitable approach to attempt to answer the stated research aim/s.	3. Detailed evidence of consideration of the sample required to address the research aim/s. e.g. sample size calculation or discussion of an iterative	3. Detailed explanation of rationale for choice of data collection tool/s. e.g. relevance to the study aim/s, co-designed with the	3. Structure and content of tool/s allow for detailed data to be gathered around all relevant issues required to address the	2. States each stage of data collection procedure but with limited detail or states some stages in detail but omits others	2. Some recruitment data but not a complete account e.g. number of people who were invited and agreed.	3. Detailed justification for choice of analytic method selected e.g. relevance to the study aim/s or comment around of the strengths	3. Method of analysis selected is the most suitable approach to attempt answer the research aim/s in detail e.g. for qualitative interpretative phenomenological analysis might be considered	0. No mention at all	3. Thorough discussion of strengths and limitations of all aspects of study including design, methods, data collection tools, sample & analytic approach.
Gaydos, L. M., Blake, S. C., Gazmararian, J. A., Woodruff, W., Thompson, W. W., & Dalmida, S. G. (2015). Revisiting Safe Sleep Recommendations for African-American Infants: Why Current Counseling is Insufficient. Maternal and Child Health Journal, 19(3), 496-503. https://doi.org/https://dx.doi.org/10.1007/s10995-014-1530-z	3. Explicit discussion of the theories or concepts that inform the study, with application of the theory or concept evident through the design,	3. Explicit and detailed statement of aims/objectives in main body of report.	3. Specific description of the research setting and target population of study e.g. 'nurses and doctors from GP practices in	3. The study design selected appears to be the most suitable approach to attempt to answer the stated research aim/s.	3. Detailed evidence of consideration of the sample required to address the research aim/s. e.g. sample size calculation or discussion of an iterative	3. Detailed explanation of rationale for choice of data collection tool/s. e.g. relevance to the study aim/s, co-designed with the	3. Structure and content of tool/s allow for detailed data to be gathered around all relevant issues required to address the	3. Detailed description of each stage of the data collection procedure, including when, where and how data was gathered such that the	3. Complete data allowing for full picture of recruitment outcomes e.g. number of people approached, recruited, and who	3. Detailed justification for choice of analytic method selected e.g. relevance to the study aim/s or comment around of the strengths	3. Method of analysis selected is the most suitable approach to attempt answer the research aim/s in detail e.g. for qualitative interpretative phenomenological analysis might be considered	2. Evidence of stakeholder input informing the research. e.g. use of pilot study with feedback influencing the study design/conduct or reference to a project reference	0. No mention at all
George, M., Theodore, R., Richards, R., Galland, B., Taylor, R., Matahaere, M., & Te Morenga, L. (2020). Moe Kitenga: a qualitative study of perceptions of infant and child sleep practices among Māori whānau [Article]. AlterNative, 16(2), 153-160. https://doi.org/10.1177/1177180120929694	3. Explicit discussion of the theories or concepts that inform the study, with application of the theory or concept evident through the design,	3. Explicit and detailed statement of aims/objectives in main body of report.	3. Specific description of the research setting and target population of study e.g. 'nurses and doctors from GP practices in	3. The study design selected appears to be the most suitable approach to attempt to answer the stated research aim/s.	1. Evidence of consideration of the sample required e.g. the sample characteristics are described and appear appropriate to address the	2. Basic explanation of rationale for choice of data collection tool/s. e.g. based on use in a prior similar study.	3. Structure and content of tool/s allow for detailed data to be gathered around all relevant issues required to address the	3. Detailed description of each stage of the data collection procedure, including when, where and how data was gathered such that the	2. Some recruitment data but not a complete account e.g. number of people who were invited and agreed.	0. No mention of the rationale for the analytic method chosen.	3. Method of analysis selected is the most suitable approach to attempt answer the research aim/s in detail e.g. for qualitative interpretative phenomenological analysis might be considered	2. Evidence of stakeholder input informing the research. e.g. use of pilot study with feedback influencing the study design/conduct or reference to a project reference	0. No mention at all

Gettler, L. T., Kuo, P. X., Sarma, M. S., Lefever, J. E. B., Cummings, E. M., McKenna, J. J., & Braungart-Rieker, J. M. (2021). US fathers' reports of bonding, infant temperament and psychosocial stress based on family sleep arrangements. Evolution, Medicine, and Public Health, 9(1), 460-469. https://doi.org/10.1093/emph/eoab038	3. Explicit discussion of the theories or concepts that inform the study, with application of the theory or concept evident through the design,	3. Explicit and detailed statement of aims/objectives in main body of report.	2. Description of research setting is made but is lacking detail e.g. 'in primary care practices in region [x]'.	3. The study design selected appears to be the most suitable approach to attempt to answer the stated research aim/s.	3. Detailed evidence of consideration of the sample required to address the research aim/s. e.g. sample size calculation or discussion of an iterative	3. Detailed explanation of rationale for choice of data collection tool/s. e.g. relevance to the study aim/s, co-designed with the	3. Structure and content of tool/s allow for detailed data to be gathered around all relevant issues required to address the	3. Detailed description of each stage of the data collection procedure, including when, where and how data was gathered such that the	3. Complete data allowing for full picture of recruitment outcomes e.g. number of people approached, recruited, and who	3. Detailed justification for choice of analytic method selected e.g. relevance to the study aim/s or comment around of the strengths	3. Method of analysis selected is the most suitable approach to attempt answer the research aim/s in detail e.g. for qualitative interpretative phenomenological analysis might be considered	0. No mention at all	3. Thorough discussion of strengths and limitations of all aspects of study including design, methods, data collection tools, sample & analytic approach.
Gilmour, H., Ramage-Morin, P. L., & Wong, S. L. (2019). Infant bed sharing in Canada [Article]. Health Reports, 30(7), 14-19. https://doi.org/10.25318/82-003-x201900700002-eng	1. General reference to broad theories or concepts that frame the study. e.g. key concepts were identified in the introduction section.	3. Explicit and detailed statement of aims/objectives in main body of report.	3. Specific description of the research setting and target population of study e.g. 'nurses and doctors from GP practices in	2. The study design can address the stated research aim/s but there is a more suitable alternative that could have been	2. Evidence of consideration of sample required to address the aim. e.g. the sample characteristics are described with	3. Detailed explanation of rationale for choice of data collection tool/s. e.g. relevance to the study aim/s, co-designed with the	2. Structure and/or content of tool/s allow for data to be gathered broadly addressing the stated aim/s but could benefit from	1. Basic and brief outline of data collection procedure e.g. 'using a questionnaire distributed to staff'.	2. Some recruitment data but not a complete account e.g. number of people who were invited and agreed.	3. Detailed justification for choice of analytic method selected e.g. relevance to the study aim/s or comment around of the strengths	3. Method of analysis selected is the most suitable approach to attempt answer the research aim/s in detail e.g. for qualitative interpretative phenomenological analysis might be considered	0. No mention at all	3. Thorough discussion of strengths and limitations of all aspects of study including design, methods, data collection tools, sample & analytic approach.
Gustafsson, S., Jacobzon, A., Lindberg, B., & Engström, Å. (2022). Parents' strategies and advice for creating a positive sleep situation in the family. Scandinavian Journal of Caring Sciences, 36(3), 830-838. https://doi.org/10.1111/scs.13020	3. Explicit discussion of the theories or concepts that inform the study, with application of the theory or concept evident through the design,	3. Explicit and detailed statement of aims/objectives in main body of report.	3. Specific description of the research setting and target population of study e.g. 'nurses and doctors from GP practices in	3. The study design selected appears to be the most suitable approach to attempt to answer the stated research aim/s.	2. Evidence of consideration of sample required to address the aim. e.g. the sample characteristics are described with	3. Detailed explanation of rationale for choice of data collection tool/s. e.g. relevance to the study aim/s, co-designed with the	3. Structure and content of tool/s allow for detailed data to be gathered around all relevant issues required to address the	3. Detailed description of each stage of the data collection procedure, including when, where and how data was gathered such that the	3. Complete data allowing for full picture of recruitment outcomes e.g. number of people approached, recruited, and who	3. Detailed justification for choice of analytic method selected e.g. relevance to the study aim/s or comment around of the strengths	3. Method of analysis selected is the most suitable approach to attempt answer the research aim/s in detail e.g. for qualitative interpretative phenomenological analysis might be considered	1. Consideration of some the research stakeholders e.g. use of pilot study with target sample but no stakeholder involvement in planning stages of study design.	3. Thorough discussion of strengths and limitations of all aspects of study including design, methods, data collection tools, sample & analytic approach.
Hamadneh, S., Kassab, M., Hamadneh, J., & Amarin, Z. (2016). Sudden unexpected infant death in Jordan and the home environment [Article]. Pediatrics International, 58(12), 1333-1336. https://doi.org/10.1111/ped.13016	1. General reference to broad theories or concepts that frame the study. e.g. key concepts were identified in the introduction section.	2. Aims statement made but may only appear in the abstract or be lacking detail.	1. General description of research area but not of the specific research environment e.g. 'in primary care.'	2. The study design can address the stated research aim/s but there is a more suitable alternative that could have been	0. No mention of the sampling approach.	1. Very limited explanation for choice of data collection tool/s. e.g. based on availability of tool.	2. Structure and/or content of tool/s allow for data to be gathered broadly addressing the stated aim/s but could benefit from	1. Basic and brief outline of data collection procedure e.g. 'using a questionnaire distributed to staff'.	2. Some recruitment data but not a complete account e.g. number of people who were invited and agreed.	1. Very limited justification for choice of analytic method selected. e.g. previous use by the research team.	3. Method of analysis selected is the most suitable approach to attempt answer the research aim/s in detail e.g. for qualitative interpretative phenomenological analysis might be considered	2. Evidence of stakeholder input informing the research. e.g. use of pilot study with feedback influencing the study design/conduct or reference to a project reference	0. No mention at all
Hauck, F. R., Tanabe, K. O., McMurry, T., & Moon, R. Y. (2015). Evaluation of Bedtime Basics for Babies: A National Crib Distribution Program to Reduce the Risk of Sleep-Related Sudden Infant Deaths [Article]. Journal of Community Health, 40(3), 457-463. https://doi.org/10.1007/s10900-014-9957-0	3. Explicit discussion of the theories or concepts that inform the study, with application of the theory or concept evident through the design,	3. Explicit and detailed statement of aims/objectives in main body of report.	3. Specific description of the research setting and target population of study e.g. 'nurses and doctors from GP practices in	3. The study design selected appears to be the most suitable approach to attempt to answer the stated research aim/s.	1. Evidence of consideration of the sample required e.g. the sample characteristics are described and appear appropriate to address the	3. Detailed explanation of rationale for choice of data collection tool/s. e.g. relevance to the study aim/s, co-designed with the	3. Structure and content of tool/s allow for detailed data to be gathered around all relevant issues required to address the	3. Detailed description of each stage of the data collection procedure, including when, where and how data was gathered such that the	3. Complete data allowing for full picture of recruitment outcomes e.g. number of people approached, recruited, and who	3. Detailed justification for choice of analytic method selected e.g. relevance to the study aim/s or comment around of the strengths	3. Method of analysis selected is the most suitable approach to attempt answer the research aim/s in detail e.g. for qualitative interpretative phenomenological analysis might be considered	1. Consideration of some the research stakeholders e.g. use of pilot study with target sample but no stakeholder involvement in planning stages of study design.	3. Thorough discussion of strengths and limitations of all aspects of study including design, methods, data collection tools, sample & analytic approach.
Hauck, F. R., Moon, R. Y., Kerr, S. M., Corwln, M. J., Heeren, T., Colson, E., Parker, M. G., & Kellams, A. (2024). Mothers Falling Asleep During Infant Feeding.	3. Explicit discussion of the theories or concepts that inform the study, with application of the theory or concept evident through the design,	3. Explicit and detailed statement of aims/objectives in main body of report.	3. Specific description of the research setting and target population of study e.g. 'nurses and doctors from GP practices in	3. The study design selected appears to be the most suitable approach to attempt to answer the stated research aim/s.	3. Detailed evidence of consideration of the sample required to address the research aim/s. e.g. sample size calculation or discussion of an iterative	3. Detailed explanation of rationale for choice of data collection tool/s. e.g. relevance to the study aim/s, co-designed with the	3. Structure and content of tool/s allow for detailed data to be gathered around all relevant issues required to address the	3. Detailed description of each stage of the data collection procedure, including when, where and how data was gathered such that the	3. Complete data allowing for full picture of recruitment outcomes e.g. number of people approached, recruited, and who	3. Detailed justification for choice of analytic method selected e.g. relevance to the study aim/s or comment around of the strengths	3. Method of analysis selected is the most suitable approach to attempt answer the research aim/s in detail e.g. for qualitative interpretative phenomenological analysis might be considered	2. Evidence of stakeholder input informing the research. e.g. use of pilot study with feedback influencing the study design/conduct or reference to a project reference	3. Thorough discussion of strengths and limitations of all aspects of study including design, methods, data collection tools, sample & analytic approach.

Herman, S., Adkins, M., & Moon, R. (2015). Knowledge and Beliefs of African-American and American Indian Parents and Supporters About Infant Safe Sleep. Journal of Community Health, 40(1), 12-19. https://doi.org/10.1007/s10900-014-9886-y	3. Explicit discussion of the theories or concepts that inform the study, with application of the theory or concept evident through the design,	3. Explicit and detailed statement of aims/objectives in main body of report.	3. Specific description of the research setting and target population of study e.g. 'nurses and doctors from GP practices in	3. The study design selected appears to be the most suitable approach to attempt to answer the stated research aim/s.	2. Evidence of consideration of sample required to address the aim. e.g. the sample characteristics are described with	3. Detailed explanation of rationale for choice of data collection tool/s. e.g. relevance to the study aim/s, co-designed with the	3. Structure and content of tool/s allow for detailed data to be gathered around all relevant issues required to address the	3. Detailed description of each stage of the data collection procedure, including when, where and how data was gathered such that the	2. Some recruitment data but not a complete account e.g. number of people who were invited and agreed.	3. Detailed justification for choice of analytic method selected e.g. relevance to the study aim/s or comment around of the strengths	3. Method of analysis selected is the most suitable approach to attempt answer the research aim/s in detail e.g. for qualitative interpretative phenomenological analysis might be considered	0. No mention at all	3. Thorough discussion of strengths and limitations of all aspects of study including design, methods, data collection tools, sample & analytic approach.
Hirsch, H. M., Mullins, S. H., Miller, B. K., & Aitken, M. E. (2018). Paternal perception of infant sleep risks and safety. Inj Epidemiol, 5(Suppl 1), 9. https://doi.org/10.1186/s40621-018-0140-4	3. Explicit discussion of the theories or concepts that inform the study, with application of the theory or concept evident through the design,	3. Explicit and detailed statement of aims/objectives in main body of report.	1. General description of research area but not of the specific research environment e.g. 'in primary care.'	3. The study design selected appears to be the most suitable approach to attempt to answer the stated research aim/s.	1. Evidence of consideration of the sample required e.g. the sample characteristics are described and appear appropriate to address the	3. Detailed explanation of rationale for choice of data collection tool/s. e.g. relevance to the study aim/s, co-designed with the	3. Structure and content of tool/s allow for detailed data to be gathered around all relevant issues required to address the	3. Detailed description of each stage of the data collection procedure, including when, where and how data was gathered such that the	1. Minimal and basic recruitment data e.g. number of people invited who agreed to take part.	3. Detailed justification for choice of analytic method selected e.g. relevance to the study aim/s or comment around of the strengths	3. Method of analysis selected is the most suitable approach to attempt answer the research aim/s in detail e.g. for qualitative interpretative phenomenological analysis might be considered	0. No mention at all	3. Thorough discussion of strengths and limitations of all aspects of study including design, methods, data collection tools, sample & analytic approach.
Huber, R., Menon, M., Russell, R. B., Smith, S., Scott, S., & Berns, S. D. (2024). Community infant safe sleep and breastfeeding promotion and population level-outcomes: A mixed methods study. Midwifery, 132, 103953. https://doi.org/10.1016/j.midw.2024.103953	3. Explicit discussion of the theories or concepts that inform the study, with application of the theory or concept evident through the design,	3. Explicit and detailed statement of aims/objectives in main body of report.	2. Description of research setting is made but is lacking detail e.g. 'in primary care practices in region [x]'.	2. The study design can address the stated research aim/s but there is a more suitable alternative that could have been	0. No mention of the sampling approach.	3. Detailed explanation of rationale for choice of data collection tool/s. e.g. relevance to the study aim/s, co-designed with the	2. Structure and/or content of tool/s allow for data to be gathered broadly addressing the stated aim/s but could benefit from	3. Detailed description of each stage of the data collection procedure, including when, where and how data was gathered such that the	1. Minimal and basic recruitment data e.g. number of people invited who agreed to take part.	3. Detailed justification for choice of analytic method selected e.g. relevance to the study aim/s or comment around of the strengths	3. Method of analysis selected is the most suitable approach to attempt answer the research aim/s in detail e.g. for qualitative interpretative phenomenological analysis might be considered	0. No mention at all	2. Discussion of some of the key strengths and weaknesses of the study but not complete. e.g. several strengths/limitations explored but with notable omissions or lack of depth of explanation.
Hutchison, B. L., Thompson, J. M. D., & Mitchell, E. A. (2015). Infant care practices related to sudden unexpected death in infancy: A 2013 survey [Article]. New Zealand Medical Journal, 128(1408), 15-22. https://www.scopus.com/inward/record.uri?eid=2-s2.0-84930278588&partnerID=40&md5=ea9ad6099644478180390f3810cea282	2. Identification of specific theories or concepts that frame the study and how these informed the work undertaken. e.g. key	3. Explicit and detailed statement of aims/objectives in main body of report.	2. Description of research setting is made but is lacking detail e.g. 'in primary care practices in region [x]'.	3. The study design selected appears to be the most suitable approach to attempt to answer the stated research aim/s.	1. Evidence of consideration of the sample required e.g. the sample characteristics are described and appear appropriate to address the	2. Basic explanation of rationale for choice of data collection tool/s. e.g. based on use in a prior similar study.	3. Structure and content of tool/s allow for detailed data to be gathered around all relevant issues required to address the	3. Detailed description of each stage of the data collection procedure, including when, where and how data was gathered such that the	3. Complete data allowing for full picture of recruitment outcomes e.g. number of people approached, recruited, and who	2. Basic justification for choice of analytic method selected e.g. method used in prior similar research.	3. Method of analysis selected is the most suitable approach to attempt answer the research aim/s in detail e.g. for qualitative interpretative phenomenological analysis might be considered	0. No mention at all	3. Thorough discussion of strengths and limitations of all aspects of study including design, methods, data collection tools, sample & analytic approach.
Hwang, S., Rybin, D., Heeren, T., Colson, E., & Corwin, M. (2016). Trust in Sources of Advice about Infant Care Practices: The SAFE Study. Maternal & Child Health Journal, 20(9), 1956-1964. https://doi.org/10.1007/s10995-016-2011-3	2. Identification of specific theories or concepts that frame the study and how these informed the work undertaken. e.g. key	3. Explicit and detailed statement of aims/objectives in main body of report.	3. Specific description of the research setting and target population of study e.g. 'nurses and doctors from GP practices in	3. The study design selected appears to be the most suitable approach to attempt to answer the stated research aim/s.	3. Detailed evidence of consideration of the sample required to address the research aim/s. e.g. sample size calculation or discussion of an iterative	3. Detailed explanation of rationale for choice of data collection tool/s. e.g. relevance to the study aim/s, co-designed with the	3. Structure and content of tool/s allow for detailed data to be gathered around all relevant issues required to address the	3. Detailed description of each stage of the data collection procedure, including when, where and how data was gathered such that the	3. Complete data allowing for full picture of recruitment outcomes e.g. number of people approached, recruited, and who	3. Detailed justification for choice of analytic method selected e.g. relevance to the study aim/s or comment around of the strengths	3. Method of analysis selected is the most suitable approach to attempt answer the research aim/s in detail e.g. for qualitative interpretative phenomenological analysis might be considered	1. Consideration of some the research stakeholders e.g. use of pilot study with target sample but no stakeholder involvement in planning stages of study design.	2. Discussion of some of the key strengths and weaknesses of the study but not complete. e.g. several strengths/limitations explored but with notable omissions or lack of depth of explanation.
Hwang, S. S., Parker, M. G., Colvin, B. N., Forbes, E. S., Brown, K., & Colson, E. R. (2021). Understanding the barriers and facilitators to safe infant sleep for mothers of preterm infants [Article]. Journal of Perinatology, 41(8), 1992-1999. https://doi.org/10.1038/s41372-020-00896-5	3. Explicit discussion of the theories or concepts that inform the study, with application of the theory or concept evident through the design,	3. Explicit and detailed statement of aims/objectives in main body of report.	3. Specific description of the research setting and target population of study e.g. 'nurses and doctors from GP practices in	3. The study design selected appears to be the most suitable approach to attempt to answer the stated research aim/s.	3. Detailed evidence of consideration of the sample required to address the research aim/s. e.g. sample size calculation or discussion of an iterative	3. Detailed explanation of rationale for choice of data collection tool/s. e.g. relevance to the study aim/s, co-designed with the	3. Structure and content of tool/s allow for detailed data to be gathered around all relevant issues required to address the	3. Detailed description of each stage of the data collection procedure, including when, where and how data was gathered such that the	1. Minimal and basic recruitment data e.g. number of people invited who agreed to take part.	3. Detailed justification for choice of analytic method selected e.g. relevance to the study aim/s or comment around of the strengths	3. Method of analysis selected is the most suitable approach to attempt answer the research aim/s in detail e.g. for qualitative interpretative phenomenological analysis might be considered	0. No mention at all	3. Thorough discussion of strengths and limitations of all aspects of study including design, methods, data collection tools, sample & analytic approach.

Kadakia, A., Joyner, B., Tender, J., Oden, R., & Moon, R. Y. (2015). Breastfeeding in African Americans May Not Depend on Sleep Arrangement. Clinical Pediatrics, 54(1), 47-53. https://doi.org/10.1177/0009922814547565	1. General reference to broad theories or concepts that frame the study. e.g. key concepts were identified in the introduction section.	3. Explicit and detailed statement of aims/objectives in main body of report.	3. Specific description of the research setting and target population of study e.g. 'nurses and doctors from GP practices in	3. The study design selected appears to be the most suitable approach to attempt to answer the stated research aim/s.	3. Detailed evidence of consideration of the sample required to address the research aim/s. e.g. sample size calculation or discussion of an iterative	3. Detailed explanation of rationale for choice of data collection tool/s. e.g. relevance to the study aim/s, co-designed with the	3. Structure and content of tool/s allow for detailed data to be gathered around all relevant issues required to address the	3. Detailed description of each stage of the data collection procedure, including when, where and how data was gathered such that the	3. Complete data allowing for full picture of recruitment outcomes e.g. number of people approached, recruited, and who	3. Detailed justification for choice of analytic method selected e.g. relevance to the study aim/s or comment around of the strengths	3. Method of analysis selected is the most suitable approach to attempt answer the research aim/s in detail e.g. for qualitative interpretative phenomenological analysis might be considered	0. No mention at all	3. Thorough discussion of strengths and limitations of all aspects of study including design, methods, data collection tools, sample & analytic approach.
Lerner, R. E., Camerota, M., Tully, K. P., & Propper, C. (2020). Associations between mother-infant bed-sharing practices and infant affect and behavior during the still-face paradigm. Infant Behavior & Development, 60, N.PAG-N.PAG. https://doi.org/10.1016/j.infbeh.2020.101464	3. Explicit discussion of the theories or concepts that inform the study, with application of the theory or concept evident through the design,	3. Explicit and detailed statement of aims/objectives in main body of report.	3. Specific description of the research setting and target population of study e.g. 'nurses and doctors from GP practices in	3. The study design selected appears to be the most suitable approach to attempt to answer the stated research aim/s.	2. Evidence of consideration of sample required to address the aim. e.g. the sample characteristics are described with	3. Detailed explanation of rationale for choice of data collection tool/s. e.g. relevance to the study aim/s, co-designed with the	3. Structure and content of tool/s allow for detailed data to be gathered around all relevant issues required to address the	3. Detailed description of each stage of the data collection procedure, including when, where and how data was gathered such that the	3. Complete data allowing for full picture of recruitment outcomes e.g. number of people approached, recruited, and who	3. Detailed justification for choice of analytic method selected e.g. relevance to the study aim/s or comment around of the strengths	3. Method of analysis selected is the most suitable approach to attempt answer the research aim/s in detail e.g. for qualitative interpretative phenomenological analysis might be considered	0. No mention at all	3. Thorough discussion of strengths and limitations of all aspects of study including design, methods, data collection tools, sample & analytic approach.
Louis-Jacques, A. F., Bartick, M., Awomolo, A., Zhang, J., Feldman-Winter, L., Leonard, S. A., Meek, J., Mitchell, K. B., & Crowe, S. (2024). Bedsharing among breastfeeding physicians: Results of a nationwide survey.	3. Explicit discussion of the theories or concepts that inform the study, with application of the theory or concept evident through the design,	3. Explicit and detailed statement of aims/objectives in main body of report.	3. Specific description of the research setting and target population of study e.g. 'nurses and doctors from GP practices in	2. The study design can address the stated research aim/s but there is a more suitable alternative that could have been	3. Detailed evidence of consideration of the sample required to address the research aim/s. e.g. sample size calculation or discussion of an iterative	2. Basic explanation of rationale for choice of data collection tool/s. e.g. based on use in a prior similar study.	2. Structure and/or content of tool/s allow for data to be gathered broadly addressing the stated aim/s but could benefit from	3. Detailed description of each stage of the data collection procedure, including when, where and how data was gathered such that the	3. Complete data allowing for full picture of recruitment outcomes e.g. number of people approached, recruited, and who	3. Detailed justification for choice of analytic method selected e.g. relevance to the study aim/s or comment around of the strengths	3. Method of analysis selected is the most suitable approach to attempt answer the research aim/s in detail e.g. for qualitative interpretative phenomenological analysis might be considered	1. Consideration of some the research stakeholders e.g. use of pilot study with target sample but no stakeholder involvement in planning stages of study design.	3. Thorough discussion of strengths and limitations of all aspects of study including design, methods, data collection tools, sample & analytic approach.
Luijk, M. P. C. M., Mileva-Seitz, V. R., Jansen, P. W., van Ijzendoorn, M. H., Jaddoe, V. W. V., Raat, H., Hofman, A., Verhulst, F. C., & Tiemeier, H. (2013). Ethnic differences in prevalence and determinants of mother–child bed-sharing in early childhood. Sleep Medicine, 14(11), 1092-1099. https://doi.org/https://doi.org/10.1016/j.sleep.2013.04.019	2. Identification of specific theories or concepts that frame the study and how these informed the work undertaken. e.g. key	3. Explicit and detailed statement of aims/objectives in main body of report.	3. Specific description of the research setting and target population of study e.g. 'nurses and doctors from GP practices in	3. The study design selected appears to be the most suitable approach to attempt to answer the stated research aim/s.	3. Detailed evidence of consideration of the sample required to address the research aim/s. e.g. sample size calculation or discussion of an iterative	3. Detailed explanation of rationale for choice of data collection tool/s. e.g. relevance to the study aim/s, co-designed with the	3. Structure and content of tool/s allow for detailed data to be gathered around all relevant issues required to address the	3. Detailed description of each stage of the data collection procedure, including when, where and how data was gathered such that the	3. Complete data allowing for full picture of recruitment outcomes e.g. number of people approached, recruited, and who	3. Detailed justification for choice of analytic method selected e.g. relevance to the study aim/s or comment around of the strengths	3. Method of analysis selected is the most suitable approach to attempt answer the research aim/s in detail e.g. for qualitative interpretative phenomenological analysis might be considered	0. No mention at all	2. Discussion of some of the key strengths and weaknesses of the study but not complete. e.g. several strengths/limitations explored but with notable omissions or lack of depth of explanation.
MacFarlane, M., Thompson, J. M. D., Mitchell, E. A., Lawton, B., McLardy, E. M., Jonas, S. D., Tepania-Palmer, G., Roa, T., Warren, G., & Jowsey, T. (2021). Pēpē-infant sleep practices and sudden unexpected death in infancy in Aotearoa New Zealand [Article]. International Journal of Gynecology and Obstetrics, 155(2), 305-317. https://doi.org/10.1002/ijgo.13910	3. Explicit discussion of the theories or concepts that inform the study, with application of the theory or concept evident through the design,	3. Explicit and detailed statement of aims/objectives in main body of report.	3. Specific description of the research setting and target population of study e.g. 'nurses and doctors from GP practices in	3. The study design selected appears to be the most suitable approach to attempt to answer the stated research aim/s.	3. Detailed evidence of consideration of the sample required to address the research aim/s. e.g. sample size calculation or discussion of an iterative	3. Detailed explanation of rationale for choice of data collection tool/s. e.g. relevance to the study aim/s, co-designed with the	3. Structure and content of tool/s allow for detailed data to be gathered around all relevant issues required to address the	3. Detailed description of each stage of the data collection procedure, including when, where and how data was gathered such that the	3. Complete data allowing for full picture of recruitment outcomes e.g. number of people approached, recruited, and who	3. Detailed justification for choice of analytic method selected e.g. relevance to the study aim/s or comment around of the strengths	3. Method of analysis selected is the most suitable approach to attempt answer the research aim/s in detail e.g. for qualitative interpretative phenomenological analysis might be considered	2. Evidence of stakeholder input informing the research. e.g. use of pilot study with feedback influencing the study design/conduct or reference to a project reference	3. Thorough discussion of strengths and limitations of all aspects of study including design, methods, data collection tools, sample & analytic approach.
Mathews, A., Joyner, B., Oden, R., Alamo, I., & Moon, R. (2015). Comparison of Infant Sleep Practices in African-American and US Hispanic Families: Implications for Sleep-Related Infant Death. Journal of Immigrant & Minority Health, 17(3), 834-842. https://doi.org/10.1007/s10903-014-0016-9	2. Identification of specific theories or concepts that frame the study and how these informed the work undertaken. e.g. key	3. Explicit and detailed statement of aims/objectives in main body of report.	3. Specific description of the research setting and target population of study e.g. 'nurses and doctors from GP practices in	3. The study design selected appears to be the most suitable approach to attempt to answer the stated research aim/s.	3. Detailed evidence of consideration of the sample required to address the research aim/s. e.g. sample size calculation or discussion of an iterative	3. Detailed explanation of rationale for choice of data collection tool/s. e.g. relevance to the study aim/s, co-designed with the	3. Structure and content of tool/s allow for detailed data to be gathered around all relevant issues required to address the	3. Detailed description of each stage of the data collection procedure, including when, where and how data was gathered such that the	2. Some recruitment data but not a complete account e.g. number of people who were invited and agreed.	3. Detailed justification for choice of analytic method selected e.g. relevance to the study aim/s or comment around of the strengths	3. Method of analysis selected is the most suitable approach to attempt answer the research aim/s in detail e.g. for qualitative interpretative phenomenological analysis might be considered	0. No mention at all	3. Thorough discussion of strengths and limitations of all aspects of study including design, methods, data collection tools, sample & analytic approach.

McIntosh, C., Trenholme, A., Stewart, J., & Vogel, A. (2018). Evaluation of a sudden unexpected death in infancy intervention programme aimed at improving parental awareness of risk factors and protective infant care practices. J Paediatr Child Health, 54(4), 377-382. https://doi.org/10.1111/jpc.13772	3. Explicit discussion of the theories or concepts that inform the study, with application of the theory or concept evident through the design,	3. Explicit and detailed statement of aims/objectives in main body of report.	3. Specific description of the research setting and target population of study e.g. ‘nurses and doctors from GP practices in	3. The study design selected appears to be the most suitable approach to attempt to answer the stated research aim/s.	1. Evidence of consideration of the sample required e.g. the sample characteristics are described and appear appropriate to address the	3. Detailed explanation of rationale for choice of data collection tool/s. e.g. relevance to the study aim/s, co-designed with the	3. Structure and content of tool/s allow for detailed data to be gathered around all relevant issues required to address the	3. Detailed description of each stage of the data collection procedure, including when, where and how data was gathered such that the	3. Complete data allowing for full picture of recruitment outcomes e.g. number of people approached, recruited, and who	3. Detailed justification for choice of analytic method selected e.g. relevance to the study aim/s or comment around of the strengths	3. Method of analysis selected is the most suitable approach to attempt answer the research aim/s in detail e.g. for qualitative interpretative phenomenological analysis might be considered	0. No mention at all	3. Thorough discussion of strengths and limitations of all aspects of study including design, methods, data collection tools, sample & analytic approach.
Moon, R. Y., Mindell, J. A., Honaker, S., Keim, S., Roberts, K. J., McAdams, R. J., & McKenzie, L. B. (2024). The Tension Between AAP Safe Sleep Guidelines and Infant Sleep. Pediatrics (Evanston), 153(4), 1. https://doi.org/10.1542/peds.2023-064675	2. Identification of specific theories or concepts that frame the study and how these informed the work undertaken. e.g. key	3. Explicit and detailed statement of aims/objectives in main body of report.	3. Specific description of the research setting and target population of study e.g. ‘nurses and doctors from GP practices in	3. The study design selected appears to be the most suitable approach to attempt to answer the stated research aim/s.	3. Detailed evidence of consideration of the sample required to address the research aim/s. e.g. sample size calculation or discussion of an iterative	3. Detailed explanation of rationale for choice of data collection tool/s. e.g. relevance to the study aim/s, co-designed with the	3. Structure and content of tool/s allow for detailed data to be gathered around all relevant issues required to address the	3. Detailed description of each stage of the data collection procedure, including when, where and how data was gathered such that the	3. Complete data allowing for full picture of recruitment outcomes e.g. number of people approached, recruited, and who	2. Basic justification for choice of analytic method selected e.g. method used in prior similar research.	2. Method of analysis can address the research aim/s but there is a more suitable alternative that could have been used or used in addition to offer a stronger analysis.	0. No mention at all	2. Discussion of some of the key strengths and weaknesses of the study but not complete. e.g. several strengths/limitations explored but with notable omissions or lack of depth of explanation.
Morrison, T. M., Standish, K. R., Wanar, A., Crowell, L., Safon, C. B., Colvin, B. N., Friedman, H., Schiff, D. M., Wachman, E. M., Colson, E. R., Drainoni, M. L., & Parker, M. G. (2023). Drivers of decision-making regarding infant sleep practices among mothers with opioid use disorder [Article]. Journal of Perinatology, 43(7), 923-929. https://doi.org/10.1038/s41372-023-01701-9	3. Explicit discussion of the theories or concepts that inform the study, with application of the theory or concept evident through the design,	3. Explicit and detailed statement of aims/objectives in main body of report.	3. Specific description of the research setting and target population of study e.g. ‘nurses and doctors from GP practices in	3. The study design selected appears to be the most suitable approach to attempt to answer the stated research aim/s.	3. Detailed evidence of consideration of the sample required to address the research aim/s. e.g. sample size calculation or discussion of an iterative	3. Detailed explanation of rationale for choice of data collection tool/s. e.g. relevance to the study aim/s, co-designed with the	3. Structure and content of tool/s allow for detailed data to be gathered around all relevant issues required to address the	3. Detailed description of each stage of the data collection procedure, including when, where and how data was gathered such that the	2. Some recruitment data but not a complete account e.g. number of people who were invited and agreed.	3. Detailed justification for choice of analytic method selected e.g. relevance to the study aim/s or comment around of the strengths	3. Method of analysis selected is the most suitable approach to attempt answer the research aim/s in detail e.g. for qualitative interpretative phenomenological analysis might be considered	0. No mention at all	3. Thorough discussion of strengths and limitations of all aspects of study including design, methods, data collection tools, sample & analytic approach.
Murray, L., Tran, T., Thang, V. V., Cass, L., & Fisher, J. (2018). How do caregivers understand and respond to unsettled infant behaviour in Vietnam? A qualitative study. Child Care Health Dev, 44(1), 62-70. https://doi.org/10.1111/cch.12474	2. Identification of specific theories or concepts that frame the study and how these informed the work undertaken. e.g. key	3. Explicit and detailed statement of aims/objectives in main body of report.	3. Specific description of the research setting and target population of study e.g. ‘nurses and doctors from GP practices in	3. The study design selected appears to be the most suitable approach to attempt to answer the stated research aim/s.	3. Detailed evidence of consideration of the sample required to address the research aim/s. e.g. sample size calculation or discussion of an iterative	3. Detailed explanation of rationale for choice of data collection tool/s. e.g. relevance to the study aim/s, co-designed with the	3. Structure and content of tool/s allow for detailed data to be gathered around all relevant issues required to address the	3. Detailed description of each stage of the data collection procedure, including when, where and how data was gathered such that the	3. Complete data allowing for full picture of recruitment outcomes e.g. number of people approached, recruited, and who	3. Detailed justification for choice of analytic method selected e.g. relevance to the study aim/s or comment around of the strengths	3. Method of analysis selected is the most suitable approach to attempt answer the research aim/s in detail e.g. for qualitative interpretative phenomenological analysis might be considered	1. Consideration of some the research stakeholders e.g. use of pilot study with target sample but no stakeholder involvement in planning stages of study design.	3. Thorough discussion of strengths and limitations of all aspects of study including design, methods, data collection tools, sample & analytic approach.
Osei-Poku, G. K., Mwananyanda, L., Elliot, P. A., MacLeod, W. B., Somwe, S. W., Pieciak, R. C., & Gill, C. J. (2022). Assessing infant sleep practices and other risk factors of SIDS in Zambia: a cross-sectional survey of mothers in Lusaka, Zambia. BMC Pediatrics, 22, 1-9. https://doi.org/10.1186/s12887-022-03712-5	3. Explicit discussion of the theories or concepts that inform the study, with application of the theory or concept evident through the design,	3. Explicit and detailed statement of aims/objectives in main body of report.	3. Specific description of the research setting and target population of study e.g. ‘nurses and doctors from GP practices in	3. The study design selected appears to be the most suitable approach to attempt to answer the stated research aim/s.	3. Detailed evidence of consideration of the sample required to address the research aim/s. e.g. sample size calculation or discussion of an iterative	3. Detailed explanation of rationale for choice of data collection tool/s. e.g. relevance to the study aim/s, co-designed with the	3. Structure and content of tool/s allow for detailed data to be gathered around all relevant issues required to address the	3. Detailed description of each stage of the data collection procedure, including when, where and how data was gathered such that the	3. Complete data allowing for full picture of recruitment outcomes e.g. number of people approached, recruited, and who	3. Detailed justification for choice of analytic method selected e.g. relevance to the study aim/s or comment around of the strengths	3. Method of analysis selected is the most suitable approach to attempt answer the research aim/s in detail e.g. for qualitative interpretative phenomenological analysis might be considered	1. Consideration of some the research stakeholders e.g. use of pilot study with target sample but no stakeholder involvement in planning stages of study design.	3. Thorough discussion of strengths and limitations of all aspects of study including design, methods, data collection tools, sample & analytic approach.
Osei-Poku, G. K., Mwananyanda, L., Elliott, P. A., MacLeod, W. B., Somwe, S. W., Pieciak, R. C., Hamapa, A., & Gill, C. J. (2023). Qualitative assessment of infant sleep practices and other risk factors of sudden infant death syndrome (SIDS) among mothers in Lusaka, Zambia. BMC Pediatrics, 23(1), 245-245. https://doi.org/10.1186/s12887-023-04051-9	3. Explicit discussion of the theories or concepts that inform the study, with application of the theory or concept evident through the design,	3. Explicit and detailed statement of aims/objectives in main body of report.	3. Specific description of the research setting and target population of study e.g. ‘nurses and doctors from GP practices in	3. The study design selected appears to be the most suitable approach to attempt to answer the stated research aim/s.	3. Detailed evidence of consideration of the sample required to address the research aim/s. e.g. sample size calculation or discussion of an iterative	3. Detailed explanation of rationale for choice of data collection tool/s. e.g. relevance to the study aim/s, co-designed with the	3. Structure and content of tool/s allow for detailed data to be gathered around all relevant issues required to address the	3. Detailed description of each stage of the data collection procedure, including when, where and how data was gathered such that the	3. Complete data allowing for full picture of recruitment outcomes e.g. number of people approached, recruited, and who	3. Detailed justification for choice of analytic method selected e.g. relevance to the study aim/s or comment around of the strengths	3. Method of analysis selected is the most suitable approach to attempt answer the research aim/s in detail e.g. for qualitative interpretative phenomenological analysis might be considered	0. No mention at all	3. Thorough discussion of strengths and limitations of all aspects of study including design, methods, data collection tools, sample & analytic approach.

Pease, A., Ingram, J., Blair, P. S., & Fleming, P. J. (2017). Factors influencing maternal decision-making for the infant sleep environment in families at higher risk of SIDS: a qualitative study. BMJ Paediatr Open, 1(1), e000133. https://doi.org/10.1136/bmjpo-2017-000133	3. Explicit discussion of the theories or concepts that inform the study, with application of the theory or concept evident through the design,	3. Explicit and detailed statement of aims/objectives in main body of report.	3. Specific description of the research setting and target population of study e.g. 'nurses and doctors from GP practices in	3. The study design selected appears to be the most suitable approach to attempt to answer the stated research aim/s.	3. Detailed evidence of consideration of the sample required to address the research aim/s. e.g. sample size calculation or discussion of an iterative	3. Detailed explanation of rationale for choice of data collection tool/s. e.g. relevance to the study aim/s, co-designed with the	3. Structure and content of tool/s allow for detailed data to be gathered around all relevant issues required to address the	3. Detailed description of each stage of the data collection procedure, including when, where and how data was gathered such that the	3. Complete data allowing for full picture of recruitment outcomes e.g. number of people approached, recruited, and who	3. Detailed justification for choice of analytic method selected e.g. relevance to the study aim/s or comment around of the strengths	3. Method of analysis selected is the most suitable approach to attempt answer the research aim/s in detail e.g. for qualitative interpretative phenomenological analysis might be considered	0. No mention at all	3. Thorough discussion of strengths and limitations of all aspects of study including design, methods, data collection tools, sample & analytic approach.
Pease, A., Turner, N., Ingram, J., Fleming, P., Patrick, K., Williams, T., Sleap, V., Pitts, K., Luyt, K., Ali, B., & Blair, P. (2023). Changes in background characteristics and risk factors among SIDS infants in England: Cohort comparisons from 1993 to 2020 [Article]. BMJ Open, 13(10), Article e076751. https://doi.org/10.1136/bmjopen-2023-076751	3. Explicit discussion of the theories or concepts that inform the study, with application of the theory or concept evident through the design,	3. Explicit and detailed statement of aims/objectives in main body of report.	3. Specific description of the research setting and target population of study e.g. 'nurses and doctors from GP practices in	3. The study design selected appears to be the most suitable approach to attempt to answer the stated research aim/s.	3. Detailed evidence of consideration of the sample required to address the research aim/s. e.g. sample size calculation or discussion of an iterative	3. Detailed explanation of rationale for choice of data collection tool/s. e.g. relevance to the study aim/s, co-designed with the	3. Structure and content of tool/s allow for detailed data to be gathered around all relevant issues required to address the	3. Detailed description of each stage of the data collection procedure, including when, where and how data was gathered such that the	3. Complete data allowing for full picture of recruitment outcomes e.g. number of people approached, recruited, and who	3. Detailed justification for choice of analytic method selected e.g. relevance to the study aim/s or comment around of the strengths	3. Method of analysis selected is the most suitable approach to attempt answer the research aim/s in detail e.g. for qualitative interpretative phenomenological analysis might be considered	3. Substantial consultation with stakeholders identifiable in planning of study design and in preliminary work e.g. consultation in the conceptualisatio	3. Thorough discussion of strengths and limitations of all aspects of study including design, methods, data collection tools, sample & analytic approach.
Pretorius, K., Choi, E., Kang, S., & Mackert, M. (2020). Sudden infant death syndrome on Facebook: Qualitative descriptive content analysis to guide prevention efforts [Article]. Journal of Medical Internet Research, 22(7), Article e18474. https://doi.org/10.2196/18474	2. Identification of specific theories or concepts that frame the study and how these informed the work undertaken. e.g. key	3. Explicit and detailed statement of aims/objectives in main body of report.	1. General description of research area but not of the specific research environment e.g. 'in primary care.'	3. The study design selected appears to be the most suitable approach to attempt to answer the stated research aim/s.	1. Evidence of consideration of the sample required e.g. the sample characteristics are described and appear appropriate to address the	2. Basic explanation of rationale for choice of data collection tool/s. e.g. based on use in a prior similar study.	2. Structure and/or content of tool/s allow for data to be gathered broadly addressing the stated aim/s but could benefit from	3. Detailed description of each stage of the data collection procedure, including when, where and how data was gathered such that the	3. Complete data allowing for full picture of recruitment outcomes e.g. number of people approached, recruited, and who	2. Basic justification for choice of analytic method selected e.g. method used in prior similar research.	3. Method of analysis selected is the most suitable approach to attempt answer the research aim/s in detail e.g. for qualitative interpretative phenomenological analysis might be considered	0. No mention at all	2. Discussion of some of the key strengths and weaknesses of the study but not complete. e.g. several strengths/limitations explored but with notable omissions or lack of depth of explanation.
Rudzik, A., & Ball, H. (2016). Exploring Maternal Perceptions of Infant Sleep and Feeding Method Among Mothers in the United Kingdom: A Qualitative Focus Group Study. Maternal & Child Health Journal, 20(1), 33-40. https://doi.org/10.1007/s10995-015-1798-7	3. Explicit discussion of the theories or concepts that inform the study, with application of the theory or concept evident through the design,	3. Explicit and detailed statement of aims/objectives in main body of report.	3. Specific description of the research setting and target population of study e.g. 'nurses and doctors from GP practices in	3. The study design selected appears to be the most suitable approach to attempt to answer the stated research aim/s.	0. No mention of the sampling approach.	1. Very limited explanation for choice of data collection tool/s. e.g. based on availability of tool.	3. Structure and content of tool/s allow for detailed data to be gathered around all relevant issues required to address the	2. States each stage of data collection procedure but with limited detail or states some stages in detail but omits others	0. No mention of recruitment data.	0. No mention of the rationale for the analytic method chosen.	3. Method of analysis selected is the most suitable approach to attempt answer the research aim/s in detail e.g. for qualitative interpretative phenomenological analysis might be considered	0. No mention at all	3. Thorough discussion of strengths and limitations of all aspects of study including design, methods, data collection tools, sample & analytic approach.
Sahud, H., Berger, R. P., Hamm, M., Heineman, E., Cameron, F., Wasilewski, J., Griffin, A., & Muniz, G. B. (2025). Understanding parental choices related to infant sleep practices in the United States using a mixed methods approach.	3. Explicit discussion of the theories or concepts that inform the study, with application of the theory or concept evident through the design,	3. Explicit and detailed statement of aims/objectives in main body of report.	3. Specific description of the research setting and target population of study e.g. 'nurses and doctors from GP practices in	3. The study design selected appears to be the most suitable approach to attempt to answer the stated research aim/s.	3. Detailed evidence of consideration of the sample required to address the research aim/s. e.g. sample size calculation or discussion of an iterative	3. Detailed explanation of rationale for choice of data collection tool/s. e.g. relevance to the study aim/s, co-designed with the	3. Structure and content of tool/s allow for detailed data to be gathered around all relevant issues required to address the	3. Detailed description of each stage of the data collection procedure, including when, where and how data was gathered such that the	3. Complete data allowing for full picture of recruitment outcomes e.g. number of people approached, recruited, and who	3. Detailed justification for choice of analytic method selected e.g. relevance to the study aim/s or comment around of the strengths	3. Method of analysis selected is the most suitable approach to attempt answer the research aim/s in detail e.g. for qualitative interpretative phenomenological analysis might be considered	1. Consideration of some the research stakeholders e.g. use of pilot study with target sample but no stakeholder involvement in planning stages of study design.	2. Discussion of some of the key strengths and weaknesses of the study but not complete. e.g. several strengths/limitations explored but with notable omissions or lack of depth of explanation.
Salm Ward, T. C. (2023). "Things changed very quickly": Maternal intentions and decision-making about infant sleep surface, location, and position [Article]. Birth. https://doi.org/10.1111/birt.12793	3. Explicit discussion of the theories or concepts that inform the study, with application of the theory or concept evident through the design,	3. Explicit and detailed statement of aims/objectives in main body of report.	3. Specific description of the research setting and target population of study e.g. 'nurses and doctors from GP practices in	2. The study design can address the stated research aim/s but there is a more suitable alternative that could have been	2. Evidence of consideration of sample required to address the aim. e.g. the sample characteristics are described with	2. Basic explanation of rationale for choice of data collection tool/s. e.g. based on use in a prior similar study.	2. Structure and/or content of tool/s allow for data to be gathered broadly addressing the stated aim/s but could benefit from	3. Detailed description of each stage of the data collection procedure, including when, where and how data was gathered such that the	2. Some recruitment data but not a complete account e.g. number of people who were invited and agreed.	2. Basic justification for choice of analytic method selected e.g. method used in prior similar research.	2. Method of analysis can address the research aim/s but there is a more suitable alternative that could have been used or used in addition to offer a stronger analysis.	0. No mention at all	3. Thorough discussion of strengths and limitations of all aspects of study including design, methods, data collection tools, sample & analytic approach.

Salm Ward, T. C., Miller, T. J., & Naim, I. (2021). Evaluation of a multisite safe infant sleep education and crib distribution program [Article]. International Journal of Environmental Research and Public Health, 18(13), Article 6956. https://doi.org/10.3390/ijerph18136956	3. Explicit discussion of the theories or concepts that inform the study, with application of the theory or concept evident through the design,	3. Explicit and detailed statement of aims/objectives in main body of report.	3. Specific description of the research setting and target population of study e.g. 'nurses and doctors from GP practices in	3. The study design selected appears to be the most suitable approach to attempt to answer the stated research aim/s.	2. Evidence of consideration of sample required to address the aim. e.g. the sample characteristics are described with	3. Detailed explanation of rationale for choice of data collection tool/s. e.g. relevance to the study aim/s, co-designed with the	3. Structure and content of tool/s allow for detailed data to be gathered around all relevant issues required to address the	3. Detailed description of each stage of the data collection procedure, including when, where and how data was gathered such that the	3. Complete data allowing for full picture of recruitment outcomes e.g. number of people approached, recruited, and who	3. Detailed justification for choice of analytic method selected e.g. relevance to the study aim/s or comment around of the strengths	3. Method of analysis selected is the most suitable approach to attempt answer the research aim/s in detail e.g. for qualitative interpretative phenomenological analysis might be considered	1. Consideration of some the research stakeholders e.g. use of pilot study with target sample but no stakeholder involvement in planning stages of study design.	3. Thorough discussion of strengths and limitations of all aspects of study including design, methods, data collection tools, sample & analytic approach.
Shimizu, M., Park, H., & Greenfield, P. M. (2014). Infant sleeping arrangements and cultural values among contemporary Japanese mothers. Front Psychol, 5, 718. https://doi.org/10.3389/fpsyg.2014.00718	3. Explicit discussion of the theories or concepts that inform the study, with application of the theory or concept evident through the design,	3. Explicit and detailed statement of aims/objectives in main body of report.	3. Specific description of the research setting and target population of study e.g. 'nurses and doctors from GP practices in	3. The study design selected appears to be the most suitable approach to attempt to answer the stated research aim/s.	2. Evidence of consideration of sample required to address the aim. e.g. the sample characteristics are described with	3. Detailed explanation of rationale for choice of data collection tool/s. e.g. relevance to the study aim/s, co-designed with the	3. Structure and content of tool/s allow for detailed data to be gathered around all relevant issues required to address the	3. Detailed description of each stage of the data collection procedure, including when, where and how data was gathered such that the	3. Complete data allowing for full picture of recruitment outcomes e.g. number of people approached, recruited, and who	3. Detailed justification for choice of analytic method selected e.g. relevance to the study aim/s or comment around of the strengths	3. Method of analysis selected is the most suitable approach to attempt answer the research aim/s in detail e.g. for qualitative interpretative phenomenological analysis might be considered	0. No mention at all	3. Thorough discussion of strengths and limitations of all aspects of study including design, methods, data collection tools, sample & analytic approach.
Shin, S. H., Choi, C., Shih, S. F., Tomlinson, C. A., & Kimbrough, T. (2023). A Hospital-Based Infant Safe Sleep Intervention and Safe Sleep Practices Among Young Women: A Prospective Longitudinal Study [Article]. Maternal and Child Health Journal, 27(12), 2113-2120. https://doi.org/10.1007/s10995-023-03716-2	3. Explicit discussion of the theories or concepts that inform the study, with application of the theory or concept evident through the design,	3. Explicit and detailed statement of aims/objectives in main body of report.	3. Specific description of the research setting and target population of study e.g. 'nurses and doctors from GP practices in	3. The study design selected appears to be the most suitable approach to attempt to answer the stated research aim/s.	3. Detailed evidence of consideration of the sample required to address the research aim/s. e.g. sample size calculation or discussion of an iterative	3. Detailed explanation of rationale for choice of data collection tool/s. e.g. relevance to the study aim/s, co-designed with the	3. Structure and content of tool/s allow for detailed data to be gathered around all relevant issues required to address the	3. Detailed description of each stage of the data collection procedure, including when, where and how data was gathered such that the	3. Complete data allowing for full picture of recruitment outcomes e.g. number of people approached, recruited, and who	3. Detailed justification for choice of analytic method selected e.g. relevance to the study aim/s or comment around of the strengths	3. Method of analysis selected is the most suitable approach to attempt answer the research aim/s in detail e.g. for qualitative interpretative phenomenological analysis might be considered	0. No mention at all	3. Thorough discussion of strengths and limitations of all aspects of study including design, methods, data collection tools, sample & analytic approach.
Stiffler, D., Matemachani, S. M., & Crane, L. (2020). Considerations in Safe to Sleep messaging: Learning from African-American mothers. Journal for Specialists in Pediatric Nursing, 25(1), e12277. https://doi.org/https://dx.doi.org/10.1111/jspn.12277	3. Explicit discussion of the theories or concepts that inform the study, with application of the theory or concept evident through the design,	3. Explicit and detailed statement of aims/objectives in main body of report.	3. Specific description of the research setting and target population of study e.g. 'nurses and doctors from GP practices in	3. The study design selected appears to be the most suitable approach to attempt to answer the stated research aim/s.	2. Evidence of consideration of sample required to address the aim. e.g. the sample characteristics are described with	3. Detailed explanation of rationale for choice of data collection tool/s. e.g. relevance to the study aim/s, co-designed with the	3. Structure and content of tool/s allow for detailed data to be gathered around all relevant issues required to address the	3. Detailed description of each stage of the data collection procedure, including when, where and how data was gathered such that the	3. Complete data allowing for full picture of recruitment outcomes e.g. number of people approached, recruited, and who	3. Detailed justification for choice of analytic method selected e.g. relevance to the study aim/s or comment around of the strengths	3. Method of analysis selected is the most suitable approach to attempt answer the research aim/s in detail e.g. for qualitative interpretative phenomenological analysis might be considered	1. Consideration of some the research stakeholders e.g. use of pilot study with target sample but no stakeholder involvement in planning stages of study design.	3. Thorough discussion of strengths and limitations of all aspects of study including design, methods, data collection tools, sample & analytic approach.
Tully, K., Holditch-Davis, D., & Brandon, D. (2015). The Relationship Between Planned and Reported Home Infant Sleep Locations Among Mothers of Late Preterm and Term Infants. Maternal & Child Health Journal, 19(7), 1616-1623. https://doi.org/10.1007/s10995-015-1672-7	3. Explicit discussion of the theories or concepts that inform the study, with application of the theory or concept evident through the design,	3. Explicit and detailed statement of aims/objectives in main body of report.	3. Specific description of the research setting and target population of study e.g. 'nurses and doctors from GP practices in	3. The study design selected appears to be the most suitable approach to attempt to answer the stated research aim/s.	2. Evidence of consideration of sample required to address the aim. e.g. the sample characteristics are described with	3. Detailed explanation of rationale for choice of data collection tool/s. e.g. relevance to the study aim/s, co-designed with the	3. Structure and content of tool/s allow for detailed data to be gathered around all relevant issues required to address the	3. Detailed description of each stage of the data collection procedure, including when, where and how data was gathered such that the	3. Complete data allowing for full picture of recruitment outcomes e.g. number of people approached, recruited, and who	3. Detailed justification for choice of analytic method selected e.g. relevance to the study aim/s or comment around of the strengths	3. Method of analysis selected is the most suitable approach to attempt answer the research aim/s in detail e.g. for qualitative interpretative phenomenological analysis might be considered	0. No mention at all	3. Thorough discussion of strengths and limitations of all aspects of study including design, methods, data collection tools, sample & analytic approach.
Weil, L. E. (2020). Prevention of unintentional, sleep-related infant deaths: Current sleep practices, caregiver beliefs, and promotion of safe sleep practices through effective preventive interventions ProQuest Information & Learning]. US.	3. Explicit discussion of the theories or concepts that inform the study, with application of the theory or concept evident through the design,	3. Explicit and detailed statement of aims/objectives in main body of report.	3. Specific description of the research setting and target population of study e.g. 'nurses and doctors from GP practices in	3. The study design selected appears to be the most suitable approach to attempt to answer the stated research aim/s.	1. Evidence of consideration of the sample required e.g. the sample characteristics are described and appear appropriate to address the	3. Detailed explanation of rationale for choice of data collection tool/s. e.g. relevance to the study aim/s, co-designed with the	3. Structure and content of tool/s allow for detailed data to be gathered around all relevant issues required to address the	3. Detailed description of each stage of the data collection procedure, including when, where and how data was gathered such that the	3. Complete data allowing for full picture of recruitment outcomes e.g. number of people approached, recruited, and who	3. Detailed justification for choice of analytic method selected e.g. relevance to the study aim/s or comment around of the strengths	3. Method of analysis selected is the most suitable approach to attempt answer the research aim/s in detail e.g. for qualitative interpretative phenomenological analysis might be considered	0. No mention at all	3. Thorough discussion of strengths and limitations of all aspects of study including design, methods, data collection tools, sample & analytic approach.

Yuma-Guerrero, P. J., Duzinski, S. V., Brown, J. M., Wheeler, T. C., Barczyk, A. N., & Lawson, K. A. (2013). Perceptions of injury and prevention practices among pregnant and parenting teenagers. Journal of trauma nursing, 20(1), 3-9. https://doi.org/10.1097/JTN.0b013e3182866157	3. Explicit discussion of the theories or concepts that inform the study, with application of the theory or concept evident through the design, [illegible]	3. Explicit and detailed statement of aims/objectives in main body of report.	3. Specific description of the research setting and target population of study e.g. 'nurses and doctors from GP practices in [illegible]	3. The study design selected appears to be the most suitable approach to attempt to answer the stated research aim/s.	2. Evidence of consideration of sample required to address the aim. e.g. the sample characteristics are described with [illegible]	3. Detailed explanation of rationale for choice of data collection tool/s. e.g. relevance to the study aim/s, co-designed with the [illegible]	3. Structure and content of tool/s allow for detailed data to be gathered around all relevant issues required to address the [illegible]	3. Detailed description of each stage of the data collection procedure, including when, where and how data was gathered such that the [illegible]	3. Complete data allowing for full picture of recruitment outcomes e.g. number of people approached, recruited, and who [illegible]	3. Detailed justification for choice of analytic method selected e.g. relevance to the study aim/s or comment around of the strengths [illegible]	3. Method of analysis selected is the most suitable approach to attempt answer the research aim/s in detail e.g. for qualitative interpretative phenomenological analysis might be considered [illegible]	0. No mention at all	3. Thorough discussion of strengths and limitations of all aspects of study including design, methods, data collection tools, sample & analytic approach.
Zoucha, R., Walters, C. A., Colbert, A. M., Carlins, E., & Smith, E. (2016). Exploring Safe Sleep and SIDS Risk Perception in an African-American Community: Focused Ethnography [Article]. Public Health Nursing, 33(3), 206-213. https://doi.org/10.1111/phn.12235	3. Explicit discussion of the theories or concepts that inform the study, with application of the theory or concept evident through the design, materials and outcomes explored. e.g. [illegible]	3. Explicit and detailed statement of aims/objectives in main body of report.	3. Specific description of the research setting and target population of study e.g. 'nurses and doctors from GP practices in [x] part of [x] city in [x] [illegible]	3. The study design selected appears to be the most suitable approach to attempt to answer the stated research aim/s.	2. Evidence of consideration of sample required to address the aim. e.g. the sample characteristics are described with reference to the aim/s.	3. Detailed explanation of rationale for choice of data collection tool/s. e.g. relevance to the study aim/s, co-designed with the target population or [illegible]	3. Structure and content of tool/s allow for detailed data to be gathered around all relevant issues required to address the stated research aim/s.	3. Detailed description of each stage of the data collection procedure, including when, where and how data was gathered such that the procedure could be replicated.	3. Complete data allowing for full picture of recruitment outcomes e.g. number of people approached, recruited, and who completed with attrition data [illegible]	3. Detailed justification for choice of analytic method selected e.g. relevance to the study aim/s or comment around of the strengths of the method selected.	3. Method of analysis selected is the most suitable approach to attempt answer the research aim/s in detail e.g. for qualitative interpretative phenomenological analysis might be considered preferable for experiences vs. content analysis [illegible]	2. Evidence of stakeholder input informing the research. e.g. use of pilot study with feedback influencing the study design/conduct or reference to a project reference group established to guide the [illegible]	3. Thorough discussion of strengths and limitations of all aspects of study including design, methods, data collection tools, sample & analytic approach.
**Count of occurrences**													
**0**	0	0	1	0	4	2	1	1	2	6	0	40	6
**1**	4	0	3	1	12	4	1	3	3	2	1	10	1
**2**	12	3	5	6	16	8	10	4	16	6	2	7	10
**3**	44	57	51	53	27	46	48	52	39	46	57	1	43
**Percentage of occurrences**													
**No evidence (0)**	0%	0%	2%	0%	7%	3%	2%	2%	3%	10%	0%	69%	10%
**Limited (1)**	7%	0%	5%	2%	20%	7%	2%	5%	5%	3%	2%	17%	2%
**Some (2)**	20%	5%	8%	10%	27%	13%	17%	7%	27%	10%	3%	12%	17%
**Explicit/detailed (3)**	73%	95%	85%	88%	46%	77%	80%	87%	65%	77%	95%	2%	72%

Supplemental Table D: Literature Review Data Extraction Table

Study	Sample	Method	Approach	Reasons	Risk profile	Challenges	Solutions	Info needs
Bailey, C. (2016). Breastfeeding mothers' experiences of bed-sharing: A qualitative study.<br><br>Australia	Mothers-six breastfeeding mothers who bed-shared with their infants	qualitative interviews	risk minimisation	Breastfeeding<br>Comforting for infant and/or mother/parent<br>Monitoring/safety/protection<br>Better/more sleep<br>Bonding/Attachment/Relationship<br>Crying (unsettled baby)<br>Needed due to (Mother's) injury/caesarean	Breastfeeding-protective	Contact with MCHNs raised by most participants--felt they were under surveillance. Hospital no co-sleeping policies. Negative media portrayals of bed-sharing-frustrating	The mothers demonstrated a good understanding of safe bed-sharing practices. The sleeping spaces were well thought out and the mothers talked about how they had positioned the baby safely. All six mothers were able to clearly articulate bed-sharing risks and many mothers had bought items such as flat mattresses and bed rails to make the space safer and address fall risk. Trundle beds, extra single beds in room for siblings. Parents in separate beds.	Half of participants actively pretended they did not bed-share.<br>Sought out social supports who also bed-shared building a small circle of influence around their bed-sharing practices (often associated with Australian Breastfeeding Association groups)
Bailey, C., Tawia, S., & McGuire, E. (2020). Breastfeeding Duration and Infant Sleep Location in a Cohort of Volunteer Breastfeeding Counselors.<br>Australia	Mothers-174 women trained as Australian Breastfeeding Association counsellors	Cross-sectional-one group survey design	risk minimisation	Breastfeeding	Breastfeeding-protective	Not described	Not described	Author-The best way of addressing safety concerns is to ensure that mothers have excellent information about how to bed share safely with their infant (Ball et al., 2016). These recommendations include sleeping on a flat surface (not a sofa) and refraining from sleeping with an infant when the parent has consumed alcohol, drugs, or smoked cigarettes Network, 2017).
Ball, H. L., Taylor, C. E., & Yuill, C. M. (2021). A box to put the baby in: UK parent perceptions of two baby box programmes promoted for infant sleep<br>UK	Mothers and Fathers-In the LTAS study, 79 expectant mothers (and fathers In the BBE study, seventy-seven (77) parents	Feasibility study: Comparative study of perceptions between two cohorts	risk minimisation	Not discussed?	Smoke exposure<br>Young parental age	Box/PSS takes up too much space in bed, babies grow out of them, could not see baby to monitor in cardboard option<br>Disruption/change in routine	Several participants indicated they used the box to avoid direct bed-sharing: "I smoke, so I don't co-sleep (bed-share) because of SIDS. I just use the box."<br>Provided a daytime space for sleep to keep baby in proximity.	Authors-A missing component of both baby box programmes as implemented in the UK was the engagement of partners and wider family members in the interventions.
Bamber, A. R., Kiho, L., Upton, S., Orchard, M., & Sebire, N. J. (2016). Social and behavioural factors in non-suspicious unexpected death in infancy; experience from metropolitan police project indigo investigation<br>UK	Infants-477 infant deaths recorded in Project Indigo (2005 – 86, 2006 – 84, 2007 – 89, 2008 – 77, 2009 – 76, 2010 – 65)	Retrospective cohort. Descriptive analysis of routinely collected police data	neutral	Breastfeeding<br>Comforting for infant and/or mother/parent<br>Tradition (Culture)<br>Environmental (no cot, no room)	Preterm AND alcohol consumed AND smoke exposure AND social deprivation	chair/sofa sharing<br>infants with multiple risk factors and environments leaving no alternative but to co-sleep	Not described	Authors-Possible approaches therefore include both more simplified and universal advice regarding safe sleeping environments for infants, or interventions specifically targeted at this high-risk parent group, requiring a coordinated approach by government and charities.
Barrett, S., Barlow, J., Cann, H., Pease, A., Shiells, K., Woodman, J., & McGovern, R. (2024). Parental decision making about safer sleep practices: A qualitative study of the perspectives of families with additional health and social care needs.<br>UK	14 white-British mothers, with 2 fathers and one grandmother joining the mother, who had recent contact with child protection services in northeast England	Qualitative- In-depth semi-structured interview	Neutral- risk minimisation lens	Comforting for infant and/or mother/parent<br>Monitoring/safety/protection<br>Infant preference/Difficult temperament<br>Better/more sleep<br>Exhaustion/Fatigue<br>Bonding/Attachment/Relationship<br>Crying (unsettled baby)<br>Maternal instinct	Contact with child protection<br>Preterm<br>Alcohol<br>Drugs<br>Smoking<br>DV<br>Social deprivation<br>Medically fragile baby<br>No breastfeeding	Aware of advice application in real-life not always possible<br>Concerns about the risks of suffocation, strangulation or rolling onto their babes while sharing sleep<br>Routine valued as good parenting.<br>Disruption to routine was a challenge due to mental health or DV (stay in mother-baby unit to shelter).<br>Baby won't sleep anywhere but chest, mother trying to stay awake)<br>Not intending to share sleep but plans changed<br>Didn't disclose/lied to HP or friends	Each mother who shared sleep described self-generated 'instinctive' ways to mitigate known risks. E.g. Use of pillows to 'cradle' baby so she can't move and parent can't roll on baby, adjusting sleep own sleep position, light sleepers/ hyperaware	Social media as a primary source of information.<br>Families valued practical guidance and support from practitioners 'come around … and show us stuff'<br>HV a trusted go-to for specific questions<br>Parents considerably less likely to follow recommendations if they did not understand the protective mechanisms, and when they lacked skills or confidence to act on the information that they received this approach to giving information can also be ineffective for some, especially when information is delivered in a condescending, didactic style, without opportunities to ask questions
Barry, E. S., & McKenna, J. J. (2022). Reasons mothers bed-share: A review of its effects on infant behavior and development.<br><br>USA	Other-A review	Narrative Review, including research from anthropology, pediatrics, sociology, social work, public health, medicine, neurobiology, epigenetics, family studies, and psychology	risk minimisation	Breastfeeding<br>Comforting for infant and/or mother/parent<br>Monitoring/safety/protection<br>Better/more sleep<br>Bonding/Attachment/Relationship<br>Tradition (Culture)<br>Crying (unsettled baby)<br>Environmental (no cot, no room)<br>Disagree with danger<br>Maternal instinct	Breastfeeding-protective	Intentional/proactive bed-sharing (more prepared and safety considered) distinctly different to Unintentional/reactive shared sleep (unprepared, less informed about safety, more likely a riskier environment).<br>Lack of formal guidance = parent's solving safety concerns intuitively.<br>Western bedding not always a safe environment	Ball (2006) called this a "breast-baby axis of interaction" C-Shape positioning of mother's body<br>Breastsleeping	After a careful review of all existing SIDS studies up through 2019, the committee approved the following statement for paediatricians worldwide, "Safe bed-sharing is possible, and the existing evidence does not support the conclusion that bed-sharing among breastfeeding infants causes sudden infant death syndrome (SIDS) in the absence of known hazards." The committee went on to say that infant deaths can be potentially reduced by educating parents about how to bed-share safely and ending the stigma around bed-sharing. Consistent with this new protocol, our review highlighted the effects on the developing infant of mother's choice to bed-share, in relation to each reason for doing so. Without exception, the reasons mothers' bed-share have been shown to have beneficial effects on infant development, and recommendations against bed-sharing incur their own risks of altering developmental outcomes for infants whose mothers would otherwise do so.

Beth Howard, M., Parrish, B. T., Singletary, J., & Jarvis, L. (2022). Infant Safe Sleep in the District of Columbia: Better for Both.<br><br>USA	Mothers and Fathers-15 English-speaking caregivers of infants. 13 mothers and 2 fathers	Qualitative focus groups	risk elimination	Breastfeeding<br>Comforting for infant and/or mother/parent<br>Monitoring/safety/protection<br>Better/more sleep<br>Bonding/Attachment/Relationship<br>Tradition (Culture)<br>Crying (unsettled baby)<br>Disagree with danger<br>In balance of risks, felt bed-sharing safer<br>Maternal instinct	social deprivation	Family demands-"I can't stay up all night with him, I have to take care of other children during the day."<br>Conflicting information-"You have the nurses telling you one thing, your friends telling you how their babies slept, your mother telling you how you slept. Who are you going to listen to?"	"Room sharing, recommended by the American Academy of Pediatrics for infant sleep, represents an alternative to bed sharing that allows for proximity and closeness for intimacy and monitoring, while, unlike bed-sharing, decreasing the risk for SUID (Moon et al., 2022). Providing parents with the alternative of sharing a room rather than a bed may decrease bed sharing rates in this population."	Author-<br>Sources of knowledge of infant safe sleep recommendations were varied and including friends and family, media, and health care providers (Table 3). All participants reported an awareness of safe sleep recommendations, SUID, and SUID risk factors. Despite universal receipt of safe sleep knowledge from these sources, there were misconceptions, such as the safety of bed-sharing in the absence of risk factors
Capper, B., Damato, E. G., Gutin-Barsman, S., & Dowling, D. (2022). Mothers' Decision Making Concerning Safe Sleep for Preterm Infants: What Are the Influencing Factors?<br><br>USA	Mothers n=98 Mothers caring for preterm infant at home	Cross-sectional descriptive survey design	risk elimination	Comforting for infant and/or mother/parent<br>Monitoring/safety/protection<br>Better/more sleep	Prematurity	Concerns for wellbeing, needing to keep baby close for monitoring, anxious having baby out of arms, baby wouldn't sleep in crib	Not described	Mothers were asked whether there was anything they wanted to tell us about sleep practices and answered with responses consistent with change over time; "When we first arrived home, we were very strict on the rules given to us regarding sleep practices. Over time, we had to adapt those rules to fit our family, while keeping safety in mind" (28 weeks, current age 2 years 4 months). One mother recommended that healthcare providers prepare parents beyond the immediate posthospitalization period: There is SO much guidance on what to do right away when babies come home from the hospital, but no guidance on when to relax a little. I was paranoid about introducing a pillow at 2 years old…. I think in the push to educate parents of newborns, the other side of that is neglected—when is it appropriate to introduce those items to toddlers. When does the risk window close? (26 weeks, current age 4 years)
Caraballo, M., Shimasaki, S., Johnston, K., Tung, G., Albright, K., & Halbower, A. C. (2016). Knowledge, Attitudes, and Risk for Sudden Unexpected Infant Death in Children of Adolescent Mothers: A Qualitative Study.<br><br>USA	Mothers-43 adolescent mothers	Focus groups	risk elimination	Breastfeeding<br>Monitoring/safety/protection<br>Better/more sleep<br>Exhaustion/Fatigue<br>Bonding/Attachment/Relationship<br>Closer monitoring when baby is sick/post immunisations/teething/reflux	Teen mother/Young Maternal Age	Advice around bed-sharing conflicted with their intuition. Also received conflicting information between medical providers and participant's' own mothers. Baby would cry and not sleep in crib. Baby sleeps better so mother sleeps better.	Ignoring advice that didn't feel right. Devising own solutions to their safety concerns-Placing pillows around baby for protection while bed-sharing	Above all, mothers across all sites consistently felt that their instinct trumped advice from any source. One mother shared, "I'll get different information from my mom and doctor, and then. whatever I feel is right, I'll go on." Regarding physician advice, one mother stated, "Sometimes you just don't listen to their stuff. You—obviously you know what's right and what's not right." This was particularly true with bed-sharing; several mothers acknowledged being informed about the risks but still described an instinct that it is better to have the baby in their bed for close observation.
Clarke, J. (2016). Velcro babies: A Qualitative Study Exploring Maternal Motivations in the Night-time Care of Infants<br><br>NZ	Mothers--13 mothers of infants, living in a more socioeconomically deprived suburb in Christchurch NZ	Inductive qualitative design-semi-structured interviews	risk minimisation	Breastfeeding<br>Comforting for infant and/or mother/parent<br>Exhaustion/Fatigue<br>Bonding/Attachment/Relationship<br>Crying (unsettled baby)<br>Needed due to (Mother's) injury/caesarean	low socio-economic	"There is often tension between the 'frontstage' persona of mothers, who listen and follow 'expert' advice, and the 'back-stage' persona, where mothers respond to intuition and pragmatic needs."<br>Mothers struggling to follow the expert advice and still meet their own needs and their infant's.	Mothers did not dismiss risk; they are very aware and take steps to mitigate risk using their knowledge and understanding of the situation.<br>her partner slept in a different room for the first few months. She described how important it was for her that the baby had her own space in the bed, with her own blankets so that she 'couldn't get caught up in the adult blankets'.	Dependence on midwives in early days for advice: 'My midwife was amazing; saw us every day for the first week and then every couple of days after that... and you text her and she'll reply within an hour or call you just to see how you were going…she was really supportive; you could ask her anything.' P, 56. Some trusted Plunket as a trusted source for information and advice whereas other dismissed professional expert advice or reported a distrust of expert advice, trusting intuition over experts.<br>Author--"The technico-scientific perspective would suggest that it is a relatively simple process of telling mothers of risks which the 'good mother' will obediently follow. The comments of the mothers above, highlight that the process is more complex than this. Just as we cannot predict how mothers will assess risk, we also cannot predict how people will interpret information about risks." P.59-60.<br>"It is difficult for mothers to know who and what to trust when they receive conflicting advice from those they consider to be experts. It is understandable that they will then look to themselves and others to decide how best to mitigate any perceived risks. Indeed, if health professionals, such as the midwives above, are using their own judgement to assess and manage risk at a personal level, it hints at an undermining of the technico-specific perspective that risks are even 'objective facts' (Bradbury, 1989, p. 382). Rather it suggests, as does Lupton (2013b), that the identification of 'risks' takes place in the specific sociocultural, political and historical contexts in which we are located' (p. 21)." P. 61
Cole, R., Young, J.,	Mothers (97%)-n3341	cross-sectional	risk	Breastfeeding	mixed	Difficulty reported with the	some substitute actions, often intended	Author-Parents reported difficulty with sleeping or care

Kearney, L., & Thompson, J. M. D. (2021). Challenges parents encounter when implementing infant safe sleep advice. Australia	caregivers in Australia with young infants	survey	minimisation	Comforting for infant and/or mother/parent<br>Exhaustion/Fatigue<br>In balance of risks, felt bed-sharing safer		recommendation for infant to sleep in *own sleep space*. Falling asleep feeding, settling baby, fatigue/exhaustion	by caregivers to increase protection and comfort such as surrounding the infant by pillows, inadvertently increased SUDI risk.	practices related to safe sleep advice-10.7% had difficult with 'own sleep space advice'
Cowan, S., Bennett, S., Clarke, J., & Pease, A. (2013). An evaluation of portable sleeping spaces for babies following the Christchurch earthquake of February 2011.<br><br>NZ	Mothers (83%) + 'others'--100 NZ families who received a Portable Sleep Space (PSS) after an earthquake disrupted their sleep conditions	convenience sample, online survey	risk minimisation	Monitoring/safety/protection<br>Change in routine	Disrupted routine<br>Smoke exposure in pregnancy<br>prematurity<br>low birth weight<br>crowded living<br>frequent moving	Earthquake concerns-<br>wanting to keep baby close to protect them from physical danger, ongoing aftershocks<br>Extreme parental anxiety<br>Disrupted routine and frequent moving<br>Baby outgrew them<br>13% bed-sharing after use of PSS	Portable Sleep Space (SSP) provided to eligible families-Criteria for getting a PSS were babies aged less than 4 months and any of the other evidence-based factors for increased risk of sudden infant death: smoke-exposure in pregnancy, prematurity and low birthweight. These were extended to include 'earthquake-related concerns' influencing infant sleep safety, such as crowded living, no baby bed, frequent moving and extreme parental anxiety.	Author-99% reported having received a thorough safety briefing across all key topics, from 'making up the PSS' (94%) and 'how sleep position affects breathing' (92%), to 'safe places to put it' (85%) and 'rules of protection' (83%), and 'support with settling' (77%) and 'who needs one and why' (73%). 97% passed this knowledge on to others.
Crane, D., & Ball, H. L. (2016). A qualitative study in parental perceptions and understanding of SIDS-reduction guidance in a UK bi-cultural urban community.<br>UK	Mothers-46 mothers-25 White British origin, 21 Pakistani origin-with infants	In-depth narrative interviews	risk minimisation	Breastfeeding<br>Monitoring/safety/protection<br>Tradition (Culture)		White British mothers avoided co-sleeping by sofa sharing and propping themselves up during feeds at night.<br>Feeling guilty if they bring baby to bed knowing it is against guidance<br>Pakistani mothers not trusting guidance with belief it is written for English mothers.	Not described	Recommendations to avoid bed-sharing with an infant were known to the Pakistani mothers who expressed strong beliefs about this practice and dismissed guidance that babies should sleep in a cot near the parents' bed, feeling this information was not directed to them: "Most of the stuff they write is meant for the English mothers and not the Asian women. I know what's best for my babies and I like them with me at night." P.3<br>Author-this study found that UK SIDS intervention strategies were limited in their effect on both Pakistani and white British families in Bradford as mothers were aware of, but did not implement, the risk-reduction guidance offered.
Cunningham, H. M., Vally, H., & Bugeja, L. (2018). Bed-Sharing in the First 8 Weeks of Life: An Australian Study.<br><br>Australia	Mothers-1126 Australian parents of 8-week-old infants	Cross-sectional survey	exploratory-leaning toward risk minimisation	Breastfeeding<br>Comforting for infant and/or mother/parent<br>Better/more sleep<br>Exhaustion/Fatigue<br>Bonding/Attachment/Relationship<br>Convenience/Ease<br>Tradition (Culture)<br>Environmental (no cot, no room)<br>Disagree with danger<br>Closer monitoring when baby is sick/post immunisations/teething/reflux<br>Needed due to (Mother's) injury/caesarean<br>Change in routine (away from home)<br>Not wanting to wake other children<br>Bed-shared in hospital<br>Play		While bed-sharing on an adult bed may be considered a safer option than on a sofa/couch, it is also with its risks (Beal and Byard 2000). With most adult beds being raised off the floor, bed-sharing on these elevated surfaces adds the risk of infant falls (Beal and Byard 2000)	In response to risk of falls, parents have moved the beds against a wall or put their infant between them, which both have increased the risk of SUDI (Beal and Byard 2000). Keeping the mattress on the floor is considered a safer option and in this study 17 (3.4%) infants were reported to be bed-sharing on mattresses on the floor.	Author-A total of 331 (78.5%) of these mothers who did not plan to bed-share had one or more known SUDI risk factors present. The difference between 'the plan' and the reality is an important understanding when developing infant safe sleeping messages, as recommendations cannot be led by what parents plan to do
Doering, J. J., Lim, P. S., Salm Ward, T. C., & Davies, W. H. (2019). Prevalence of unintentional infant bed-sharing.<br><br>USA	77% mothers 23% fathers--375 parents-77% mothers, 74% Caucasian	exploratory survey	risk minimisation	Breastfeeding<br>Comforting for infant and/or mother/parent<br>Exhaustion/Fatigue<br>Closer monitoring when baby is sick/post immunisations/teething/reflux	medically complex infant	exhaustion, negotiating the needs of a medically complex infant and sleep.	Clinicians can help to normalize the occurrence of unintentional bed-sharing by asking if it has happened, and if so, where. Subsequently, clinicians can engage in supportive conversations about reducing future risk in these situations, for example, by choosing a firm, flat surface, or by providing practical suggestions for avoiding accidentally falling asleep, such as setting a timer to rouse parent after 20 min or arranging for help with infant caregiving to promote parental sleep.	Author-Clinicians can help to normalize the occurrence of unintentional bed-sharing by asking if it has happened, and if so, where. Subsequently, clinicians can engage in supportive conversations about reducing future risk in these situations, for example, by choosing a firm, flat surface, or by providing practical suggestions for avoiding accidentally falling asleep, such as setting a timer to rouse parent after 20 min or arranging for help with infant caregiving to promote parental sleep.
Doering, J. J., Marvin, A., & Strook, S. (2017). Parent decision factors, safety strategies, and fears about infant sleep locations.<br><br>USA	98% mothers 2% fathers--The 49 caregivers represented ten different countries and lived in the continents of Asia (n = 4), Europe (n = 4), North America (n = 38), Oceania (n=2), and South	pilot study used a mixed-methods, exploratory, descriptive, non-experimental design	exploratory	Comforting for infant and/or mother/parent<br>Monitoring/safety/protection<br>Better/more sleep<br>Convenience/Ease		Worries included smothering, keeping baby an appropriate temperature	Parents maximized safety by providing a clear sleep surface, no blankets, no toys, sleep sack use, and a firm mattress.<br>The 11% who reported sharing a sleep surface and having no worries or fears deserves further investigation to explore the steps parents take to secure such peace of mind, investigate the concrete	Author-In a clinical context, asking for the thought behind a parent's decision, the safety strategies used, and their worries, may help nurses obtain a more comprehensive assessment and more effectively discuss practical risk-reduction strategies rather than telling parents simply that their choices are "good" or "bad"

	America (n=1)						steps parents take to reduce hazards in shared sleep environments, and quantify the effectiveness of those hazard reduction methods.	
Ellis, C. (2019). Safely Sleeping?: an Exploration of Mothers' Understanding of Safe Sleep Practices and Factors that Influence Reducing Risks in Their Infant's Sleep Environment<br><br>UK	Mothers-15 mothers aged between 16-21 years, presented with at least one other factor: smoking; misuse of drugs or alcohol (in excess of 2 units/day in a given week, unemployment or low income; reported housing issues (rented, overcrowding, homelessness/sofa surfing).	qualitative approach using Interpretative Phenomenological Analysis (IPA). Serial in-depth interviews	exploratory	Comforting for infant and/or mother/parent<br>Exhaustion/Fatigue<br>Infant preference/Difficult temperament	Teen/Young Maternal Age + smoking; misuse of drugs or alcohol (in excess of 2 units/day in a given week, unemployment or low income; reported housing issues (rented, overcrowding, homelessness/sofa surfing).	Turning on lights to stay awake while feeding and then unable to get back to sleep after<br>The time after a baby outgrew their Mose's basket also coincided with the time when a mother's confidence increased, and their advice-seeking decreased. Participants often 'worked out' their own solutions. Understanding safety recommendations to cot safety did not transfer to other sleep scenario. Sleep environments that were non-nighttime varied greatly-make-shift 'beds' on pillows and blankets or on the sofa, in car seats, on soft bean bags and bouncy chairs for example, or falling asleep on an adult, on a sofa	Participants appear to be able to 'consciously' apply safe sleep recommendations some of the time, and particularly when the infants were in the Moses basket, but this application is inconsistent once the infant outgrows the Moses basket. My observations support that these mothers 'subconsciously' do what comes naturally to them, they resolve issues that arise but apparently without consciously thinking them through, this is reactive not planned, they do whatever works, or they accept advice without question or challenge<br><br>Use of pillows to soften a hard mattress<br>Turning on lights and walking around to avoid falling asleep in bed when baby needs a cuddle	Author-Own judgement was applied along with common sense and information was accepted if it seemed 'believable'. Not always sure where their knowledge had come from 'just knew'. Some trusted their midwives and family as sources. "When trusted relationships were a feature of service provision, the decision-making processes for these young mothers were more likely to be underpinned by better information and supported both the challenge of outdated information and translation into practice." P.189 (not always consistent though)
Fangupo, L. J., Lucas, A. W., Taylor, R. W., Camp, J., & Richards, R. (2021). Sleep and parenting in ethnically diverse Pacific families in southern New Zealand: A qualitative exploration.<br><br>NZ	Mothers, Grandmothers, male caregivers (fathers?)-15 caregivers who identified as Ethnically Diverse Pacific Families (EDPF) 9 mothers, 3 fathers, 2 grandmothers	Qualitative interviews	Exploratory	Comforting for infant and/or mother/parent<br>Infant preference/Difficult temperament	Ethnically Diverse-Pacific families	Baby won't settle without touch and proximity. In many cases, parents had initially tried to follow advice closely, but changed their practices or became more "flexible" if they noticed that other practices (such as shared sleep spaces or allowing baby to sleep on their tummy) appeared to result in their child settling to sleep better or staying asleep for longer.	Not described	Author-It appears that there is a need for healthcare providers to move away from highly prescriptive sleep safety and health messages, and instead adopt non-judgmental, practical "share and discuss" engagement styles with families. This would encourage full and open conversations between providers and families whereby any discrepancies between recommendations and actual practices could be safely discussed and the risks and benefits (with regard to both sleep safety, and sleep health) would be clearly understood and appraised by both parties.
Feld, H., Ceballos Osorio, J., Bahamonde, M., Young, T., Boada, P., & Rayens, M. K. (2021). Poverty and Paternal Education Associated With Infant Safe Sleep Intentions in a Peri-Urban Community in Ecuador.	Women-100 pregnant women	Cross-sectional descriptive design. Self-report surveys	risk elimination	Breastfeeding<br>Comforting for infant and/or mother/parent<br>Monitoring/safety/protection<br>Convenience/Ease<br>Tradition (Culture)<br>Environmental (no cot, no room)		No crib<br>Women in this study also reported high rates of bed-sharing intentions, history of bed-sharing, and breastfeeding. The analysis of the narrative rationales about bed-sharing suggest that poverty reinforces this practice. Some mothers indicated they did not have other sleep options, and some reported keeping infants in mother's bed protects them from other hazards related to animals, pests, and earthquakes. Women in the study also stated that at night it is easier to care for and breastfeed the infant in bed.	Not described	Not described
Gaertner, V. D., Malfertheiner, S. F., Postpischil, J., Brandstetter, S., Seelbach-Göbel, B., Apfelbacher, C., Melter, M., Kabesch, M., Ambrosch, A., Arndt, P. A., Baessler, A., Berneburg, M., Böse-O'Reilly, S., Brunner, R., Buchalla, W., Franke, A., Häusler, S., Heid, I., Heinze, S., . . . Wellmann, S. (2023). Implementation of safe infant sleep recommendations during night-time sleep in the first year of life in a German birth cohort Germany	Mothers-1400 mothers of infants in Germany	Quantitative interviews and surveys at multiple timepoints	risk minimisation	Breastfeeding	smoke exposure	Bed-sharing rates increased despite intention not to bed-share before birth.<br>Keeping baby warm enough-used sleep sack + additional blanket<br>Use of baby nests and pillows inside sleep environment<br>Use of bedside sleepers strong despite no studies on SIDS and bedside sleepers or safety standards in this jurisdiction	Breastfeeding while bed-sharing	Author-Safety guidance on proper installation of three-sided/bedside sleep space<br>Considering that two-thirds of families in our survey obviously did not follow the blanket recommendation not to bed-share, it appears that the current practice of counseling is not working. Whether a more targeted approach, focusing on specific high-risk infants, might be overall more effective needs to be clarified in future studies. Regardless of this thought for future advancement of SIDS counseling, all parents should be educated by the health care professionals about the potential benefits and the risks of bed-sharing.
Gaydos, L. M., Blake, S. C., Gazmararian, J. A., Woodruff, W., Thompson, W. W., & Dalmida, S. G. (2015). Revisiting Safe	Mothers and Medical providers-60 African American mothers of young infants, 20 medical providers who serve new	Focus groups (with mothers) and telephone interviews (with providers)	risk minimisation	Monitoring/safety/protection<br>Better/more sleep<br>Convenience/Ease	low-income mothers<br>African American	Lack of access to risk minimisation advice meant participants devised their own solutions to address their fears for their baby's safety.<br>Providers do not actively counsel their	sleeping on chairs or sofas as a *solution* to avoid bed-sharing. Adding blankets and pillows to mitigate risk.<br>"Because clinicians typically give the "gold standard" recommendations, but	Author-Our findings may suggest a need for more community involvement in developing appropriate counseling strategies and recommendations to reduce unsafe sleeping behaviors among African-American mothers and infants, rather than a renewed focus

Sleep Recommendations for African-American Infants: Why Current Counseling is Insufficient.<br><br>USA	mothers					patients on risk mitigation techniques if they decide to bed-share.<br>"Another paediatrician noted that her practice instituted a policy requiring bed sharing mothers to sign a waiver stating that they understand the increased SIDS risk."	do not provide risk mitigation information, many mothers are using their own judgment to ‘‘reduce’’ SIDS risk when they decide to bed share or place the child in a supine sleeping position. Unfortunately, many of the actions intended to increase safety and comfort, such as surrounding the infant by pillows, have the effect of further increasing SIDS/SUID risk. It appears that current clinical practices reported in our study are not translating to safe sleeping behaviours among African American patients, who in our study demonstrate a poor understanding of risk reduction techniques."	exclusively on provider training.
George, M., Theodore, R., Richards, R., Galland, B., Taylor, R., Matahaere, M., & Te Morenga, L. (2020). Moe Kitenga: a qualitative study of perceptions of infant and child sleep practices among Māori whānau<br><br>NZ	Mothers-14 Māori families- 11 interview mother only, 3 interviews with 2 parents	Qualitative interviews	Exploratory (aiming for risk minimisation)	Breastfeeding<br>Monitoring/safety/protection<br>Better/more sleep<br>Exhaustion/Fatigue<br>Bonding/Attachment/Relationship<br>Tradition (Culture)<br>Closer monitoring when baby is sick/post immunisations/teething/reflux<br>Needed due to (Mother’s) injury/caesarean<br>Cheaper warmth/cooling	Māori	large families, sleeping in same room norm and preference. Did not feel safe telling health professional about it-"In spite of all of these reasons for bed-sharing, several parents reported purposefully not telling health professionals about it. For example, Christina said, 'I used to lie to Plunket because they’d say, “Do you sleep with your baby?” They’d say, “she should always be in her own bed,” and I’d say, “Yeah, yeah.” ’Cause I’d find they’d just make me feel bad and I was just trying to get through.' This fear and avoidance of punitive discussions around bed-sharing resonated with Olivia: “. . . because of the whole making me feel like I was a bad parent because she was sleeping with me.”"	not smoking if sharing a bed. Bassinet to start the night. Bassinet until 3 months then in with her. Authors mention Pēpi-pods®//Wahakura but not mentioned by any families	Authors-We therefore note that contemporary safe sleep messages to avoid bed-sharing must be recognised as going against what feels “right” or is easiest in light of competing family priorities and resources available for many whānau. Alternative approaches, such as including information and access to wahakura (woven flax basket allowing babies to sleep safely in the caregiver’s bed) or pēpi pods, explaining why not smoking and drinking is important, and urging caution in the use of blankets due to the cold may be more relevant for some whānau..
Gettler, L. T., Kuo, P. X., Sarma, M. S., Lefever, J. E. B., Cummings, E. M., McKenna, J. J., & Braungart-Rieker, J. M. (2021). US fathers’ reports of bonding, infant temperament and psychosocial stress based on family sleep arrangements.<br>USA	Fathers-195 Midwestern US Fathers	Qualitative survey tools	Exploratory	Comforting for infant and/or mother/parent<br>Bonding/Attachment/Relationship<br>Infant preference/Difficult temperament<br>Crying (unsettled baby)	Fathers (non-breastfeeding parent)	Difficult infant temperament-our results also show indications that (reactive) bed-sharing is linked to paternal perceptions of negative infant temperament and fathers’ reports of parenting stress pertaining to difficulties with their child.<br>Shared sleep safety when father is in bed (with or without the mother)	Not described	Not described
Gilmour, H., Ramage-Morin, P. L., & Wong, S. L. (2019). Infant bed sharing in Canada	Women-5329 Canadian mothers	Cross-sectional survey	Exploratory	Breastfeeding<br>Better/more sleep<br>Exhaustion/Fatigue<br>Environmental (no cot, no room)<br>Closer monitoring when baby is sick/post immunisations/teething/reflux	Mixed	Need some sleep<br>Unsettled or sick child<br>No crib	Not describe	Not described
Gustafsson, S., Jacobzon, A., Lindberg, B., & Engström, Å. (2022). Parents' strategies and advice for creating a positive sleep situation in the family.<br>Sweden	Mothers (84.2%) and fathers (15.8%)<br>76 parents	Qualitative online survey	Exploratory	Comforting for infant and/or mother/parent<br>Better/more sleep<br>Bonding/Attachment/Relationship		Not described	Not described	Author-Due to the variations in preferences and needs, the nurse should start from a person-centred or family-centred perspective and explore the specific circumstances of each family. Suggested implications are to advocate safe co-sleeping, having routines, prioritising, helping each other as parents, and/or asking for help from others, so the parents also can get some sleep.
Hamadneh, S., Kassab, M., Hamadneh, J., & Amarin, Z. (2016). Sudden unexpected infant death in Jordan and the home environment<br><br>Jordan	Mothers-604 mothers-394 citizens and 210 refugees in Jordan	semi-structured interview completed during a face-to-face interview	risk elimination		Refugee/unstable accommodation AND high smoke exposure and poorly ventilated sleep environments	Challenges keeping infant warm enough during Winter in poorly ventilated accommodation with high use rates of wood, kerosene or gas heaters (73%) with 10 % regularly ventilating the room<br>Blankets in sleep space (67%)<br>Head covering through Winter (84%)<br>Side sleep positioning is cultural norm (88%)<br>Wedging using pillows and bumpers common (55%)<br>Almost 60% unaware of SUDI risks	Not described	Not described

						(higher rate among Bedouin and rural groups)		
Hauck, F. R., Tanabe, K. O., McMurry, T., & Moon, R. Y. (2015). Evaluation of Bedtime Basics for Babies: A National Crib Distribution Program to Reduce the Risk of Sleep-Related Sudden Infant Deaths<br><br>USA	96% mother and 4% other-3303 families with financial need for a free crib + at least 1 African American, American Indian or Alaska Native, maternal smoking, pre-term or low birth weight, or sibling of a SIDS infant, 1729 through to follow-up	pre and post intervention surveys	risk elimination	Environmental (no cot, no room)	African American, American Indian or Alaska Native, maternal smoking, pre-term or low birth weight, or sibling of a SIDS infant	Bed-sharing due to lack of access to a crib? "When asked where their infant would have slept had they not received the BBB crib, 32 % responded that they would have slept in their parents' bed or in bed with another person (other than parents or siblings)."<br>16% of participants still bed-shared after intervention with no risk minimisation guidance	Free crib provision? No solutions to assist in shared sleep safety	Not described
Hauck, F. R., Moon, R. Y., Kerr, S. M., Corwln, M. J., Heeren, T., Colson, E., Parker, M. G., & Kellams, A. (2024). Mothers Falling Asleep During Infant Feeding.<br>USA	1259 mothers who responded to the postpartum survey (mean infant age, 11.2 weeks).	Quantitative - randomized trial-survey	Risk elimination-with a risk minimisation as an add on	Breastfeeding<br>Comforting for infant and/or mother/parent	Mixed-<br>Breastfeeding-protective<br>Formula feeding<br>Priority population-African-American	A total of 28.2% of mothers reported FAF usually or sometimes in the last 2 weeks, 83.4% of whom reported that FAF was unplanned.<br>Challenges – sitting on couch or sofa because they were told beds are unsafe, trying to stay awake to feed, not to disturb others<br>Breastfeeding release of oxytocin and relaxation effects	Feeding on couch or sofa to avoid Bedsharing	Author- repeats AAP advice but adds – "that infants be brought into the adult bed for feedings if there is a chance that the parent may FAF and that infants be placed back in their own sleep space when the parent awakens." And remove pillows, blankets and other soft items
Herman, S., Adkins, M., & Moon, R. (2015). Knowledge and Beliefs of African-American and American Indian Parents and Supporters About Infant Safe Sleep.<br><br>USA	54 mothers and 13 female supporters and 13 male supporters--73 African-American, or American-Indian caregivers	Focus groups	Exploratory (leaning toward risk elimination)	Breastfeeding<br>Comforting for infant and/or mother/parent<br>Monitoring/safety/protection<br>Better/more sleep<br>Exhaustion/Fatigue<br>Convenience/Ease<br>Infant preference/Difficult temperament<br>Disagree with danger	African American, American Indian	Challenges with health professionals and materials not addressing the differences among infants in terms of sleep behaviour and environmental comfort.<br>Sleeping baby in crib led to increased disruption to mother's sleep as she kept waking to check on her baby.	Direct request for risk minimisation guidance from providers-"I think you should give a tip on how there a safe way can be the baby can sleep in the bed instead of just saying, no, you can't do it. [African-American Mother]"<br>"Especially if you are having that many problems putting your [baby to bed], trying to follow safe sleep steps and you're having problems. I think if there was something that … had a list of safe alternatives, … instead of waking up every 15 min to adjust your baby, you'd actually read it to see if something can accommodate you. [American Indian Mother]"	Parents expressed the opinion that health professionals and educational materials did not address the differences among infants in terms of sleep behaviour and environmental comfort<br>Direct ask for risk minimisation advice for shared sleep<br>Parents suggested that their decisions about how and where the infant slept were often driven, not by the recommendations or by safety concerns, but by the parent's need for sleep and, therefore, the parent's need for the infant to go to sleep (Q16). Some participants expressed the desire for safe sleep guidelines to include alternative strategies to keep the infant safe while promoting sleep
Hirsch, H. M., Mullins, S. H., Miller, B. K., & Aitken, M. E. (2018). Paternal perception of infant sleep risks and safety.<br><br>USA	49 fathers/grandfathers/uncles/cousins caregivers of infants. 67% African-American, 33% White	Focus Groups	Risk elimination	Breastfeeding<br>Comforting for infant and/or mother/parent<br>Monitoring/safety/protection<br>Better/more sleep<br>Exhaustion/Fatigue<br>Bonding/Attachment/Relationship<br>Convenience/Ease<br>Infant preference/Difficult temperament<br>Crying (unsettled baby)<br>Disagree with danger<br>Closer monitoring when baby is sick/post immunisations/teething/reflux	African-American, Fathers (non-breastfeeding parent)	Most owned a crib/pack 'n' play//bassinet but due to Infant temperament/preferences-does not like the pack 'n' play.<br>Baby only sleeps on parent(s).<br>Media portrayal of dads in unsafe shared sleep environments-One participant asked, "Am I supposed to fall asleep with the baby on the couch and get a picture like this? I think a lot of dads don't really know what they are supposed to be doing the first few months."<br>No guidance targeted at fathers	Risk of falling-very large king-size bed, baby in middle, another had pushed their bed against a wall<br>Smothering-believed their infant was far enough away that they would not roll on top of them: "…we know that me and my wife, we don't move when we sleep, when we pass out we kinda wake up in the same position… so it's never been an issue as to we're scared of rolling over on the child…."<br>Bed-sharing to avoid 'crib death' in crib<br>Sleeping between father's arms like a cradle	Participants were asked to identify suggestions for future infant safe sleep messages targeted to male caregivers. Overwhelmingly, participants wanted the content and tone of safe sleep messages to be factual, brief, and serious.<br>Most participants emphasized that they were involved in childcare since birth and that messages should recognize that.<br>The participants strongly advocated for positive images of male caregivers<br>promoting safety rather than as unskilled in childcare: "I can tell you one thing I don't wanna see …I don't wanna see things where dad's an idiot; a bumbling idiot." Regarding delivery method for safe sleep messages, participants emphasized a desire for quick communication methods including billboards, posters, commercials, and social media. Options for media targeting male consumers, including internet radio, video games, phone apps, and sports venues were also endorsed. Locations for messages primarily included obstetrician and pediatrician's offices and hospital delivery and discharge. Many stated that they, "…would be most likely to listen to stuff and pay attention to stuff in a pediatrician office.
Huber, R., Menon, M., Russell, R. B., Smith, S., Scott, S., & Berns, S. D. (2024). Community infant safe sleep and breastfeeding promotion and population level-outcomes: A mixed methods study.<br><br>USA	Providers + PRAMS and OPAS data-7 perinatal service providers participating in NAPPSSIIN-National Action Partnership to Promote Safe Sleep Improvement and Innovation Network<br>+<br>2019 PRAMS and OPAS	Mixed methods-intervention study	risk minimisation (used risk mitigation)		mixed	Elders of the family need to be on board with practices to see them continue post intervention	several informants expressed interest in promoting alternative infant sleep practices in sustainability planning. One informant explained how their sustainability goals involved risk-mitigation messaging: "[We want to] make it okay for home visitors to talk about reducing the risk of co-sleeping…we dance around [the topic], but we have not addressed it. I want to remove the [feeling] that they can only	HPs want to be able to deliver risk mitigation messaging

	data						share prescriptive [ISS solutions] out of concern of losing their job.”	
Hutchison, B. L., Thompson, J. M. D., & Mitchell, E. A. (2015). Infant care practices related to sudden unexpected death in infancy: A 2013 survey<br>NZ	Women-172 mothers of infants	postal survey (quant and qual)	exploratory	Breastfeeding<br>Short naps/Occasional		Challenges when baby won't settle, staying awake breastfeeding and shared sleep for naps	Not described	Not described
Hwang, S. S., Parker, M. G., Colvin, B. N., Forbes, E. S., Brown, K., & Colson, E. R. (2021). Understanding the barriers and facilitators to safe infant sleep for mothers of preterm infants<br><br>USA	23 mothers of preterm infants	in-depth qualitative interviews	Exploratory (leaning towards risk minimisation)	Breastfeeding<br>Comforting for infant and/or mother/parent<br>Monitoring/safety/protection<br>Better/more sleep<br>Exhaustion/Fatigue<br>Closer monitoring when baby is sick/post immunisations/teething/reflux<br>In balance of risks, felt bed-sharing safer	Prematurity	Interventions that have demonstrated effectiveness in improving safe infant sleep practices in the healthy term population may not resonate with mothers of preterm infants who have had starkly different prenatal, birth, and postnatal experiences.<br>Unsafe sleep practices when infant in care of other family members including Grandmothers-sleeping baby on couch, couch cushions and blankets. Parent felt they lacked control over the situation when not present<br>Need to closely monitor premature infant at home.<br>Father in bed moves around a lot	Risk minimisation support-One mother who was struggling with lack of sleep discussed how other groups aided her risk mitigation approach and allowed her to have more control over the situation. She stated, “…the Leche League and the nurse family partners coached me on [co-sleeping] and how to make it safe. So I felt confident in doing it and that was really the game changer because I wasn’t sleeping. So once we found a way to [safely co-sleep], that’s what made a huge difference for us was the co-sleeping.”<br>C-Shape positioning<br>baby in middle of bed to mitigate risk of falling	Besides health care providers, mothers mentioned the impact of their partners, other children, parents, grandparents, and friends on their infant sleep practices. Some mothers reported that advice from friends or family was not concordant with recommendations from health care providers and thus decision-making about infant sleep was more complicated.<br>Author-It is likely that the interventions that have demonstrated effectiveness in improving safe infant sleep practices in the healthy term population may not resonate with mothers of preterm infants who have had starkly different prenatal, birth, and postnatal experiences. Thus, population-based studies of factors that impact decision-making about infant sleep practices for families of preterm infants are needed in order to inform the development of intervention trials specifically targeted to the preterm population.
Hwang, S., Rybin, D., Heeren, T., Colson, E., & Corwin, M. (2016). Trust in Sources of Advice about Infant Care Practices: The SAFE Study.<br><br>USA	3297 mothers of infants	Postal survey	Exploratory			Variation in trust and information given-Generally lower levels of trust in sources about bed-sharing across the surveyed options-Doctor (60%), Nurses (25%), Family (30%), Friends (12%), Media (11%). Doctors were more trusted for Sleep position, Feeding or Vaccination advice. Black non-Hispanic respondents reported even lower levels of trust in these sources for bed-sharing information.	Not described	Not described
Kadakia, A., Joyner, B., Tender, J., Oden, R., & Moon, R. Y. (2015). Breastfeeding in African Americans May Not Depend on Sleep Arrangement.<br><br>USA	Mothers-Survey 412 African American parents<br>FG//Interviews-83 African-American parents	cross-sectional mixed methods-survey/focus groups/interviews	risk elimination	Breastfeeding<br>Ease of feeding (not breastfeeding)	African-American<br>social deprivation<br>formula feeding	Challenges experienced trying to successfully breastfeed/feed baby and not share sleep at all<br>Our findings suggest that bed-sharing is associated with breastfeeding in lower SES groups, but that skepticism that breast milk is better than formula may contribute to low breastfeeding rates in African Americans.<br>Lack of breastfeeding support from-workplaces, access to leave to be with infant in their first 12 weeks, difficult to breastfeed without support of family and friends and crucially the infant’s father	Not described	Author-Women were significantly more likely to usually bed-share or to have bed-shared the previous night if they were breastfeeding (either exclusively or any), compared with those who were exclusively formula-fed.<br>Health care providers may need to reinforce information about breastfeeding benefits while providing information about safe sleep arrangements to optimize outcomes for infants.
Lerner, R. E., Camerota, M., Tully, K. P., & Propper, C. (2020). Associations between mother-infant bed-sharing practices and infant affect and behavior during the still-face paradigm.<br>USA	63 African American mother-infant dyads	Observational – qualitative via survey and video	exploratory	Breastfeeding<br>Comforting for infant and/or mother/parent<br>Crying (unsettled baby)	African-American	Feeding and settling an unsettled infant	Not described	Author-Results also highlight that infants of full bed-sharing mothers had less negativity than infants of non-bed-sharing mothers. The study findings suggest that mother-infant bed-sharing practices may positively impact later infant behavior and affect within the mother-child relationship. If replicated, this knowledge may help health care providers and parents make more informed decisions about sleep locations to improve the health and well-being of both parents and children.
Louis-Jacques, A. F., Bartick, M., Awomolo, A., Zhang, J., Feldman-Winter, L., Leonard, S. A., Meek, J., Mitchell, K. B., & Crowe, S. (2024). Bedsharing among breastfeeding physicians: Results of a nationwide survey.<br>USA	546 physicians and medical students who birthed children from October 2020 through August 2021	Quantitative-online survey was adapted from surveys administered by the Centers for Disease Control and Prevention	Exploratory	Breastfeeding<br>Better/more sleep<br>Bonding/Attachment/Relationship<br>Comforting for infant and/or mother/parent<br>Monitoring/safety/protection<br>Tradition (Family)	Breastfeeding-protective	Recommendations to not co-sleep not consistent with the lived experiences of the physicians in this sample.<br>Of those respondents who bedshared, 52% did not report it to their child’s healthcare provider and 19% did not answer the question	Not described	Not described
Luijk, M. P. C. M.,	5095 mothers at 2 months	Prospective cohort	neutral	Breastfeeding		Reactive-infant temperament, anxiety,	Not described	Not described

Mileva-Seitz, V. R., Jansen, P. W., van Ijzendoorn, M. H., Jaddoe, V. W. V., Raat, H., Hofman, A., Verhulst, F. C., & Tiemeier, H. (2013). Ethnic differences in prevalence and determinants of mother–child bed-sharing in early childhood.<br>Netherlands	and 5361 mothers at 24 months a population-based multiethnic (Dutch, Turkish and Moroccan, and Caribbean)	design-questionnaires and medical records		Tradition (Culture)<br>Infant preference/Difficult temperament<br>Environmental (no cot, no room)		wakefulness, culture that values autonomy and independence		
MacFarlane, M., Thompson, J. M. D., Mitchell, E. A., Lawton, B., McLardy, E. M., Jonas, S. D., Tepania-Palmer, G., Roa, T., Warren, G., & Jowsey, T. (2021). Pēpē-infant sleep practices and sudden unexpected death in infancy in Aotearoa New Zealand<br><br>NZ	Thirty māmā participated in the study (Figure 1), of whom 17 (57%) were Māori. Non-Māori māmā identified as Pasifika (n = 9, 30%) and European, including New Zealand/Other, and Asian (n = 4, 13%)	qualitative face to face interviews	exploratory. Bed-sharing is the cultural norm and expectation	Breastfeeding<br>Comforting for infant and/or mother/parent<br>Monitoring/safety/protection<br>Better/more sleep<br>Exhaustion/Fatigue<br>Bonding/Attachment/Relationship<br>Convenience/Ease<br>Tradition (Culture)<br>Infant preference/Difficult temperament<br>Crying (unsettled baby)	Māori and Pasifika<br>smoke exposure	Safety concerns-suffocation, rolling on baby<br>(Cousin's baby dies from a partner rolling on infant in bed)<br>Unintentionally falling asleep due to exhaustion (felt extremely bad and learned her lesson)<br>Grandparents falling asleep with baby on sofa against parent's wishes<br>Unsettled baby, won't sleep in recommended positions will sleep in bed<br>The need for quality maternal sleep	being a light sleeper<br>propping baby up on a pillow near her head to ensure his safety during sleep<br>Used a Pēpi-pods® at night but bed-shared for day naps with baby having own space by being separated by a pillow<br>Development of a whole family safer sleep plan to apply in real-world situations	A shared commitment between māmā, partners, and whānau<br>members to practice safe sleep as prescribed by māmā was important for Māori and non-Māori māmā. Based on personal experience of being home alone, exhausted, and breastfeeding in bed while her partner worked shifts, Tara (Participant 11, Māori) recommended whānau develop a plan outlining how, collectively, whānau and friends will apply safe sleep theory in real-world situations.<br>Author-Service providers are encouraged to respond to the lived experiences and cultural realities, values, and beliefs of māmā when designing and delivering effective interventions for the prevention of SUDI.<br>…partners, and their wider whānau have the capacity and capability to routinely practice safe sleep with their treasured pēpē, and some māmā may benefit from receiving individualized, strengths-based support.
Mathews, A., Joyner, B., Oden, R., Alamo, I., & Moon, R. (2015). Comparison of Infant Sleep Practices in African-American and US Hispanic Families: Implications for Sleep-Related Infant Death.<br><br>USA	422 African–American and 90 Hispanic mothers	cross-sectional, multimodal (surveys, qualitative interviews-focus groups or individual	exploratory but with a risk elimination tone	Monitoring/safety/protection<br>Convenience/Ease	African American<br>CALD<br>Lower SES<br>Smoke exposure	Safety concerns need to closely monitor baby who will only sleep prone-''He won't lay on his back, he won't lay on his side, but he'll lay on his stomach…And the things is, yea, and it's like if I lay him on his stomach, it's like ok, I gotta go in here and run in here, jump to make sure he is ok…so he sleeps with me.'' (28-year-old single lower SES mother of 2 month old)"<br>No other space<br>African American infants were more likely to be placed prone, bed-share and be exposed to smoke<br>African American mothers more knowledgeable about AAP recommendations but less adherent to them than Hispanic mothers with less knowledge	Not described	Not described
McIntosh, C., Trenholme, A., Stewart, J., & Vogel, A. (2018). Evaluation of a sudden unexpected death in infancy intervention programme aimed at improving parental awareness of risk factors and protective infant care practices.<br><br>NZ	240 Māori and Pacifica women-112 intervention group (101 for full intervention), 110 control group	randomised controlled trial	risk minimisation? But still aiming to stop shared sleep	Breastfeeding<br>Environmental (no cot, no room)	Māori and Pacifica<br>smoke exposure<br>low birth weight<br>congenital airways issue<br>prior SIDS of sibling	No baby bed<br>Baby outgrew Pēpi-pods® by around 8 weeks. Reduction in Pēpi-pods® use from 2 months' age is concerning because SUDI risk is still high at this age. Support to find and use a follow-on baby bed as the baby grows is important.<br>Several families were concerned about the safety of baby in the Pēpi-pods® when used around preschool children.<br>50% of babies were bed-sharing at least some of the time at follow up	Free provision of a Pēpi-pods® (in-bed portable Sleep Space)	Author-New Zealand data covering the period of this study suggest a significant reduction in SUDI for areas with a safe sleep space programme especially for Maori. This is perplexing given the lack of significant effect that this and Baddock et al.'s study of Wahakura demonstrated on frequency of bed-sharing and suggests that it is possible that there are other aspects of the safe sleep space programme that are influencing the SUDI rate. Perhaps more knowledge of bed-sharing risk is resulting in greater parental consideration of hazards in the bed-sharing environment.<br>Safe sleep programmes need to be targeted accurately to the level of risk and provision of culturally and age appropriate baby beds is required to provide the opportunity to safely sleep infants.
Moon, R. Y., Mindell, J. A., Honaker, S., Keim, S., Roberts, K. J., McAdams, R. J., & McKenzie, L. B. (2024). The Tension Between AAP Safe Sleep Guidelines and Infant Sleep<br><br>USA	25 US based mothers	survey and virtual focus groups	risk elimination	Comforting for infant and/or mother/parent<br>Better/more sleep<br>Exhaustion/Fatigue<br>Infant preference/Difficult temperament<br>Crying (unsettled baby)<br>Disagree with danger<br>In balance of risks, felt bed-sharing safer		Infants needing closeness and comfort to fall and stay asleep<br>Infant care cycle is exhausting<br>ABCs of safe sleep contributes to less sleep<br>Waking a lot seen to signify baby's dislike or discomfort in their sleep environment-mattress too hard, uncomfortable on back<br>Know the rules but when you're sleep	Some use Safe Sleep 7 guidelines from La Leche League International<br>Use of 'boppy' pillows in bed<br>Make sure there is nothing that could cover baby's face<br>Start in Pack 'n' Play, wakes every 5-20 minutes for a few hours then they move to the couch-"My couch just looks crazy. I have blankets shoved into all of the cracks, so there aren't any cracks to fall	When asked about how they decided between their infant sleeping well and sleeping safely, mothers frankly stated that the ABCs of Safe Sleep were unrealistic. Some were skeptical that the ABCs are the only safe option. Others chose to follow alternate guidelines, such as the Safe Sleep Seven by La Leche League International.<br>Still other mothers prioritized different aspects of the infant's sleep environment and tried to minimize what they perceived as most dangerous (eg, suffocation or

						deprived you do whatever you have to do to get baby to sleep<br>Prioritized minimising what they perceived as most dangerous-suffocation and falls	into, and with one of those firm lumbar support pillows that I put on the inner edge, and he sleeps on top of that. I stay on the like outer edge of the couch, and then I just make sure he's on his back and kind of turn his head to the side. Then he's comfortable, and he won't move out of that position all night until I put him back in the pack 'n play in the morning, so I can go to work."<br>Use of pillows to prevent falls	falls. Mothers were also reassured when they were not following the ABCs if they had received this advice from trusted sources in their social network. No mothers expressed concern that these alternate strategies placed their infant at higher risk for SUID.
Morrison, T. M., Standish, K. R., Wanar, A., Crowell, L., Safon, C. B., Colvin, B. N., Friedman, H., Schiff, D. M., Wachman, E. M., Colson, E. R., Drainoni, M. L., & Parker, M. G. (2023). Drivers of decision-making regarding infant sleep practices among mothers with opioid use disorder<br><br>USA	23 mothers with an Opioid Use Disorder (OUD)	Qualitative interviews and thematic analysis	risk elimination	Comforting for infant and/or mother/parent<br>Better/more sleep<br>Exhaustion/Fatigue<br>Infant preference/Difficult temperament<br>Crying (unsettled baby)	Opioid Use Disorder	Mothers described how their infants' withdrawal symptoms contributed to maternal stress and exhaustion = unintentionally falling asleep with baby<br>Lack of robust social support systems to help care for their infant, including establishment of infant sleep routines. Some mothers lived on their own with no support. Other mothers lived with or were co-parenting with an individual with a history of substance use disorder and felt they could not rely on this individual for infant care, including establishment of infant sleep routines, as this could impose too much stress on the individual. For example, one mother commented, "My husband suffers from addiction and he…wasn't doing well so I was like solo here.<br>Regarding consequences for bed-sharing in residential facilities, mothers explained that they could be "kicked out" of the facility and/or reported to child protective services for too many bed-sharing incidents. One mother recalled her experience with bed-sharing with her infant in a residential home, stating: "I had so many co-sleeping [bed-sharing] incidents, they [residential home staff] did file a 51 A [a report for child abuse or neglect] with DCF [child protective services]."	Falling asleep on couch-putting pillows on floor in case baby falls off<br>Sleeping baby on a boppy pillow that has been further propped by bean bag cushions to mitigate risk of baby rolling<br>Pillows around baby on adult bed to stop them rolling off	Authors-Many mothers acknowledged the safety risks with certain sleep practices and then engaged in certain behaviors to minimize such safety risks to their infant. Overall, mothers weighed this array of factors to make decisions about infant sleep practices that they believed were in the best interest of their infants and themselves. Our findings demonstrate unique experiences of mothers with OUD that may inform the development of tailored safe sleep interventions to reduce SUID in this infant population at high risk.
Murray, L., Tran, T., Thang, V. V., Cass, L., & Fisher, J. (2018). How do caregivers understand and respond to unsettled infant behaviour in Vietnam? A qualitative study.<br>Vietnam	21 Vietnamese mothers	Semi-structured qualitative interviews	exploratory. Bed-sharing is the cultural norm and expectation	Tradition (Culture)		Baby's head covered and many layers for sleep.<br>Fathers and siblings also in bed<br>The findings from this study illustrate that infants in Central Vietnam most commonly live with multiple adults of different generations, who share care provision, in particular during the day	Not described	Author-Educational interventions about the amount of sleep young infants require, interpreting infant cues, and settling strategies may be helpful for assisting new parents to create a household environment that is optimal for infant sleep. However, such interventions should include carers from multiple generations, address traditional understandings of the reasons infants cry and acknowledge that multiple family members (including other children) often sleep in the same bed.
Osei-Poku, G. K., Mwananyanda, L., Elliot, P. A., MacLeod, W. B., Somwe, S. W., Pieciak, R. C., & Gill, C. J. (2022). Assessing infant sleep practices and other risk factors of SIDS in Zambia: a cross-sectional survey of mothers in Lusaka, Zambia.	478 mothers in Lusaka Zambia	qualitative cross-sectional survey	risk elimination	Tradition (Culture)		89.5% of respondents indicated that they share a bed with the infant during sleep, 73.0% preferred putting their baby on its side, and 19.9% preferred the prone position. Only 6.7% of respondents described using the safer, supine position.<br>The type of mattress babies slept on was also reported to be mostly used (68.6%, 328/478) and soft (41.6%, 199/478).	Not described	Not described
Osei-Poku, G. K., Mwananyanda, L., Elliott, P. A., MacLeod, W. B., Somwe, S. W., Pieciak, R. C., Hamapa, A., & Gill, C. J. (2023). Qualitative assessment of infant sleep practices and other risk factors of sudden infant death syndrome (SIDS)	35 mothers in Lusaka Zambia	Qualitative, focus groups	risk minimisation. Bed-sharing is the cultural norm and expectation	Breastfeeding<br>Comforting for infant and/or mother/parent<br>Monitoring/safety/protection<br>Better/more sleep<br>Tradition (Culture)<br>Environmental (no cot, no room)		Bed-sharing the cultural norm<br>Inability to afford a separate room or bed (socioeconomic challenges).<br>Avoiding supine positioning for fear of aspiration or choking.<br>NOT BED-SHARING RELATED but relevant-Our findings suggest that preventing respiratory infections such as pneumonia likely takes precedence over preventing SIDS in cultures where such	Being vigilant was the best strategy to prevent such deaths. Most suggested frequently checking on the infant when they are sleeping, rarely leaving the infant to sleep alone, and being alert to the presence of the infant in bed as some strategies to reduce SIDS and smothering deaths. "…. The mind is alert. We know that there is the baby here [in same bed]. When the father is	Authors-In this population, the convenience of breastfeeding and the need to keep the infant safe motivated the need to bed-share. Strategies that make bed-sharing and breastfeeding safer may be worthwhile in these Zambian communities.<br>In our opinion, any behavioral change campaigns that result would benefit from a high degree of community input and sensitization, ideally guided by a community-based participatory research strategy to generate evidence that is not just robust but that will be

among mothers in Lusaka, Zambia.						infections are prevalent.<br>Fathers in bed	on this side, you know that the baby is in the middle, so when turning, you are careful. The mind is always alert"<br>All participants reported no tobacco use during pregnancy, citing possible neonatal nicotine addiction, stillbirth, and even unexplained sudden infant death as reasons for choosing not to smoke during pregnancy.	accepted by the community.
Pease, A., Ingram, J., Blair, P. S., & Fleming, P. J. (2017). Factors influencing maternal decision-making for the infant sleep environment in families at higher risk of SIDS: a qualitative study.<br><br>UK	20 mothers from a deprived area of Bristol, UK	Semi structured interviews	risk minimisation	Exhaustion/Fatigue<br>Short naps/Occasional	Three or more measures of increased risk of SIDS-young maternal age, smoking during pregnancy, three or more children, and a measure of deprivation	Disrupted routines<br>Advice that didn't seem credible or went against the mother's instincts.<br>Weighing up risks in balance<br>Falling asleep unintentionally-'A few times I had fallen asleep with him on me, not meaning to in the early days, and woken up in a panic like oh my God have I rolled on him? But then you're like no he's perfectly asleep, you haven't moved, everything is fine, and you doze back off again.'<br>While feeding-'And sometimes, you do, like nod off, and like, like, you wake up, and you think like… When I was doing, like, the bottle feeding and stuff in the middle of the night, I just, like, used to feel myself, like, feeding him and just going like that, and like trying to wake myself up. So, like, sometimes you just don't even realise, but, yeah, it is a bit worrying.'	Perceived increased maternal awareness//'mum sleep' 'When I've got a baby in my arms (and asleep) for some reason, I don't know why, I'm half and half, and I can hear things, and I know that she's fine. If she moved on me I can feel it because I'm half and half.'	Mothers felt the style of safe sleep advice was inappropriate, with one mother describing it as 'condescending' and another as being 'lectured'. Mothers said they felt didactic approaches gave little time for absorbing the information or asking questions. Some mothers described how health professionals had not taken the time to discuss safe sleep, as the mother already had children and it was taken for granted that they would have heard the advice before. Mothers often described pressure from health professionals to comply with safe sleep messages, and this was connected to a lack of understanding as to why safe sleep messages are important.<br>Mothers talked about lists of 'do this' and 'do not do this' as unhelpful in helping them make decisions, stating that they would prefer a more individual approach using friendly conversations with people they trust.<br>Mothers found didactic approaches to advice unhelpful, and wanted to know why and how these messages keep babies safe. Mothers in this study coped with the stress of looking after a young baby by going against the advice every now and then and felt that this was normal and justified. Mothers used alternative strategies to reduce the risk, which were more about helping them feel like they were protecting their infants;<br>Author-Safer sleep messages should be tailored to fit with the lived realities of mothers, especially those at higher risk. The traditional list of 'do's' and 'don'ts' was not well accepted by this group. Interventions that seek to influence this higher-risk group should acknowledge mothers' own protective instincts and consider their beliefs and understanding behind the safer sleep messages if they are to be effective and encourage this group to change.
Pease, A., Turner, N., Ingram, J., Fleming, P., Patrick, K., Williams, T., Sleap, V., Pitts, K., Luyt, K., Ali, B., & Blair, P. (2023). Changes in background characteristics and risk factors among SIDS infants in England: Cohort comparisons from 1993 to 2020<br><br>UK	Infants-138 SIDS deaths in 2020 compared with 402 SIDS deaths and 1387 age-equivalent surviving controls	Cohort of SIDS in 2020 compared with a combined analysis of two case-controlled studies	risk minimisation	Change in routine (away from home)	low birth weight, premature, male infants, smoke exposure during pregnancy and after, socioeconomically deprived families, prone, non-sober parent, sleeping on a sofa, times of disrupted routine	change in routine	Our specific recommendations based on these findings, are:<br>1. National SIDS risk reduction campaigns should focus on renewed efforts to emphasise the need for all babies to be put down for sleep on their backs and avoid hazardous co-sleeping; in particular, to avoid using sofas or consuming alcohol or drugs before bed-sharing.<br>2. Families with infants at increased risk should be provided with targeted intensive support, particularly in the first few months of a baby's life, to address the barriers to following safer sleep advice, and plan for infant sleep safety during times of disruption to the normal routine.<br>3. Annual monitoring by CDOPs and the NCMD should include reporting on background characteristics of families affected by unexpected infant deaths, track sleep environment risk factors present in the deaths each year and provide geographical 'heat maps',	Authors-Certainly, the finding that almost half of the deaths occur in hazardous co-sleeping circumstances should be a call to all those involved in improving the uptake of safer sleep advice to find ways to support families to reduce the risks for their infants from these situations. Sofa sharing and bed-sharing in the presence of an adult who smokes, has consumed alcohol or taken drugs should be the first priorities. The finding that changes to the infant care routine are increasingly prevalent in deaths, and that combinations of risk factors make the situation worse,27<br>suggests that prevention<br>efforts may also benefit from interventions that directly focus on planning for safety during times of disruption to the normal routine. Our work with vulnerable families has shown that parents appreciate individually tailored advice that provides reasons, giving the how and why certain situations can increase risks for a baby
Pretorius, K., Choi, E., Kang, S., & Mackert, M. (2020). Sudden infant death syndrome on	526 mothers on Facebook (undescribed locations)	Qualitative data-textual analysis	risk elimination	Breastfeeding<br>Comforting for infant and/or mother/parent<br>Monitoring/safety/protection		Fear of co-sleeping wanting to learn more because husband want to share sleep (had been 'freaked' out about co-sleeping in hospital)	mother allowed her infant to sleep in bed on a pillow because the mother could not rest "without [the baby] being near," despite knowledge that such practices	Informational support was evident in the following discussions among mothers: (1) asking questions about SIDS, infant sleep, or baby products; (2) sharing personal experiences of provider communication

Facebook: Qualitative descriptive content analysis to guide prevention efforts<br><br>USA				Convenience/Ease<br>Infant preference/Difficult temperament<br>Disagree with danger<br>Closer monitoring when baby is sick/post immunisations/teething/reflux<br>Needed due to (Mother's) injury/caesarean<br>In balance of risks, felt bed-sharing safer		Mother's caesarean making it hard to get up and tend to baby, closer monitoring for a baby with reflux, many mothers in the group were aware of safe sleep recommendations but chose not to follow them because they were not feasible. Sleep practices changed from night to night.	were not recommended. Mothers felt confident they would "wake up the moment anything [happened]" or mentioned that they would never roll onto their infant when bed sharing. Another mother who was bed sharing explained that she was not worried about suffocation as the blanket did not reach the infant's head and that she and her husband do not move throughout the night.	regarding SIDS or safe sleep; (3) sharing personal definitions or beliefs regarding SIDS or safe sleep (including discussions on vaccines and SIDS); and (4) sharing informational sources for SIDS or safe sleep. Overwhelmingly, the information shared demonstrated misinformation and inaccurate use of terminology. Another finding is that infant sleep practices are not straightforward; safe sleep and unsafe sleep are best described on a continuum. Mothers alter infant sleep practices based on the infant and family's needs. Many mothers in the group were aware of safe sleep recommendations but chose not to follow them because they were not feasible. This finding supports a prior study's conclusion that parental motivation to bed-share trumped known risks of unsafe sleep
Rudzik, A., & Ball, H. (2016). Exploring Maternal Perceptions of Infant Sleep and Feeding Method Among Mothers in the United Kingdom: A Qualitative Focus Group Study.<br>UK	39 mothers in the UK	Qualitative analysis-focus groups	exploratory	Breastfeeding<br>Better/more sleep<br>Convenience/Ease	Formula Feeding	Need for sleep, exhaustion, fatigue-Use of both formula feeding and cry it out sleep training to attain a baby sleeping through the night on their own against safe sleep advice to room share and breastfeed.<br>Those who did breastfeed were much more likely bed-share to manage their baby's nighttime needs.<br>"I find I get more sleep, if I'm honest, than I did [when I used formula]…Even though he's woken up more, if he's having a night where he wants to nurse a lot I'll put him in bed with me and I'll just sleep and he just latches on when he wants to and it doesn't really interrupt my sleep a great deal. Whereas the other [older] two, when you're bottle feeding them you've got to kind of sit up with them, hold a bottle in their mouth, so you have to be up and you have to be awake.:	Not described	Not described
Sahud, H., Berger, R. P., Hamm, M., Heineman, E., Cameron, F., Wasilewski, J., Griffin, A., & Muniz, G. B. (2025). Understanding parental choices related to infant sleep practices in the United States using a mixed methods approach.<br>USA	21 parents who had practiced non-recommended sleep methods with their infant and had or had not experienced an undesirable sleep event such as a fall.<br>85% mothers<br>14% fathers	Mixed Methods-one-on-one phone interviews	Exploratory-Risk minimisation	Comforting for infant and/or mother/parent<br>Infant preference/Difficult temperament<br>Monitoring/safety/protection<br>Crying (unsettled baby)<br>Better/more sleep<br>Exhaustion/Fatigue	Mixed-<br>Priority population-African-American	Despite parents universally understanding safe sleep recommendations and all had separate space available they often did not use these because either infant/ parent was not comfortable with that environment.<br>Perceived risk of falls focus of their risk mitigation actions<br>Reluctant or did not disclose shared sleep to HP.<br>Sleep accident happened while trying to comply- fell asleep in chair and dropped infant.	Perceived risk of falls focus of their risk mitigation actions<br>mitigation strategies focused on the placement of the baby in the center of the bed and not using pillows or blankets when sleeping with the child, many parents referred to themselves as being "light sleepers", feeling confident that they would wake up if the baby became distressed, moved, or made noise<br>Falls/ roll- Cushioned border, ottoman next to couch (on couch as lower than bed to reduce fear of baby falling from height), parents either side, no pillows or bedding	Recommendations 'not realistic or practical'<br>or practical- "Um, 'cause I've tried putting her in the pack and play at night and she wakes up right away screaming and then, you know, you're back and up all night… Put the baby in their crib, you know, but in real life, you know, it's aren't—it's not as easy as that." "It all sounds great in theory. I just don't know how to put it into practice from a realistic, practical standpoint." "I don't think' it's real – it's not always realistic….I think they [primary care providers] need to be more realistic about, you know, when, uh, parents are tired or caregivers are tired and, um, you know, they don't always do a hundred percent of what is recommended."<br>"…I can try not to put him in bed with me. That's fine, but I can't guarantee that it's not gonna' happen on nights when we're tired and people need to get sleep."
Salm Ward, T. C., Miller, T. J., & Naim, I. (2021). Evaluation of a multisite safe infant sleep education and crib distribution program<br>USA	615 mothers (pre-and post) and 66 follow-ups	Matched pre and post-test cohort design with follow up survey	risk elimination	Breastfeeding<br>Exhaustion/Fatigue<br>Environmental (no cot, no room)		Unintentionally falling asleep on sofa, chair or in bed while feeding even after intervention (provision of cot)<br>Lack of crib?<br>Toddler siblings in bed	Free crib provision? No solutions to assist in shared sleep safety	The results suggest that participating in the education program was associated with increased knowledge and intended adherence, but that these changes were not maintained at follow-up. These results are in line with the research literature that finds a difference in intentions and actual practices after the baby is born.
Salm Ward, T. C. (2023). "Things changed very quickly": Maternal intentions and decision-making about infant sleep surface, location, and position<br>USA	Mothers and fathers-22 families (20 mothers and 2 mother–father dyads)	Qualitative semi-structured interviews	risk elimination with a dash of minimisation	Breastfeeding<br>Comforting for infant and/or mother/parent<br>Monitoring/safety/protection<br>Bonding/Attachment/Relationship<br>Infant preference/Difficult temperament<br>Crying (unsettled baby)<br>Disagree with danger<br>Maternal instinct		Tried other sleep surfaces but baby doesn't settler and sleep as well as they do in bed with, the parent.<br>Unintentionally falling asleep feeding on the couch<br>Need for adult sleep when baby won't sleep alone	Most families who reported bed-sharing described efforts to reduce the perceived risks of bed-sharing, for example, risk of suffocation, overlay, or falling. Some described keeping loose or soft items, such as pillows or blankets, away from baby's sleep space. Some paid specific attention to where baby was positioned in bed, for example, when bed-sharing with a partner, they placed baby on the outside of the bed, not near the partner. Others placed the infant above their shoulder in the bed, and one placed the infant perpendicular to the mother	Author-While advice was often cited as one consideration, it was not the ONLY factor considered, and often the participants would note that they would weigh the other factors described previously ("trying things," "safety concerns," and "what's comfortable for me") over the advice of others.<br>providing additional details about factors that increase risk in bed-sharing (such as those noted in the 2022 AAP recommendations, for example, with a current smoker, on a soft surface, with soft bedding, and with infants under age 5 months)2 could be helpful for families who choose to bed-share. Many mothers in this sample were trying to be careful by attempting to address potential risks. Some parents' attempts

								(horizontally on the bed instead of vertically) to reduce the risk of infant rolling off the bed. A few others arranged their own body around the infant to avoid rolling onto the infant, for example, placing their legs or arms so as to not roll over or reported not moving during sleep. A few blocked one side of the bed to prevent the infant from falling out of bed, for example, using furniture (such as portable crib) pushed up against the bed, placing a bedside sleeper between the bed and wall so the infant would roll onto it versus falling, or using bed rails on the adult bed. A few mentioned avoiding alcohol or drug use when bed-sharing, and a few reported using hypervigilance, always watching, or light sleeping to reduce risk. A few reported propping the infant's head on the mother's arm to avoid perceived risk of choking or of mother rolling onto infant. A few placed pillows around the infant or on the floor to protect infant from perceived risk of rolling and falling out of bed. A few also sat upright in a recliner or propped up in bed while holding infant to avoid falling asleep in a bed-sharing situation or placed the infant on their chest to monitor the infant's breathing—in those situations, however, the caregivers often reported falling asleep.<br>Risk reduction efforts that may increase risk	somewhat aligned with AAP recommendations (e.g., remove soft items),2 while others attempted to reduce perceived risk in ways that may have actually increased suffocation risk, such as placing pillows around infant.
Shimizu, M., Park, H., & Greenfield, P. M. (2014). Infant sleeping arrangements and cultural values among contemporary Japanese mothers.<br><br>Japan	51 Japanese mothers	Qualitative analysis of comments on a parenting forum	neither. Bed-sharing is the cultural norm and expectation	Breastfeeding<br>Comforting for infant and/or mother/parent<br>Monitoring/safety/protection<br>Bonding/Attachment/Relationship<br>Convenience/Ease<br>Tradition (Culture)<br>Disagree with danger<br>Closer monitoring when baby is sick/post immunisations/teething/reflux		Co-sleeping is as frequent among Japanese mothers in 2008-2009 as it had been in the 1960s and 1980s however, honouring traditional infant care practices including shared sleep (soine) can be challenging for Japanese mothers as they construct their values for child rearing and gender roles in the current sociodemographic conditions.	"The definition of "soine" is unclear to me. Does it mean that a mother and a baby sleep together on the same bedding? I think that's scary. In my family, husband and I sleep on our own futon next to each other, and baby's futon was spread above my head. So this is not "kawa" [child sleeping between parents] position. But with this position, my husband and I are close to each other, and plus, I can see my baby's face. I wonder whether it's difficult to breastfeed a baby or change diapers when sleeping in a separate room. Just a simple question."<br>Own futon/crib next to parents.	"The definition of "soine" is unclear to me. Does it mean that a mother and a baby sleep together on the same bedding?
Shin, S. H., Choi, C., Shih, S. F., Tomlinson, C. A., & Kimbrough, T. (2023). A Hospital-Based Infant Safe Sleep Intervention and Safe Sleep Practices Among Young Women: A Prospective Longitudinal Study<br>USA	411 US women	Pre-post-test surveys-quantitative	risk elimination			Bed-sharing rates increased after the third postpartum month despite women reporting during the initial study period that they avoided bed-sharing. One possible explanation-infant development leads to parent's relaxing safe sleep. Also hypothesised that high room-sharing rates may lead to more bed-sharing that those who sleep infant in separate room.	"our results suggest that a hospital-based preventive intervention incorporating education and home visiting services can effectively improve safe-sleep practices by reducing accidental suffocation risks in the first six months of life through removing unsafe items from the sleeping areas. However, the intervention did not demonstrate the effectiveness in a reduction in bed-sharing. Furthermore, we still continue to see that underlying sociocultural factors influencing parents' decisions to continue to bed-share. Therefore, it is imperative that future research focuses on specific target groups and develop culturally appropriate intervention strategies that effectively reduce the use of unsafe	Not described

							sleep practices to reduce the rate of SUIDs and promote health equity across populations."	
Stiffler, D., Matemachani, S. M., & Crane, L. (2020). Considerations in Safe to Sleep messaging: Learning from African-American mothers.<br><br>USA	15 African American mothers	Qualitative focus groups	risk elimination but exploring risk minimisation	Breastfeeding<br>Monitoring/safety/protection<br>Exhaustion/Fatigue<br>Convenience/Ease<br>Infant preference/Difficult temperament<br>Crying (unsettled baby)<br>Needed due to (Mother's) injury/caesarean	African-American	Baby won't settle or stay asleep on back. Grandmother's insisting baby should sleep on stomach in bed-One mother stated, "My culture has the baby sleep with us, my mom helped me a lot with my first daughter, but she insisted that the baby should sleep with me and on her tummy." Another said, "My mother called it "New mommy stuff," (following safe sleep guidelines) that's not for me." "For first-time moms, you believe everything and try to follow everything, but with each subsequent child, you know it won't work, so you don't try. When she slept on her belly, she slept for hours, but on her back, she was fussy all night. The postpartum depression kicks in because of lack of sleep. If you don't get any sleep, you are cranky to everyone else. You don't get any sleep, when you put your baby on its back, I have to stay up with the baby. She's crying, I'm crying. Where do you draw the line?"	These focus group participants heard the safe sleep recommendations but had difficulty following them.	We asked the mothers, "How would they change the message? How could the message be year of the baby's life, you should have them keep getting the message—not just in the hospital." Another said, "People are more comfortable with their own telling what happened to them." One mother said that she had had two different providers tell her two different things, so consistency in the message is very important. Other suggestions included:<br>• Have more details, not just a pamphlet • Make sure you tell everyone: parents, mother, father, grandparents, everyone<br>• Stress the statistics about the higher incidence among African–American infants<br>• Have a person give the message, not just a video • Dads frequently watch infants, so they need to be educated • People who look like me telling what happened to them • They need to give examples—real people giving real-life examples • Throughout the first year of the infant's life, you should keep getting the message—not just in the hospital
Tully, K., Holditch-Davis, D., & Brandon, D. (2015). The Relationship Between Planned and Reported Home Infant Sleep Locations Among Mothers of Late Preterm and Term Infants.<br><br>USA	56 mother infant dyads-26 late preterm and 30 term	Qualitative semi-structured interviews	risk minimisation	Breastfeeding<br>Comforting for infant and/or mother/parent<br>Monitoring/safety/protection<br>Better/more sleep<br>Exhaustion/Fatigue<br>Bonding/Attachment/Relationship<br>Convenience/Ease<br>Infant preference/Difficult temperament<br>Crying (unsettled baby)<br>Environmental (no cot, no room)<br>Short naps/Occasional	Premature	Women in both groups said that they planned not to bed share at home due to fear for infant safety-fears of suffocation, overlay, falls etc.<br>Fear of spoiling baby<br>Significantly more women reported that they bed shared with their infant during the first postpartum month (21 of 44, 47.7 %) than said that they had planned to do so (6 of 56, 10.7 %), X2 = 15.3, df= 1, p\.0001.<br>Late preterm and term groups did not differ in unplanned bed sharing. Further, the proportions of any parent-infant bed sharing by 1 month postpartum, regardless of intentions, did not differ by childbirth group.<br>All women who planned on bed sharing reported doing so. Women in the late preterm and term groups offered similar reasons for bed sharing, with nighttime infant fussiness being the primary factor. Other reasons for bed sharing were maternal preference/emotional closeness, reassurance of infant wellbeing, convenience with nighttime feeds, early morning rest, daytime naps, and lack of a bassinet (term mother).<br>One of the women who bed shared did so because she was told the baby (born late preterm) had to be upright for a prolonged period after feeding.	A mother who reactively bed shared with a late preterm infant propped on pillows. She did this to comply with the pediatrician's advice to position the baby upright for an extended period after feedings and her need to achieve this arrangement in a way that permitted maternal sleep. Maternal decision making for high-needs infants may be impaired by fatigue from managing health challenges over time.	Authors-Professional guidance that works with mothers, acknowledges their challenges around infant sleep locations and provides evidence-based guidance on potential hazards may help to enable both safe infant environments and maternal rest.<br>Conversations among health care providers and families may advance understanding of infant sleep location recommendations away from the 'letter of the law' to the 'spirit.'
Weil, L. E. (2020). Prevention of unintentional, sleep-related infant deaths: Current sleep practices, caregiver beliefs, and promotion of safe sleep practices through effective preventive interventions<br><br>USA	12 young mothers from Cook County, US living in transitional living programs for young mothers	Self-report surveys + Qualitative focus groups	risk elimination	Breastfeeding<br>Comforting for infant and/or mother/parent<br>Monitoring/safety/protection<br>Better/more sleep<br>Exhaustion/Fatigue<br>Bonding/Attachment/Relationship<br>Convenience/Ease<br>Infant preference/Difficult temperament<br>Disagree with danger	Young Maternal Age	Individual needs of the child: "She wouldn't fall asleep unless she was on top of me. I would lay on the edge [of the bed] on the open part and she would lay on the edge closest to the wall, so she can't fall."<br>Confusion about the terms co-sleeping and bed-sharing-used AAP definitions where co-sleeping is referring to room-sharing not sharing a sleep space.<br>It is believed that some safe sleep preventive campaigns that were fear-based (e.g., Milwaukee's campaign with a knife in bed with an infant) may have prevented caregivers from openly	Solution to address risk of falling-"She wouldn't fall asleep unless she was on top of me. I would lay on the edge [of the bed] on the open part and she would lay on the edge closest to the wall, so she can't fall."	several participants spoke to the requirements being rigid and not incorporating the individual needs of the mother and child.<br>Some mothers believed loss-framed materials would resonate more with caregivers: "It would be more effective because it is actual real life. You are actually hearing from the parent to make you realize that 'wow this could be me if this is what I am doing.' I could be her. And actually see the pain and the tears in her eyes." However, other mothers expressed that the loss-framed messaging is too negative and may be a "turn off" to some caregivers. Additionally, participants noted that the loss-framed materials did not provide any information about the safe way of sleeping with a baby. Thus, acknowledging that even if the loss-framed

						sharing about their use of unsafe sleep practices, rather than opening a dialogue that may have encouraged use of alternative sleep methods (Peacock et al., 2017).		material resonated with a caregiver, he/she may still not have the knowledge of how to sleep safely with an infant., many caregivers were unclear about the difference between SIDS, asphyxiation, and suffocation. Preventive education and healthcare messaging have tended to emphasize information about SIDS risk reduction and have been less focused on discussing prevention of unintentional deaths such as asphyxiation and suffocation (Mathews et al., 2016). This difference in terminology is an important distinction, as parents in our focus groups and prior studies have shown a low level of self-efficacy in regard to protecting their child from SIDS but a high level of self-efficacy with regards to preventing unintentional sleep-related death from asphyxiation This study shows that caregivers rely on technology and leverage internet applications for information about their children. Using social media platforms, Facebook in particular, may be an effective way to educate caregivers and reach a large audience. Social media education can easily be disseminated to target groups based on user demographics. An additional dissemination method is through doctor's appointments, as caregivers in this study reported medical providers as the most trusted source of information about infant safety. Caregivers also reported fear of judgment from medical providers and a desire for education to be tailored to the family's situation, rather than a one size fits all model. Training medical providers about educational approaches that will be most welcomed by caregivers may be a necessary next step in advancing effective preventive education.
Yuma-Guerrero, P. J., Duzinski, S. V., Brown, J. M., Wheeler, T. C., Barczyk, A. N., & Lawson, K. A. (2013). Perceptions of injury and prevention practices among pregnant and parenting teenagers.<br><br>USA	93 pregnant or parenting teenagers 9507% (n=89) mothers 4.3% fathers (n=4) who are also students in Texas USA	Semi structured focus groups	risk elimination	Breastfeeding<br>Comforting for infant and/or mother/parent<br>Monitoring/safety/protection<br>Bonding/Attachment/Relationship<br>Convenience/Ease<br>Environmental (no cot, no room)<br>Ease of feeding (not breastfeeding)	Teenage mothers	Conflicting advice. Multiple sources for sleeping information-older women close to the parent, health care providers, television, daycare, or written materials. Participants consistently described health care providers' sleeping advice as placing children in a crib, on their backs, and without blankets, pillows, or toys. Some participants found this information helpful, while others found it "ridiculous".<br>"Teenagers may not have developed all the skills needed ***to effectively parent without risk.*** Therefore, pregnant and parenting teenagers may need assistance for developing skills to prevent injuries within their complex and changing circumstances." They may also require assistance for educating others – family and social supports	Not described	Author's contribution-Need assistance to develop skills to prevent injuries in complex, ever changing circumstances<br>Need for support in educating other family members and social supports
Zoucha, R., Walters, C. A., Colbert, A. M., Carlins, E., & Smith, E. (2016). Exploring Safe Sleep and SIDS Risk Perception in an African-American Community: Focused Ethnography<br>USA	84% women and 16% male--19 African-American caregivers	Semi structured qualitative interviews	risk elimination	Comforting for infant and/or mother/parent<br>Monitoring/safety/protection<br>Bonding/Attachment/Relationship<br>Tradition (Culture)	African American	Need for close monitoring of breathing<br>Need for softness in bed for baby's comfort<br>Grandmothers and older women shown much respect and have a lot of influence though some of advice not always safe and this could be a source of conflict at times		Informants reported that they would appreciate working with nurses and nursing students on a variety of health care issues in the community. Informants shared ideas about partnerships that would help their community. Ideas such as training young mothers and grandmothers to serve as health educators, nurses going door to door, hosting community events such as a barbeque with a heath fair would work in their community. A common thread in all the data was that nursing intervention would be very welcomed regarding SIDS and safe sleep practice.

Australian College of Midwives. (2014). *Position Statement for Bed Sharing and Co-Sleeping*. . Australian College of Midwives. Retrieved 19 September 2024 from https://midwives.org.au/Web/Web/About-ACM/Guiding-Documents.aspx#:~:text=Midwives%20should%20be%20aware%20of,%20and

Blair, P. S., Ball, H. L., McKenna, J. J., Feldman-Winter, L., Marinelli, K. A., Bartick, M. C., & Academy of Breastfeeding, M. (2020). Bedsharing and Breastfeeding: The Academy of Breastfeeding Medicine Protocol #6, Revision 2019. *Breastfeed Med*, *15*(1), 5-16. https://doi.org/10.1089/bfm.2019.29144.psb

HealthEd. (2023). *Keep Your Baby Safe During Sleep - HE1228*. Ministry of Health. Retrieved 1st October from https://healthed.govt.nz/products/keep-your-baby-safe-during-sleep#:~:text=The%20full%20resource:%20Every%20year,%20too

Moon, R. Y. (2023). *How to Keep Your Sleeping Baby Safe: AAP Policy Explained*. American Academy of Pediatrics. Retrieved 21 February 2024 from https://www.healthychildren.org/English/ages-stages/baby/sleep/Pages/A-Parents-Guide-to-Safe-Sleep.aspx

National Health Service. (2025). *Safer Sleep*. NHS. Retrieved 26 July 2025 from https://www.nhs.uk/start-for-life/baby/baby-basics/newborn-and-baby-sleeping-advice-for-parents/safe-sleep-advice-for-babies/#co-sleeping-with-your-baby

National Institute for Health and Care Excellence. (2021). *Postnatal Care: NICE guideline [NG194]*. NICE National Institute for Health and Care Excellence. Retrieved 22 February 2022 from https://www.nice.org.uk/guidance/ng194/chapter/recommendations#bed-sharing

Queensland Clinical Guidelines. (2022). *Safer Infant sleeping*. Australia: State of Queensland Retrieved from http://www.health.qld.gov.au/qcg

Red Nose Australia. (2024). *Co-sleeping with your baby*. Red Nose Australia. Retrieved 21 February 2024 from https://rednose.org.au/article/Co-sleeping_with_your_baby

UNICEF UK. (2022). *Caring for your baby at night*. United Kingdom Committee for UNICEF. Retrieved 21 February 2024 from https://www.unicef.org.uk/babyfriendly/wp-content/uploads/sites/2/2018/08/Caring-for-your-baby-at-night-web.pdf
